# Proceedings of the Canadian Society of Allergy and Clinical Immunology Annual Scientific Meeting 2019

**DOI:** 10.1186/s13223-020-00445-x

**Published:** 2020-06-24

## Allergic rhinitis/asthma

### #1 IgG4 cross-reactivity induced by the SQ tree SLIT-tablet extends the immune modulating effect of the SQ tree tablet to the entire birch homologous group

#### Peter A. Würtzen^1^, Henrik Ipsen^1^, Pernille M. Grønager^1^, Maria A. Steffensen^1^, Marie Chantal Arseneault^2^, Kaare Lund^1^

##### ^1^ALK, Horsholm, Denmark; ^2^ALK Canada, Montreal, QC, Canada

###### **Correspondence:** Marie Chantal Arseneault

*Allergy Asthma Clin Immunol* 2020, **16(Suppl 1):**#1

**Background:** Allergic rhinoconjunctivitis is commonly caused by allergens from the birch homologous group, which includes birch, oak, hazel, alder and hornbeam, all characterised by having Bet v 1 homologous allergens that extensively cross-react. The current study characterized the ability of the SQ tree SLIT-tablet (containing birch extract) to induce immunological responses that cross-react with all species in the birch homologous group.

**Methods:** Blood samples were collected from adults who had moderate to severe rhinoconjunctivitis induced by birch pollen who were treated with the SQ tree SLIT-tablet for 24 weeks (ClinicalTrials.gov Identifier NCT02481856). Serum IgE (pre-treatment) and IgG4 (post-treatment) specific for birch, oak, hazel, alder, beech, chestnut and hornbeam were measured by standard ImmunoCAP. Correlation analyses and inhibition assays were performed to evaluate immunological cross-reactivity. The study was approved by the Ontario IRB, approval number 12069.

**Results:** The analyses demonstrated a high level of correlation between IgE reactivity (pre-treatment) between birch and alder, hazel, hornbeam and oak. The strongest correlation in IgE titers was observed between birch and alder (r = 0.98), and the weakest between birch and oak (r = 0.83). A strong correlation between IgE titers was also observed for beech, but not for chestnut. The analyses also demonstrated a high level of correlation between IgG4 reactivity (treatment-induced) towards birch and alder, hazel, hornbeam, beech and oak, with the strongest correlation between birch and alder (r = 0.95), and the weakest between birch and oak (r = 0.78). Inhibition experiments revealed that in the majority of patients, alder-, hazel- and oak-specific IgE and IgG4 reactivity was inhibited > 80% by birch allergen extract.

**Conclusion:** The data demonstrate a high level of cross-reactivity between birch-specific IgE and IgG4, and allergens from the birch homologous group, which suggests that the significant clinical effect determined previously for both birch and oak exposure may extend to the entire birch homologous group.

### #2 Dupilumab improved asthma control and health-related quality of life in patients with oral corticosteroid-dependent, severe asthma during the LIBERTY ASTHMA VENTURE study

#### Linda B. Ford^1^, Klaus F. Rabe^2,3^, Robert N. Wolfe^4^, Santiago Quirce^5^, Megan S. Rice^6^, Paul Rowe^7^, Ariel Teper^7^, Siddhesh Kamat^8^, Nikhil Amin^8^, Neil M.H. Graham^8^, Asif Khan^9^

##### ^1^Asthma and Allergy Center, Bellevue, NE, USA; ^2^LungenClinic Grosshansdorf (member of the German Center for Lung Research [DZL]), Airway Research Center North (ARCN), Grosshansdorf, Germany; ^3^Christian-Albrechts University (member of the German Center for Lung Research [DZL]), Airway Research Center North (ARCN), Kiel, Germany; ^4^Cedars-Sinai Medical Center, Los Angeles, CA, USA; ^5^Hospital La Paz Institute for Health Research (IdiPAZ), Madrid, Spain; ^6^Sanofi, Cambridge, MA, USA; ^7^Sanofi, Bridgewater, NJ, USA; ^8^Regeneron Pharmaceuticals, Inc., Tarrytown, NY, USA; ^9^Sanofi, Chilly-Mazarin, France

###### **Correspondence:** Linda B. Ford

*Allergy Asthma Clin Immunol* 2020, **16(Suppl 1):**#2

**Background:** Dupilumab, a fully human monoclonal antibody, blocks the shared receptor component for IL-4/IL-13, key drivers of type 2 inflammation in multiple diseases. In the USA, dupilumab is approved for patients aged ≥ 12 years with moderate to severe eosinophilic or oral corticosteroid (OCS)-dependent asthma and for patients aged ≥ 12 years with inadequately controlled, moderate-to-severe atopic dermatitis. In the phase 3 VENTURE study (NCT02528214), dupilumab 300 mg every 2 weeks, vs. placebo, reduced OCS use and severe asthma exacerbations, while improving FEV1 during the 24-week treatment period, independently of baseline eosinophil levels, in 210 patients with OCS-dependent, severe asthma. This pre-specified analysis assessed dupilumab’s effect on asthma control and health-related quality of life (HRQoL) in these patients.

**Methods:** Asthma control was assessed by 5-item Asthma Control Questionnaire (ACQ-5), where higher scores (range 0–6) indicate less control. HRQoL was assessed by Asthma Quality of Life Questionnaire (AQLQ), where higher scores (range 1–7) indicate better HRQoL. Changes from baseline were analyzed using mixed-effect models with repeated

**Results:** At baseline, dupilumab and placebo groups had mean ACQ-5 scores of 2.42 and 2.58 and mean AQLQ scores of 4.38 and 4.31. In the dupilumab group, ACQ-5 scores improved by Week 2 (LS mean change from baseline − 0.57, P = 0.002 vs. placebo) with further improvements by Week 12 (− 1.01, P = 0.001 vs. placebo), which were sustained to Week 24 (− 1.05, P = 0.002 vs. placebo). AQLQ scores improved by first assessment at Week 12 (0.76, P = 0.14 vs. placebo) and continued to improve to Week 24 (0.89, P = 0.008 vs. placebo). Similar trends were observed in patients who achieved ≥ 50% OCS dose reduction or discontinued OCS use. Overall, the most frequent treatment-emergent AE occurring in dupilumab- vs. placebo-treated patients was eosinophilia (14% vs. 1%).

**Conclusion:** Dupilumab vs. placebo improved asthma control and HRQoL in patients with OCS-dependent, severe asthma. Dupilumab was generally well tolerated.

### #3 Targeting the IL-5 pathway in eosinophilic asthma: a comparison of mepolizumab to benralizumab in the reduction of peripheral eosinophil counts

#### Arian Ghassemian^1^, Michael W. Tsoulis^2^, Harold Kim^1,3^

##### ^1^Division of Clinical Immunology and Allergy, Department of Medicine, Schulich School of Medicine and Dentistry, Western University, London, ON, Canada; ^2^Renaissance School of Medicine, Stony Brook University, Stony Brook, NY, USA; ^3^Division of Clinical Immunology and Allergy, Department of Medicine, Michael D.DeGroot School of Medicine, McMaster University, Hamilton, ON, Canada

###### **Correspondence:** Arian Ghassemian

*Allergy Asthma Clin Immunol* 2020, **16(Suppl 1):**#3

**Background:** Mepolizumab and benralizumab are biologics approved for severe eosinophilic asthma. Mepolizumab is an anti-IL-5 antibody while benralizumab is an anti-IL-5Rα antibody targeting the IL-5 receptor on eosinophils. Both therapies reduce oral steroid requirements and asthma exacerbations. However, no head-to-head studies have been published. We compared these agents in their efficacy in peripheral eosinophil reduction.

**Methods:** A retrospective chart review was conducted on patients with severe eosinophilic asthma who were approved for either IL-5 agent. Patients with noted non-compliance or those who were on fluctuating doses of steroids for non-asthma related illnesses were excluded. The last detectable eosinophil count for each patient prior to start of therapy was compared to the highest eosinophil count noted after therapy start.

**Results:** Thirty-six patients taking mepolizumab and 19 patients taking benralizumab met the inclusion criteria and had both pre-treatment and post-treatment eosinophil counts. There was no statistically significant difference between the age and sex of the patients taking mepolizumab versus benralizumab. The mean pre-therapy serum eosinophil count did not statistically differ between patients on mepolizumab (611.1 cells/µL) compared to benralizumab (526.3 cells/µL), p = 0.3222. While both therapies resulted in a significant decrease in eosinophil count (p < 0.0001); the mean decrease did not statistically differ between patients taking mepolizumab compared to those on benralizumab, p = 0.7420. Nonetheless, 100% of patients receiving Benralizumab had undetectable eosinophil counts post-therapy compared to 25% of patients receiving Mepolizumab (p < 0.0001).

**Conclusion:** Both mepolizumab and benralizumab are potent targets of the IL-5 pathway with the ability to significantly reduce peripheral eosinophil counts. While there is there is no statistical difference in the magnitude of eosinophil reduction offered by each agent, benralizumab is superior in decreasing peripheral eosinophil counts to 0 cells/µL.

*This study was approved by the Hamilton Integrated Research Ethics Board-5437-C.

### #4 Controlled dander aerosolization in a naturalistic exposure chamber

#### Laura Haya^1^, Rym Mehri^2^, Suzanne Kelly^1^, Bryan Santone^1^, Edgar Matida^2^, William H. Yang^1^

##### ^1^Red Maple Trials Inc., Ottawa, ON, Canada; ^2^Carleton University, Ottawa, ON, Canada

###### **Correspondence:** Laura Haya

*Allergy Asthma Clin Immunol* 2020, **16(Suppl 1):**#4

**Background:** A naturalistic exposure chamber housing two neutered male cats has been developed (Red Maple Trials, Ottawa) to test allergic responses of subjects during controlled exposures to Fel d 1. To improve upon traditional methods of dander aerosolization in which bedding is shaken resulting in transient levels of allergen, we have developed an automated delivery system with adjustable flow, using a modified robotic vacuum cleaner. Here, we present the range of Fel d 1 and particle concentrations produced by our system.

**Methods:** Circuitry was added to the vacuum, enabling full control of its suction power level to achieve a range of allergen levels. Controlled remotely, the vacuum passed throughout the chamber (floor area = 15.1 m^2^) for up to 2 h, aspirating dander that naturally collected on the floor and venting it through a custom exhaust tube. Air samples were obtained using portable air sampling pumps (Gillian 5000) at three locations across the chamber and for multiple time intervals. Fel d 1 deposited on glass fiber filters was quantified using ELISA. Numbers and sizes of dander particles were measured in real time using a time-of-flight particle size distribution analyser (PSD 3603, TSI Incorporated). Tests were performed for a range of suction power levels and for operation with and without brushes.

**Result**s: A fivefold increase in particle aerosolization was observed from the lowest to the highest power setting. A significant range of Fel d 1 concentrations was also measured. The vacuum’s brushes significantly increased the airborne particulate and allergen level.

**Conclusion:** A novel automated system to aerosolize animal dander has been developed with controls to generate a range of allergen concentrations. This provides a means of better controlling subject exposure to allergen for cat allergy studies, while maintaining a naturalistic environment.

### #5 Validation of a cat dander Naturalistic Exposure Chamber (NEC ™)

#### Suzanne Kelly^1^, Jacob Karsh^1^, Rym Mehri^2^, Bryan Santone^1^, Jenna Falbo^1^, William H. Yang^1^

##### ^1^Red Maple Trials Inc., Ottawa, ON, Canada; ^2^Department of Mechanical and Aerospace Engineering, Carleton University, Ottawa, ON, Canada

###### **Correspondence:** Suzanne Kelly

*Allergy Asthma Clin Immunol* 2020, **16(Suppl 1):**#5

**Background:** A naturalistic cat exposure chamber (NEC) with allergen concentrations similar to homes with cats can provide a relevant model to test allergy medications in a controlled environment with stable Fel d 1 levels. We report here on the technical and clinical validation of the NEC.

**Methods:** Allergen buildup was assessed over 2 years to assess stability of allergen levels. Walls and floor were swabbed using glass fiber filters (Millipore) for sampling, while airborne allergen was measured at 3 points using portable air sampling pumps (Gilian 5000) at 4 L/min with glass fiber filters. Fel d 1 was quantified by ELISA (Indoor Biotechnologies). Six subjects (4 allergic and 2 non-allergic) underwent two 60-minn challenges 1 week apart during which cat bedding was shaken every 15 min. Nasal, ocular and respiratory symptoms were captured every 5 min using a 4-point severity scale and spirometry was performed every 15 min. The study was approved by Advarra Ethics Board, approval number Pro 00023880. NCT03414801.

**Results:** Two-year averages for Fel d 1 were: floor 20.2 ± 14.7 µg/m^3^, walls 2.3 ± 1.67 µg/m^3^. Variation was due to build up and cleaning. Airborne Fel d 1 was below the limit of detection without disturbance. During subject challenges, room Fel d 1 was 189.5 ± 183.4 ng/m^3^; personal samplers reported 63.4 ± 51.2 ng/m^3^. Rhinoconjunctivitis symptoms for the two challenges in the 4 cat-allergic subjects were similar (6.5 ± 1.7 vs. 6.25 ± 3.36). Nasal symptoms predominated (5.25 ± 2.1, Challenge 1; 4.0 ± 2.4, Challenge 2). Allergic subjects reported modest respiratory discomfort during the challenges although none experienced a fall in FEV1. The 2 non-cat allergic subjects experienced no nasal, ocular or respiratory symptoms.

**Conclusion:** With disturbance, the NEC maintains airborne Fel d 1 levels similar to homes and induces reproducible symptoms in allergic subjects while non-allergic subjects remain asymptomatic.

### #6 Prevalence and predictive factors of pollen food syndrome in a Canadian cohort of birch, grass and/or ragweed allergic individuals

#### Sanju Mishra^1,2^, Lisa Steacy^3^, Dan Adams^3^, Anne K. Ellis^2,3^

##### ^1^Department of Medicine, Division of Clinical Immunology & Allergy, Western University, London, ON, Canada; ^2^Department of Medicine, Division of Allergy & Immunology, Queen’s University, Kingston, ON, Canada; ^3^Allergy Research, Kingston Health Sciences Centre Kingston General Hospital Site, Division of Allergy & Immunology, Kingston, ON, Canada

###### **Correspondence:** Sanju Mishra

*Allergy Asthma Clin Immunol* 2020, **16(Suppl 1):**#6

**Background:** Pollen food syndrome (PFS) is an IgE-mediated food allergy that occurs in aeroallergen sensitized individuals, due to cross-reactivity of plant proteins with homologous proteins present in food. In North America, culprit aeroallergens include: birch, grass and ragweed. Few prognostic factors for PFS have been identified, and there is limited data on the epidemiology of this condition in Canada.

**Methods:** A retrospective chart review was completed of patients seen in a single Canadian allergy clinic in Kingston, ON, Canada. Adult patients with allergic rhinitis and sensitization to birch, grass and/or ragweed pollen, based on positive skin prick testing, who were seen between 2009 and 2018 were included in our study. Patients were considered to have PFS based on physician diagnosis. Variables of interest such as multiple aeroallergen sensitization were assessed with a Pearson’s Chi-square test. The relationship between skin prick test size of aeroallergens of interest and the presence of PFS was assessed using a binomial logistic regression. The study was approved by Queen’s University Ethics Board.

**Results:** Of 400 patients identified with sensitization to an aeroallergen of interest, 82 (21%) had a concomitant physician diagnosis of PFS. PFS occurred significantly more frequently in those patients with multiple aeroallergen sensitization (p < 0.001) and birch and alder sensitization, compared to birch sensitization alone (p = 0.002). Larger skin prick test size to birch was associated with the presence of PFS (OR = 1).

**Conclusion:** Our data, similarly to previous studies, suggests that multiple aeroallergen sensitization is associated with PFS. Additionally, birch and alder co-sensitization were associated with higher rates of PFS than birch sensitization alone, and larger skin prick test size to birch was correlated with PFS. In our Canadian cohort of patients, the rate of PFS was 21%.

### #7 Dupilumab improved lung function despite exacerbation events in patients with uncontrolled, moderate-to-severe asthma during the LIBERTY ASTHMA QUEST study

#### Klaus F. Rabe^1,2^, Mario Castro^3^, Sally E. Wenzel^4^, Jonathan Corren^5^, Ian D. Pavord^6^, Constance Katelaris^7,8^, Yuji Tohda^9^, Megan S. Rice^10^, Paul Rowe^11^, Heribert W. Staudinger^11^, Nikhil Amin^12^, Bolanle Akinlade^12^, Neil M.H. Graham^12^, Ariel Teper^11^

##### ^1^LungenClinic Grosshansdorf (member of the German Center for Lung Research [DZL]), Airway Research Center North (ARCN), Grosshansdorf, Germany; ^2^Christian-Albrechts University (member of the German Center for Lung Research [DZL]), Airway Research Center North (ARCN), Kiel, Germany; ^3^Washington University School of Medicine, St. Louis, MO, USA; ^4^University of Pittsburgh Asthma Institute at the University of Pittsburgh Medical Center, Pittsburgh, PA, USA; ^5^David Geffen School of Medicine at UCLA, Los Angeles, CA, USA; ^6^University of Oxford, Oxford, UK; ^7^Campbelltown Hospital, Campbelltown, NSW, Australia; ^8^Western Sydney University, Sydney, NSW, Australia; ^9^Faculty of Medicine, Kindai University, Osakasayama, Japan; ^10^Sanofi, Cambridge, MA, USA; ^11^Sanofi, Bridgewater, NJ, USA 12Regeneron Pharmaceuticals, Inc., Tarrytown, NY, USA; ^12^Regeneron Pharmaceuticals, Inc., Tarrytown, NY, USA

###### **Correspondence:** Klaus F. Rabe

*Allergy Asthma Clin Immunol* 2020, **16(Suppl 1):**#7

**Background:** Dupilumab, a fully human monoclonal antibody, blocks the shared receptor component for IL-4/IL-13, key drivers of type 2 inflammation in multiple diseases. In the USA, dupilumab is approved for patients aged ≥ 12 years with moderate to severe eosinophilic or oral corticosteroid-dependent asthma and for patients aged ≥ 12 years with inadequately controlled, moderate-to-severe atopic dermatitis. In the phase 3 QUEST study (NCT02414854), dupilumab 200 mg/300 mg every 2 weeks, vs. matched placebo, reduced severe exacerbations and improved pre bronchodilator FEV1 and quality-of-life in uncontrolled, moderate-to-severe asthma patients. This post hoc analysis assessed dupilumab’s effect on lung function by number of exacerbations during the 52-week study period.

**Methods:** LS mean change from baseline in pre- and post-bronchodilator FEV1 at Week 52, adjusted for baseline FEV1, was assessed in patients experiencing 0, 1, 2, ≥ 3 exacerbations.

**Results:** More dupilumab- than placebo-treated patients had 0 exacerbations during the study. At Week 52, dupilumab 200 mg/300 mg vs. matched placebo significantly improved pre-bronchodilator FEV1 by 0.20 L (95% CI: 0.12, 0.27)/0.11 L (0.03, 0.18) and post-bronchodilator FEV1 by 0.20 L (0.13, 0.26)/0.13 L (0.06, 0.20), respectively, in patients experiencing 0 exacerbations (all P < 0.01). At Week 52, LS mean difference in change from baseline post-bronchodilator FEV1 was 0.14 L (0.02, 0.26)/0.01 L (− 0.11, 0.14), 0.12 L (− 0.08, 0.32)/0.10 L (− 0.09, 0.30), and 0.12 L (− 0.14, 0.37)/0.13 L (− 0.09, 0.35) in patients experiencing 1, 2, and ≥ 3 exacerbations, for dupilumab 200 mg/300 mg vs. matched placebo, respectively. Similar trends in pre-bronchodilator FEV1 at Week 52 were observed in these subgroups. The most frequent AE in dupilumab-treated patients vs. matched placebo was injection-site reaction (15%/18% vs. 5%/10%).

**Conclusion:** Most dupilumab-treated patients had 0 exacerbations during the study. In placebo-treated patients, lung function worsened with increasing numbers of exacerbations. Dupilumab-treated patients had improved post-bronchodilator FEV1, though the magnitude of improvement diminished with increasing exacerbations. These data highlight the relationship between exacerbations and lung function preservation in moderate-to-severe asthma.

### #8 The SQ tree SLIT-tablet reduces rhinoconjunctivitis symptoms and medication use during the tree pollen season (hazel, alder and birch pollen seasons): results from a large multi-center phase III trial

#### Tilo Biedermann^1^, Piotr Kuna^2^, Petr Panzner^3^, Dorthe Thrane^4^, Helle Frobøse Sørensen^4^, Karen Rance^5^, Erkka Valovirta^6,7^

##### ^1^Department of Dermatology and Allergology, Technical University of Munich, Munich, Germany; ^2^Division of Internal Medicine, Asthma and Allergy, Barlicki University Hospital, Medical University of Lodz, Lodz, Poland; ^3^Department of Immunology and Allergology, Faculty of Medicine in Pilsen, Charles University, Prague, Czech Republic; ^4^ALK, Hørsholm, Denmark; ^5^ALK, Bedminster, NJ, USA; ^6^Department of Pulmonary Diseases and Clinical Allergology, University of Turku, Turku, Finland; ^7^Terveystalo Allergy Clinic, Turku, Finland

###### **Correspondence:** Karen Rance

*Allergy Asthma Clin Immunol* 2020, **16(Suppl 1):**#8

**Background:** The SQ tree SLIT-tablet (ALK, Denmark) is being developed for treatment of moderate-to-severe allergic rhinitis and/or conjunctivitis induced by pollen from the birch homologous group. We report the results of a phase III trial.

**Methods:** TT-04 (EudraCT 2015-004821-15) was a DBPC trial. IRB approval was obtained from 57 sites. 634 subjects were randomized 1:1 to the SQ tree SLIT-tablet (12 DU dose) or placebo. Subjects received ≥ 16 weeks of treatment before the start of the hazel, alder and birch pollen seasons; i.e., tree pollen season (TPS) in 2017. Daily symptom score (DSS), daily medication score (DMS) and the sum of these; i.e., total combined score (TCS) were assessed during the birch pollen season (BPS) and TPS. The primary endpoint was average TCS during the BPS.

**Results:** Treatment effects on the average TCS, DSS and DMS in the BPS and TPS were all statistically significantly greater for the SQ tree SLIT-tablet versus placebo. Average TCS during the BPS showed an estimated absolute difference of 3.02 corresponding to a reduction of 39.6% in favor of the SQ tree SLIT-tablet relative to placebo (p < .0001). For the average TCS during the TPS the estimated absolute difference was 2.27 (36.5% relative to placebo; p < .0001). For the average DSS, the estimated absolute differences were 1.32 for the BPS (36.8% relative to placebo; p < .0001), and 0.99 for the TPS (32.7% relative to placebo; p < .0001). DSS and DMS contributed almost equally to the observed treatment effect in both the BPS and TPS.

**Conclusion:** A positive risk–benefit balance with a clinically relevant treatment effect of the SQ tree SLIT-tablet during the entire TPS was demonstrated.

**Results:** Treatment was well-tolerated. Local reactions were the most common treatment-related events; the majority were mild or moderate in severity. No deaths or anaphylactic reactions were reported with the SQ tree SLIT-tablet.

## Allied health

### #9 Impact of public healthcare provider counselling on early peanut introduction at home

#### Lianne Soller^1^, Jennifer Scarr^2^, Barbara Crocker^3^, Jennifer Schneidereit-Hsu^3^, Daniel Naiman^3^, Edmond S. Chan^1^, Radhika Bhagat^3^, Claire Heath^3^, Zahra Hussein^2^, Debby Rhodes^3^, Kyla J. Hildebrand^1^

##### ^1^Division of Allergy & Immunology, Department of Pediatrics, University of British Columbia, and BC Children’s Hospital, Vancouver, BC, Canada; ^2^Child Health BC, Vancouver, BC, Canada; ^3^Vancouver Coastal Health, Vancouver, BC, Canada

###### **Correspondence:** Lianne Soller

*Allergy Asthma Clin Immunol* 2020, **16(Suppl 1):**#9

**Rationale:** In 2017, NIAID guidelines recommending early peanut introduction to infants at high risk of peanut allergy were released. In Canada, some parents with concerns about severe allergic reactions to peanut seek the advice of nurse or dietitian public healthcare providers (PHPs). This pilot project sought to increase confidence of parents about introduction of non-choking peanut at home after meeting with PHPs who had received training through an allergist-facilitated education session. This is a novel knowledge translation collaboration of tertiary care providers (allergists) and PHPs (nurses and dietitians).

**Methods:** With input from PHPs to identify educational needs, an education session was developed and delivered to a group of PHPs by a pediatric allergist. A PHP (dietitian) then communicated this information to parents in an infant nutrition session. Pre and post surveys were distributed to parents attending nutrition sessions to assess parental confidence (1 = no confidence to 5 = very confident) in introducing non-choking peanut at home. A follow-up survey was conducted 3 months later.

**Results:** 65 parents completed the pre and post surveys at the drop-in session, and 43 (66%) completed the 3-month follow-up survey. Following the interaction with the dietitian, confidence among parents in introducing non-choking peanut at home increased from 3.61 (95%CI 3.34–3.87) to 4.24 (95%CI 4.05–4.42), for an increase of 0.60 (95%CI 0.41–0.79). Confidence in introducing non-choking peanut at home was 4.45 (95%CI 4.18–4.72) at the 3-month survey, a significant increase from the pre-survey (increase of 0.97, 95%CI 0.59–1.35) and the post-survey (increase of 0.34, 95%CI 0.03–0.65).

**Conclusions:** Parents of infants increased their confidence in introducing non-choking peanut at home following an interaction with a PHP trained through an allergist-facilitated education session. This increased confidence was sustained 3 months later, suggesting that targeted education of parents by PHPs may play a role in the prevention of peanut allergy.

### #90 Mast cell activation syndrome (MCAS): symptom prevalence and characteristic of patients with this presumed diagnosis

#### Alexander Shusterman^1^, Khaldon F. Abbas^1^, Masum Patel^1^, Gordon Sussman^1,2^

##### ^1^Gordon Sussman Clinical Research Inc., Toronto, ON, Canada; ^2^University of Toronto, TN, Ontario

###### **Correspondence:** Alexander Shusterman

*Allergy Asthma Clin Immunol* 2020, **16(Suppl 1):**#90

**Background:** Mast Cell Activation Syndrome (MCAS) is a disorder characterized by irregular mast cell degranulation [1]. It is a newly recognized disorder that is not yet fully understood. Patients with this disorder experience repeated anaphylactic type reactions without a clear cause. The aim of our study is to characterize and examine the frequency of symptoms suggestive of MCAS in a cohort of patients presenting with this condition.

**Methods:** Charts of 9 patients seen in an allergy clinic presenting with MCAS were isolated are reviewed. Demographics, medical history, clinical features, laboratory findings and symptoms were assessed and summarized. Symptoms were categorized based on their organ system involvement including gastrointestinal, cutaneous, respiratory, and cardiovascular systems.

**Results:** All patients were female with a mean age of 38 years (range 7–51). Common medical co-morbidities include food/environmental allergies (5/9), POTS (3/9), hypothyroidism (3/9), Ehlers-Danlos Syndrome (2/9), asthma (2/9), Irritable Bowel Syndrome (2/9), and anxiety (2/9). All 9 patients reported gastrointestinal symptoms, the most prevalent of these symptoms being vomiting, diarrhea and abdominal cramping. 8 patients reported cutaneous symptoms including hives and swelling. 3 patients reported respiratory symptoms including shortness of breath and wheezing. 3 patients reported cardiovascular symptoms including tachycardia, hypotension, and loss of consciousness. All patients reported fatigue. Serum tryptase was elevated in only one patient.

**Conclusion:** MCAS is a predominantly female disorder with symptoms encompassing multiple organ systems. This disorder presents mostly with gut and skin complaints. Respiratory and cardiovascular symptoms, although not as prevalent as gut and skin symptoms, is common in patients with this condition. Elevated tryptase levels can be present in only a small portion of patients with this presumed diagnosis, indicating low reliability of serum tryptase measurement as a diagnostic test. A study with a larger cohort of patients is warranted to better evaluate the prevalence of symptoms in patients with MCAS.

#### Reference

1. Akin C, Valent P, Metcalfe D. Mast cell activation syndrome: Proposed diagnostic criteria. J Allergy Clin Immunol. 2010;126:1099–104.

## Case report

### #10: An uncommon case of insulin allergy

#### Babak Aberumand^1^, Samira Jeimy^2^

##### ^1^Department of Medicine, Queen’s University, Kingston, ON, Canada; ^2^Division of Allergy & Immunology, Department of Medicine, Western University, London, ON, Canada

###### **Correspondence:** Babak Aberumand

*Allergy Asthma Clin Immunol* 2020, **16(Suppl 1):**#10

**Background:** Insulin hypersensitivity is rare, but challenging for individuals with diabetes. The prevalence of insulin allergy has decreased since the introduction of human recombinant insulin preparations. Hypersensitivity reactions range from injection site erythema and swelling to anaphylaxis. While some reactions are to excipients (zinc, protamine, metacresol), the majority are to insulin. We present a case of type 1 hypersensitivity to various preparations of insulin in a patient with insulin-dependent type 2 diabetes mellitus.

**Case presentation:** A 61-year-old woman with a 30-year history of insulin-dependence was referred for evaluation of reaction to insulin. She had two episodes over 5-months; both required Emergency Department visits and epinephrine administration. The first episode entailed pruritus of the extremities and nausea, immediately after injecting NovoRapid insulin. The second event entailed angioedema, respiratory distress and hypotension, immediately after injecting Detemir and NovoRapid, and taking metformin. There were no cofactors such as exercise, infectious illness, or NSAIDs. Skin testing was performed with metformin, Lantus, Humalog, NovoRapid, Glulisine, Insulin Regular, NPH, Detemir and the excipient protamine, as per published testing concentrations. metacresol was not done as its use was restricted by the hospital pharmacy. Insulin preparations with and without metacresol were included in testing. A clinic staff served as negative control. The patent had negative testing with protamine, but sensitization to all insulin preparations. Metformin skin test and challenge along with latex IgE were negative. She declined a challenge with insulin and was recommended insulin desensitization followed by use of an insulin pump.

**Conclusions:** Our case highlights the importance of diagnosing insulin allergy through a detailed history and focused testing. Therapeutic strategies include avoidance and the use of oral hypoglycemics, alternate insulin preparation, or desensitization followed by insulin pump. In some situations, omalizumab and desensitization therapy have been used successfully.

*Consents were obtained from the patient(s) for all case reports.

Consent has been received

### #11 Anaphylaxis to edible cricket powder

#### Leila A. Alenazy^1^, Rozita Borici-Mazi^2^

##### ^1^Department of Medicine, Queen’s University, Kingston, ON, Canada; ^2^Division of Allergy & Immunology, Department of Medicine, Queen’s University, Kingston, ON, Canada

###### **Correspondence:** Leila A. Alenazy

*Allergy Asthma Clin Immunol* 2020, **16(Suppl 1):**#11

**Background:** Cricket powder form a new high protein source with growing popularity among North American consumers. Previous studies have reported anaphylaxis following the consumption of insects and cross reactivity between shellfish and insects such as crickets and mealworm has been identified [1–3]. Here in, we describe a case that demonstrates a clinically relevant co-sensitisation to crickets powder in a patient with shellfish allergy.

**Case presentation:** A 22 years old male with Crhon’s disease was referred to the Allergy Clinic at Queen’s University for evaluation of anaphylaxis caused by ingestion of cricket powder. He presented to the Emergency Department with epigastric pain, generalized pruritus, hives with erythema and swelling of his hands and forearms. His symptoms developed 40 min after ingestion of cookies made with cricket powder. He was treated with intravenous antihistamines. He had never consumed cricket powder or other edible insects before. He had a history of allergic rhinitis to environmental allergens. He was told he had a shellfish allergy, but did not recall prior reaction. He denied history of anaphylaxis, or childhood asthma. In clinic, he underwent skin prick testing (SPT) to environmental and shellfish allergens. He reacted to dust mites, cockroaches, grass, ragweed, and birch pollen. SPT was positive for shrimp, crab and lobster, but negative to molluscs. SPT with diluted saline slurry of cricket powder produced a strongly positive reaction, with appropriate controls and was negative when applied on a healthy volunteer. He was diagnosed with IgE mediated cricket powder allergy. He was advised to avoid consumption of cricket powder and traces and carry an epinephrine autoinjector. He preferred to continue avoiding all shellfish.

**Conclusion:** Shellfish allergic/sensitive patients are at risk of developing an allergy to edible cricket. Therefore, these patients should be notified about this allergy risk and consider for evaluation before consumption of cricket.

**Statement of consent:** Written informed consent was obtained from the patient.

#### **References**

1. Kamemura N, et al. Cross-allergenicity of crustacean and the edible insect Gryllus bimaculatus in patients with shrimp allergy. Mol Immunol 2019;106:127–34.

2. Broekman H, et al. Majority of shrimp-allergic patients are allergic to mealworm. J Allergy Clin Immunol 2016;137:1261–63.

3. Broekman H, et al. Is mealworm or shrimp allergy indicative for food allergy to insects? Mol Nutr Food Res 2017:61.

### #12 A non-pigmenting fixed drug eruption (NPFDE) to Cephalexin in a child

#### Zeinah AlHalees^1^, Moshe Ben-Shoshan^2^, Elena Netchiporouk^1^

##### ^1^Division of Dermatology, McGill University, Montreal, QC, Canada; ^2^Division of Allergy & Clinical Immunology, McGill University, Montreal, QC, Canada

###### **Correspondence:** Zeinah AlHalees

*Allergy Asthma Clin Immunol* 2020, **16(Suppl 1):**#12

**Background:** Fixed drug eruption is a cutaneous delayed hypersensitivity reaction mediated by cytotoxic and memory T cells, retained in the epidermis and reactivated upon re-exposure to an offending drug. NPFDE is a relatively rare variant, with only a few cases reported in adults, and even fewer in children. We describe a case of NPFDE in a child presenting with a right knee lesion post Cephalexin oral provocation testing.

**Case presentation:** A 6-year-old girl was referred to allergy clinic for assessment of Cephalexin allergy, after developing a red lesion localized to the right knee 5 days post Cephalexin exposure. She subsequently received Amoxicillin with no adverse reactions. Eight hours post Cephalexin oral provocation testing, she again developed a right knee lesion. Cutaneous examination revealed a well-demarcated, erythematous, edematous plaque over the right knee, which resolved without residual hyperpigmentation 3 days later. The provisional diagnosis of NPFDE was further supported on skin histopathology showing superficial and deep perivascular infiltrates of lymphocytes and eosinophils, with dermal edema and rare melanophages.

**Conclusion:** NPFDE is associated with multiple medications, but has not been previously reported with Cephalexin. The recurrence of lesions at the same site, post challenge, and the absence of pigmentation thereafter support the diagnosis of NPFDE to Cephalexin in our patient. Oral provocation testing seems to be a safe and accurate diagnostic strategy, though larger scale studies are required to confirm this. The risk of Cephalexin cross reactivity with other beta-lactam antibiotics remains unclear. Our patient tolerated Amoxicillin but not Cephalexin, suggesting that cross reactivity might be independent from the R1-group side chains.

**Statement of consent:** Written informed consent was obtained.

### #13 Severe perioperative anaphylaxis caused by Patent Blue Dye injection for sentinel lymph node mapping: a report of two cases

#### Walaa Almasri^1^, Peter Maliha^2^, Gilbert Matte^1^, Michael Fein^1^

##### ^1^Department of Clinical Immunology and Allergy, McGill University, Montreal, QC, H3G 1A4, Canada; ^2^Department of Nuclear Medicine, McGill University, Montreal, QC, H3G 1A4, Canada

###### **Correspondence:** Walaa Almasri

*Allergy Asthma Clin Immunol* 2020, **16(Suppl 1):**#13

**Background:** Patent Blue (PB) is an aniline dye commonly used for intraoperative lymph node mapping for patients with malignant cancers. Hypersensitivity reactions are infrequent, underrecognized and can be severe with anaphylactic shock and rarely cardiac or respiratory arrest.

**Case 1:** A 61-year-old male patient with no atopic history diagnosed with melanoma was admitted for sentinel lymph node mapping and excision of a right flank melanoma. During induction, the patient received Ancef, Fentanyl, Propofol, Succinylcholine, topical Chlorhexidine 2%, and Toradol, followed by Patent Blue and technetium-99 Sulfur-Colloid. Within 15 min of induction, he developed profound hypotension followed by cardiac arrest. Cardiopulmonary resuscitation was initiated as well as epinephrine infusion which results in return of spontaneous circulation. Skin testing for all possible culprits was negative except for PB (25 mg/mL) which was positive for both 1:10 and full-strength dilutions. He subsequently underwent successful surgery without the use of Patent Blue.

**Case 2:** A 32-year-old female diagnosed with cervical squamous cell carcinoma was admitted for radical hysterectomy and sentinel lymph node dissection. At induction, she received Ancef, Fentanyl, Propofol, Succinylcholine, topical Chlorhexidine 2%, followed by injection of 99-Technecium- Sulfur-Colloid and Patent Blue. Shortly after the injection of Sulfur Colloid and PB, the patient developed urticaria and angioedema and became severely hypotensive. She required resuscitation with vasopressors and the surgery was aborted. Skin testing to PB (25 mg/mL) was positive for both 1:10 and full-strength dilutions. Repeat surgery was performed successfully without the use of Patent Blue.

**Conclusion:** Patent Blue is a rare but important cause of severe perioperative anaphylaxis. It is important to consider all perioperative agents when evaluating patients including dyes, radiopharmaceuticals, and antiseptics in addition to medications.

**Statement of consent:** Written informed consent was obtained.

### #14 Castleman disease

#### Ashwag Alsaidalan, Reza Alizadehfar

##### Department Pediatrics, Division of Allergy & Immunology, McGill University, Montreal, QC, Canada

###### **Correspondence:** Ashwag Alsaidalani

*Allergy Asthma Clin Immunol* 2020, **16(Suppl 1)**:**#**14

**Background:** Castleman disease (CD) is a rare heterogeneous group of lymphoproliferative disorder of unknown etiology most often presenting in adulthood with nonspecific symptoms. It can be localized to one lymph node (monocentric) or generalized with constitutional symptoms (multicentric). Its definitive diagnosis requires specific histopathological features.

**Case presentation:** A 16-year-old male was referred by hematology to our immunology service for persistent fever, weight loss, mild splenomegaly and generalized fatigue for 3 months. He had a mild anemia and a thrombocytopenia. He underwent initially a PET scan and two bone marrow aspirations ruling out a malignant etiology. Genetic testing for a panel of genes involved in auto-inflammatory syndromes did not reveal any relevant mutation. His evaluation and extensive investigation by infectious disease and rheumatology services did not reveal a cause to his persistent symptoms. The Patient was eventually re-admitted again after a year. Upon further investigation, the PET scan showed a positive signaling in multiple lymph nodes (LN). A biopsy of the supraclavicular LN was compatible with a definitive diagnosis of CD. A regimen of corticosteroids and anti-IL6 monoclonal antibody therapy led to significant improvement of his symptoms.

**Conclusion:** Castleman disease is a rare disease in the pediatric population. Given the nonspecific manifestations, a high index of suspicion and repeated investigations are required for its proper diagnosis and management.

**Statement of consent:** Written informed consent was obtained.

### #15 Anaphylaxis caused by trace exposure to angiotensin II blocker in a healthcare worker: a case report

#### Angeliki Barlas^1^, Raymond Mak^2^

##### ^1^Clinical Immunology and Allergy, University of British Columbia, Vancouver, BC, Canada; ^2^Clinical Immunology and Allergy, University of British Columbia, Vancouver, BC, Canada

###### **Correspondence:** Angeliki Barlas

*Allergy Asthma Clin Immunol* 2020, **16(Suppl 1):**#15

**Background:** Angiotensin-converting enzyme inhibitor (ACEI)-related angioedema is well described in the medical literature. However, angiotensin II receptor blocker (ARB)-related angioedema and anaphylaxis is not as well elucidated due to its lower incidence. There are limited reports of severe ARB-related adverse drug reactions in the literature and its definitive mechanism remains unknown. In healthcare workers, one of the possible aetiologies of anaphylaxis includes occupational exposures to medications.

**Case presentation:** A 57-year-old female was referred to our clinic for evaluation of unexplained anaphylaxis. On detailed history, the patient, who works as a Care Aide, handled Irbesartan and Atenolol at a client’s house earlier that day and then subsequently rubbed her eyes. Within 10 min of contact she developed lip swelling, flushing and rashes on the face. She presented to the emergency department where she was noted to be stridorous. She was treated with epinephrine, antihistamines and steroids but required intubation for respiratory distress. Later in the clinic, a graded oral challenge to Irbesartan resulted in substantial hypotension, dizziness, nausea and visible lip angioedema. She required two doses of epinephrine in the office which led to resolution of her symptoms. Subsequently, she tolerated a graded Atenolol challenge. She was diagnosed with an ARB allergy. Suggestions were provided for a modified workplace to ensure ongoing safety.

**Conclusion:** Occupational allergy in health care workers can result in anaphylaxis. In healthcare workers, medications as a cause for anaphylaxis is often overlooked, especially when the causative agent is seemingly benign. Although an ARB allergy is rare, unusual drug allergies may be the cause of “unexplained” anaphylaxis even with trace exposures. When evaluating such a patient, a detailed history of medication exposure in the workplace and pursuing drug provocation testing are necessary for accurate diagnosis.

Consent to publish was obtained from the patient involved in this study.

### #16 Failure of immunotherapy as treatment of Post-orgasmic Illness Syndrome (POIS)

#### Ana-Maria Bosonea, Amin S. Kanani

##### Division of Allergy and Immunology, Department of Medicine, University of British Columbia, Vancouver, BC, Canada

###### **Correspondence:** Ana-Maria Bosonea

*Allergy Asthma Clin Immunol* 2020, **16(Suppl 1):**#16

**Background:** POIS is a rare disease first defined in 2002. Since then, 50 cases have been described in literature, but it remains largely under-recognized in the medical community. Although the pathophysiology is unknown, various treatments have been tried on a small scale including 3 published case reports of immunotherapy successfully decreasing POIS symptoms. We present a case of unsuccessful immunotherapy in a patient with positive autologous seminal fluid skin testing.

**Case Presentation:** The patient is a 23-year-old male, meeting criteria for POIS with headaches, fatigue, generalized weakness, myalgias, arthralgias, sore throat and concentration problems within 1-h post-ejaculations lasting 1 week, occurring since puberty. On intradermal testing he had a positive reaction to seminal plasma 1:10 dilution (12 mm) and negative reaction to sperm. An immunotherapy preparation was made to the seminal plasma at a starting concentration of 1:100,000 and a maintenance concentration of 1:10 seminal fluid. The patient was treated with immunotherapy to autologous seminal fluid for a total of 4 years. Although after 3 years, the skin reaction to the 1:10 intradermal seminal fluid decreased from 12 to 3 mm, the symptoms were only minimally improved. Other treatments trialed include antihistamines, NSAIDs, and prednisone all without significant improvement.

**Conclusions:** Although there are previous case reports documenting the success of immunotherapy as treatment for POIS, this is the first publication reporting its’ unsuccessful use in a patient with POIS and positive skin testing, thus highlighting the gap in knowledge of the pathophysiology of this rare disease.

**Statement of consent:** Written informed consent was obtained.

### #17 Anaphylaxis to cow’s milk after pregnancy

#### Robyn Cochrane Fones^1^, Elissa M Abrams^1,2,3^

##### ^1^Meadowood Medical Centre, Winnipeg, MB, Canada; ^2^Department of Pediatrics, Section of Allergy and Clinical Immunology, University of Manitoba; ^3^Department of Pediatrics, Division of Allergy and Immunology, University of British Columbia, Canada

###### **Correspondence:** Robyn Cochrane Fones

*Allergy Asthma Clin Immunol* 2020, **16(Suppl 1):**#17

**Background:** IgE-mediated cow’s milk allergy is a common food allergy in childhood but is rare in adulthood. We report a case of a woman developing IgE-mediated cow’s milk allergy after the birth of her child. While there are rare case series noting adult-onset of cow’s milk allergy, this is the first reported in North America in the literature of this food allergy developing after pregnancy [1, 2].

**Case presentation:** We present a 43-year old female who tolerated cow’s milk products on a regular basis with no reaction prior to the birth of her child. After the birth of her child she noted that each ingestion of milk products would result in cutaneous symptoms (urticaria,) oral pruritus and many ingestions resulted in shortness of breath/cough. Total avoidance of milk was initiated following a severe reaction after ingesting a smoothie containing cow’s milk. This reaction resulted in requiring 2 doses of epinephrine for symptoms of urticaria, shortness of breath, cough, light-headedness and an episode of emesis. Epicutaneous testing was performed in clinic and highly positive(8 mm) to commercial cow’s milk extract (histamine control 6 mm, saline control negative)

**Conclusion:** This case is the first to our knowledge to document emergence of IgE-mediated cow’s milk allergy after pregnancy in North America. This case demonstrates that new food allergies after pregnancy should be considered and reiterates that adult cow’s milk allergy, as noted in previous case series, can be phenotypically severe.

**Statement of consent:** Written informed consent was obtained.

#### **References**

1. Lam H-Y, van Hoffen E, Michelsen A, Guikers K, van der Tas CHW, Bruijnzeel-Koomen CAFM, et al. Cow’s milk allergy in adults is rare but severe: both casein and whey proteins involved. Clin Exp Allergy. 2008;38:995–1002.

2. Stoger P, Wuthrich B. Type 1 allergy to cow milk proteins in adults. A retrospective study of 34 adult milk- and cheese-allergic patients. Int Arch Allergy Immunol. 1993;102:399–407.

### #18 A compound heterozygous Factor I mutation family and invasive pneumococcal infection

#### Hannah Laure Elfassy, Marylin Desjardins, Bruce Mazer

##### Immunology-allergy Department, Children’s Hospital of Montreal, McGill University, Montreal, Canada

###### **Correspondence:** Hannah Laure Elfassy

*Allergy Asthma Clin Immunol* 2020, **16(Suppl 1):**#18

**Background:** The infectious susceptibility to *Streptococcus Pneumoniae* remains a typical presentation of defects in the classical complement pathway. The regulatory proteins of the alternative complement pathway play a major role in the homeostasis of the latter. An abnormality of Factor I protein classically results in complement-mediated thrombotic microangiopathy, C3-glomerulonephritis or age-related macular degeneration. This unique case series reports a compound heterozygous pathogenic mutations of factor I in relation to pneumococcal invasive infections.

**Case presentation:** Two brothers of 3 and 7 years-old, coming from an unremarkable non-consanguine family, were evaluated in immunology in the context of pneumococcal pulmonary sepsis and repetitive acute otitis. Functional complement tests have demonstrated a complete inactivation of the alternative pathway (AH50 0%) and a major decreased activity in the classical (CH50 11–15%) and lectin pathway (MBL 0–46%). Serum sC5b9 is normal and Factor B as well as C2 to C9 were quantitatively decreased. These siblings carry 2 heterozygous mutations of CFIc.1429 + 1G>C and CFIc.485G>A(Gly162Asp) for which each parent is a healthy carrier. The in silico studies revealed major changes in the functioning of CFI, namely a major amputation of the serine protease domain as well as in the C5 domain. The qualitative measurement also confirms a significant decrease in factor I serum.

**Conclusions:** This rare family case proves the importance of CFI in the defense of the host. Indeed, this mutation generates a continuous and unregulated activation of the alternative complement pathway leading to depletion of main complement’s protein and encapsulated germs susceptibility. Depending on the factor I mutation, disparate clinical presentation could occur whether infectious, nephrological or microangiopathic. Identifying these patients early in life and providing them vaccination and antibioprophylaxis is important.

**Statement of consent:** Written informed consent was obtained.

### #19 It’s not just rice! Food protein-induced enterocolitis to grains: single vs multiple and the benefits of structured medical challenges

#### Kristiina Frechette^1,2^, Tarin T. Moni^1,2^, Vince Wu^2^, Sydney A. Scheffler^2^, Wardha Wardha^2^,Jason A. Ohayon^1,2^

##### ^1^McMaster University, Hamilton, ON, Canada; ^2^Hamilton Allergy, Hamilton, ON, Canada

###### **Correspondence:** Kristiina Frechette

*Allergy Asthma Clin Immunol* 2020, **16(Suppl 1):**#19

**Background:** Food Protein-Induced Enterocolitis Syndrome (FPIES) is a non-IgE-mediated food allergy that occurs in infancy during solid food introduction. FPIES manifests as delayed vomiting, irritability, ± diarrhea. Severe dehydration and shock may follow. Oral food challenges (OC) are needed to confirm FPIES triggers with IV fluids, ondansetron and adequate observation. Guidance for other grain introduction in single FPIES infants remains unknown.

**Case presentation:** A non-atopic 6 month-old female presented to a SW Ontario ER with recurrent emesis, cyanosis, and peripheral hypotonia 1–2 h after ingestion of rice cereal. Blood work revealed an elevated total WBC of 28.9 × 10^9^/L. Intravenous fluids and ondansetron were needed to recover the infant. The child was diagnosed with FPIES to rice based on presentation. Subsequent in-patient admissions were arranged to help assess tolerance to other grains, using published protocols (0.6 g protein/kg exposure) (1). Physician-supervised OC was performed to wheat and oats, on separate admissions. Neither of these grains were tolerated with profuse emesis occurring 2 h post-ingestion that required intravenous fluid rehydration and ondansetron for recovery. Blood work revealed elevated platelets, but not total WBC from in hospital challenge.

**Conclusions:** Multiple grain FPIES is rare; most FPIES to grains are single. This case highlights the benefits of structured in-hospital OC that confirmed multiple FPIES. In-hospital challenge was safe, in light of severe initial FPIES presentation to rice. Severe elevated WBC may be a marker of more complicated FPIES presentation.

**Statement of consent:** Written informed consent for publication of their clinical details and/or images was obtained from the parent of the patient. A copy of the consent form is available for review by the Editor of this journal.

#### **Reference**

1. Nowak-Węgrzyn A, et al. International consensus guidelines for the diagnosis and management of food protein–induced enterocolitis syndrome. JACI. 2017;139(4):1111–1126.e4.

### #20 An atypical presentation of ceftriaxone and vancomycin hypersensitivity reaction: a case report

#### Kelsey Hinther^1^, Chrystyna Kalicinsky^2^

##### ^1^Department of Internal Medicine, University of Manitoba, Winnipeg, MB, Canada; ^2^Department of Allergy and Clinical Immunology, University of Manitoba, Winnipeg, MB, Canada

###### **Correspondence:** Kelsey Hinther

*Allergy Asthma Clin Immunol* 2020, **16(Suppl 1):**#20

**Background:** Cephalosporins and vancomycin are increasingly used together to treat infections. With increase use, more adverse drug reactions have been reported. Currently, there is no clear evidence for the potentiation of rash when the two drugs are used together. Our case demonstrates this possibility.

**Case presentation:** A 63-year-old male, no history of drug allergy, developed a diffuse, pruritic maculopapular rash 5 days after initiation of ceftriaxone and vancomycin for severe lower leg cellulitis. Rash resolved within 4 days of discontinuing ceftriaxone and vancomycin. One week later, delayed intradermal testing (IDT) to ceftriaxone and vancomycin was negative, however testing precipitated recurrence of rash. Testing each antibiotic individually did not result in rash.

**Conclusions:** Further studies are needed to define ceftriaxone and vancomycin IDT sensitivity. Based on this case, we postulate that there is possible potentiation of rash when ceftriaxone and vancomycin are used together.

**Ethics approval:** The University of Manitoba ethics requirements for Case Report state that consent is required, and submission for ethics review is to be done before publication. Therefore submission for ethics review will be done if abstract is accepted for presentation.

### #21 Positive to negative basophil histamine release assay conversion in a patient with chronic spontaneous urticaria

#### Uliana Kovaltchouk^1^, Chrystyna Kalicinsky^2^

##### ^1^Internal medicine, University of Manitoba, Winnipeg, MB, Canada; ^2^Department of Allergy and Immunology, Internal medicine, University of Manitoba, Winnipeg, Canada

###### **Correspondence:** Uliana Kovaltchouk

*Allergy Asthma Clin Immunol* 2020, **16(Suppl 1):**#21

**Background:** The use of the basophil histamine release assay has expanded its use from being a diagnostic tool for the autoimmune subtype of chronic spontaneous urticaria, to depicting disease duration and severity, depending on a positive or negative assay result.

**Case presentation:** We describe a patient, diagnosed with the autoimmune subtype of chronic spontaneous urticaria with a positive basophil histamine release assay, who was treated with anti-histamine therapy until disease remission. Conversion of the assay to negative correlating with disease remission was observed. This is the first description, in current literature, that a positive to negative conversion has been observed.

**Conclusion:** This case suggests further research into the utility of the basophil histamine release assay as a biomarker for disease remission in the subset of patients with a positive assay at the time of diagnosis should be considered.

Written informed consent for publication of clinical details was obtained from the patient. A copy of the consent is available for review by the Editor of this journal.

### #22 Drug reaction with eosinophilia and systemic symptoms (DRESS) induced by amoxicillin-clavulanic acid. A report of two pediatric cases

#### Hasandeep Kular^1^, Bruce Carleton^2,3,4^, Michael Rieder^5^, Raymond Mak^1^, Tiffany Wong^4,6^

##### ^1^Division of Allergy and Immunology, Department of Medicine, University of British Columbia, Vancouver, BC, Canada; ^2^Department of Pediatrics, University of British Columbia, Vancouver, BC, Canada; ^3^Pharmaceutical Outcomes Programme, BC Children’s Hospital, Vancouver, BC, Canada; ^4^BC Children’s Hospital Research Institute, Vancouver, BC, Canada; ^5^Drug Safety Laboratory, Department of Paediatrics, Physiology & Pharmacology and Medicine, Western University, London, ON, Canada; ^6^Division of Allergy and Immunology, Department of Pediatrics, University of British Columbia, Vancouver, BC, Canada

###### **Correspondence:** Hasandeep Kular

*Allergy Asthma Clin Immunol* 2020, **16(Suppl 1):**#22

**Background:** Drug reaction with eosinophilia and systemic symptoms (DRESS) is a rare delayed hypersensitivity reaction, typically with a long latency period. DRESS is uncommon but potentially life-threatening in children. The lymphocyte toxicity assay (LTA) is an investigational assay measuring cell viability upon incubation with the suspected drug. We describe two cases of pediatric DRESS syndrome caused by amoxicillin-clavulanic acid.

**Case presentation:** Case 1 involves an 11-month-old male prescribed amoxicillin-clavulanic acid for a respiratory infection. 14 days after antibiotic exposure, he presented with fevers, lethargy, a generalized erythematous maculopapular rash, and facial, hand, and scrotal edema. Laboratory investigations revealed reactive lymphocytes, peripheral eosinophilia (0.72 × 10^9^/L) and hepatitis. Testing for ANA, Hepatitis A and B, EBV, CMV, HHV6, Mycoplasma, Chlamydia, and blood cultures were negative. A RegiSCAR-Group Diagnosis Score of 6 confirmed definite DRESS. LTA testing revealed a concentration-dependant decline in viability after incubation with penicillin and penicillin metabolites. Parental samples also displayed concentration-dependent decreased cell viability. Case 2 involves an 11-year-old female with atopic dermatitis prescribed amoxicillin-clavulanic acid for a secondary bacterial skin infection. She developed fevers and decreased appetite 11 days after antibiotic exposure with progression to diffuse erythrodermic, maculopapular eruption, superficial desquamation, facial angioedema, peripheral eosinophilia (6.8 × 10^9^/L), hepatitis, and lymphadenopathy. Her RegiSCAR-Group Diagnosis Score was 5, confirming probable DRESS. LTA revealed a substantial decline in viability when exposed to penicillin. Both cases were treated with systemic steroids with normalization of labwork and improvement of symptoms.

**Conclusions:** Amoxicillin-clavulanic acid is a commonly used antibiotic which should be recognized as a potential cause of DRESS in pediatric patients. The LTA can be a helpful tool in DRESS cases and may have potential implications for family members. Furthermore, these cases add to current literature supporting that there is a shorter latent period in DRESS induced by antibiotics in children.

**Statement of consent:** Written informed consent for publication was obtained from the parents of the patients.

### #23 Angiotensin-converting enzyme inhibitor-induced angioedema with vocal cord dysfunction, a case of a combination of diagnoses masquerading as hereditary angioedema with normal C1 inhibitor

#### Shun Chi Ryan Lo^1^, Raymond Mak^2^, Amin Kanani^3^

##### ^1^Department of Medicine, University of British Columbia, Vancouver, BC, Canada; ^2^Division of Allergy and Immunology, Department of Pediatrics, University of British Columbia, Vancouver, BC, Canada; ^3^Division of Allergy and Immunology, Department of Medicine, University of British Columbia, Vancouver, BC, Canada

###### **Correspondence:** Shun Chi Ryan Lo

*Allergy Asthma Clin Immunol* 2020, **16(Suppl 1):**#23

**Background:** Hereditary angioedema with normal C1 inhibitor (HAE-n-C1inh) is characterized by bradykinin-mediated swelling with normal level and function of C1 inhibitor (C1inh), family history of angioedema, no response to high-dose antihistamines, and exclusion of alternative causes. Genetic variations in factor XII, angiopoietin-1, and plasminogen have been described in some HAE-n-C1inh patients; however, diagnostic testing is limited. Angiotensin-converting enzyme inhibitors (ACEi) can cause bradykinin-mediated angioedema, lasting up to 2 months after ACEi discontinuation. Vocal cord dysfunction (VCD) causes respiratory symptoms including stridor, dyspnea, dysphonia and throat tightness secondary to abnormal vocal cord movements but is a benign condition.

**Case presentation:** A 58 years-old female with hypertension on ramipril presented with cough and stridor to a rural hospital. CT scan revealed epiglottic swelling. C1inh level and function were normal. The patient was intubated and treated with antihistamines and prednisone. Ramipril was discontinued with a provisional diagnosis of ACEi-induced angioedema. The patient continued to present with stridor for the next 7 months, for which she was intubated multiple times, and a tracheostomy performed. Further history revealed possible angioedema affecting several generations. A clinical diagnosis of HAE-n-C1inh was made. Testing for genetic variations in *SERPING1, F12,* and *PLG* was negative. Daily attacks continued despite prophylaxis with tranexamic acid, danazol, and C1 inhibitor. Acute attacks had minimal response to icatibant and C1 inhibitor. Upon transfer to tertiary care for reassessment, provocative maneuvers performed by otolaryngologists discovered VCD.

**Conclusions:** Common diagnoses in combination may mimic rare disorders. Initial angioedema may be explained by ACE inhibitors and subsequent episodes of stridor by VCD. Earlier reconsideration of the diagnosis may have prevented harm secondary to intubation and inappropriate treatment. The diagnosis of HAE-n-C1inh is difficult without reliable diagnostic testing; thus, caution in the exclusion of alternative diagnoses, and frequent re-evaluation is crucial for accurate patient management.

Written informed consent was obtained from the patient for publication of the case details.

### #24 ACE inhibitor induced angioedema treated with C1 inhibitor concentrate in the emergency department

#### Sarah Lohrenz^1^, Ibraheem Othman^2^

##### ^1^College of Medicine, University of Saskatchewan, Regina, SK, Canada; ^2^Alain Blair Cancer Centre, College of Medicine, University of Saskatchewan, Regina, SK, Canada

###### **Correspondence:** Sarah Lohrenz

*Allergy Asthma Clin Immunol* 2020, **16(Suppl 1):**#24

**Background:** Angioedema induced by treatment with angiotensin converting enzyme (ACE) inhibitors accounts for one-third of all cases of angioedema treated in the emergency room [1]. ACE inhibitors block the effects of the enzyme ACE, altering the renin–angiotensin–aldosterone pathway and ultimately leading to decreased degradation of bradykinin. High levels of bradykinin stimulate vasodilation and increased vascular permeability, leading to angioedema, which rarely may progress to airway obstruction. C1 inhibitor concentrate is used to treat hereditary angioedema by inhibiting the formation of bradykinin. Few case reports have also demonstrated its efficacy in ACE inhibitor induced angioedema [2–4].

**Case presentation:** A 71-year old male presented to the emergency department with angioedema (without urticaria) of the face, lips and tongue following 4 years of ACE inhibitor therapy. He developed respiratory distress in the emergency department that was not responsive to epinephrine or antihistamines. C1 esterase and complement (C3/C4) levels later measured were normal. He received C1 inhibitor concentrate and responded with significant improvement in edema and resolution of respiratory distress within 1 h of administration.

**Conclusions:** There is limited documentation in the literature to guide treatment of acute ACE inhibitor induced angioedema in the emergency department. We report an additional case treated successfully with C1 inhibitor concentrate.

Written informed consent for publication of their clinical details was obtained from the patient. A copy of the consent form is available for review by the Editor of this journal.

#### **References**

1. Banerji A, Clark S, Blanda M, et al. Multicenter study of patients with angiotensin-converting enzyme inhibitor-induced angioedema who present to the emergency department. Ann Allergy Asthma Immunol. 2008;100:327–32.

2. Nielsen E, Gramstad S. Angioedema from angiotensin-converting enzyme (ACE) inhibitor treated with complement 1 (C1) inhibitor concentrate. Acta Anaesthesiologica Scandinavica 2005;50(1):120–2.


3. Gelée B, Michel P, Haas R, Boishardy F. Angiotensin-converting enzyme inhibitor-related angioedema: emergency treatment with complement C1 inhibitor concentrate. Rev Med Interne 2008;29:516.

4. Rasmussen ER, Bygum A. ACE-inhibitor induced angio-oedema treated with complement C1-inhibitor concentrate. BMJ Case Rep. 2013;2013.

### #25 Is it allergy? Photodermatitis mimicking the effects of allergic contact dermatitis

#### Tarin Moni^1,2^, Kristiina Frechette^1,2^, Vince Wu^2^, Wardha Wardha^2^, Sydney A. Scheffler^2^, Jason Ohayon^1,2^

##### ^1^McMaster University, Hamilton, ON, Canada; ^2^Hamilton Allergy, Hamilton, ON, Canada

###### **Correspondence:** Tarin Moni

*Allergy Asthma Clin Immunol* 2020, **16(Suppl 1):**#25

**Background:** Adverse reactions to tattoos are frequently diagnosed as allergic contact dermatitis (ACD) to dyes. Patch testing is often inconclusive in diagnosing contact allergy in these patients.

**Case presentation:** Two patients were seen in the clinic for suspected ACD reactions to the red dye in their tattoos. A 65-year-old male with pre-existing tattoos containing red dye presented with pruritus and well-demarcated red, raised, and localized patches over the red dye tattoo (RDT) sites. Symptoms were treated with topical moisturizers and antihistamines. Assessment was carried out to ACD, dye and metal panels. Patch testing was only positive to copper. The patient was treated with Betaderm 0.1% cream. Five months later, he returned due to exacerbations of his dermatitis. Interestingly, only the RDT sites exposed to sunlight developed pruritus and rash, but unexposed RDT areas were unremarkable. The patient was diagnosed with photodermatitis of red dye, facilitated by the presence of copper, advised to cover affected areas while in the sun, and discharged home with oral prednisone. A 38-year-old male was seen for localized pruritic rashes over red/orange sites on his tattoo. Topical steroids were helpful, but symptoms returned upon sunlight exposure. The patient tested negative for ACD on all patch testing. Both patients denied adverse reactions to other colours in their respective tattoos.

**Conclusions:** It is hypothesized that composite pigments in RDT react strongly to UV-radiation, causing decomposition, fragmentation and formulation of reactive oxidative species, leading to compromised skin integrity, which is often misdiagnosed as ACD. Clinicians should be aware of this risk with RDT sites in sunlight for proper treatment and counsel.

**Consent to publish:** Written informed consent for publication of their clinical details and images was obtained from the patients. A copy of the consent form is available for review by the Editor of this journal.

### #26 Anaphylaxis to goat/sheep milk in a 4-year-old boy tolerant to cow milk

#### Pasquale Mulé^1^, Sofianne Gabrielli^1^, Julia Upton^2^, Abrams EM^3,4^, Moshe Ben-Shoshan^1^

##### ^1^Division of Allergy, Immunology, and Dermatology, Montreal Children’s Hospital, Montreal, QC, Canada; ^2^Division of Immunology and Allergy, The Hospital for Sick Children, Department of Paediatrics, University of Toronto, Toronto, ON, Canada; ^3^Department of Pediatrics, Section of Allergy and Clinical Immunology, University of Manitoba, Winnipeg, MB, Canada; ^4^Department of Pediatrics, Division of Allergy and Immunology, University of British Columbia, Vancouver, BC, Canada

###### **Correspondence:** Pasquale Mulé

*Allergy Asthma Clin Immunol* 2020, **16(Suppl 1):**#26

**Background:** Goat’s and sheep’s milk (GSM) allergies are rare in patients who display tolerance to cow’s milk (CM). Despite high cross reactivity of milk caseins between the three dairy products, immunological response may vary depending on the mammalian source. Patients with IgE antibodies that are unreactive to bovine β-casein are reported to be reactive to caprine β-casein, despite the significant sequence homology between the two proteins [1]. We present a case of anaphylaxis to sheep/goat cheese in a 4 year old boy who is tolerant to CM products, demonstrating a mammalian-specific dairy allergy.

**Case presentation:** A 4-year-old boy who is tolerant to CM presented with an allergic reaction within minutes after eating feta cheese (consisting of a mixture of GSM). The patient experienced drowsiness, abdominal cramps, angioedema, and was wheezing. He had known intermittent asthma, but no previous history of food allergies. The tryptase level measured 1 h post initial symptoms was 14.6 μg/L (norm: 0.0–13.5 μg/L). A tryptase repeated a month later was within normal limits (5.1 μg/L), confirming the diagnosis of anaphylaxis. A skin prick test performed 1 month after the reaction was highly positive for GSM (17 mm and 16 mm respectively), but negative for CM.

**Conclusions:** Care givers and patients with CM tolerance should be aware of the risk of allergy to milk caseins found in different mammals. Specific skin prick tests may help establish a GSM allergy, while allowing continuous consumption of CM. Finally, patients who successfully complete oral immunotherapy for CM allergy should be informed that their increased tolerance may be restricted to CM protein, and not to other mammals. This is the first case of a patient who is unreactive to CM, but reactive towards GSM to be reported in Canada.

Written informed consent for publication of the patient’s clinical details and clinical images was obtained from their parents. A copy of the consent form is available for review by the Editor of this journal.

#### **Reference**

Hazebrouck S, et al. Goat’s milk allergy without cow’s milk allergy: suppression of non-cross-reactive epitopes on caprine β-casein. Clin Exp Allergy. 2014;44:602–10.

### #27 Anaphylaxis to clindamycin following cutaneous exposure

#### Noémie Paradis^1^, Louis Marois^1^, François Graham^2^, Louis Paradis^1,2^, Philippe Bégin^1,2^, Anne Des Roches^1^

##### ^1^Pediatrics Department, Allergy Section, CHU Sainte-Justine, Montréal, QC, Canada; ^2^Medicine Department, Allergy Service, CHUM, Montreal, QC, Canada

###### **Correspondence:** Noémie Paradis

*Allergy Asthma Clin Immunol* 2020, **16(Suppl 1):**#27

**Background:** Allergen exposure through skin barrier is a known cause of sensitization in food allergy but has not been reported in drug allergy.

**Case presentation:** A 14-year-old boy was admitted to the intensive care unit for a suspected staphylococcus toxic shock syndrome. He presented multiples skin abscess, fever, headache, macular rash and low blood pressure at 102/49. He was treated with iv cefazolin and clindamycin. Five minutes after starting the clindamycin perfusion, he complained of throat tightening and dyspnea. Physical examination revealed angioedema, conjunctival hyperemia, generalized hives and wheezing. Saturation decreased to 88%. He was immediately treated with epinephrine, oxygen, diphenhydramine, salbutamol, hydrocortisone, and symptoms were rapidly controlled except for remaining low diastolic blood pressure. As he continued to be febrile and considering that clindamycin allergy is rare, moreover as he never received it before, we decided to perform a drug challenge. After perfusion of 380 mg, he presented throat tightness, hand pruritus, dyspnea, wheezing, and desaturation to 84%. Symptoms were rapidly controlled with epinephrine. At follow-up, prick and intradermal skin tests were performed, and both were positive. After verification, the mother reported the use of clindamycin gel for acne at one or two occasions in the previous year.

**Conclusion:** We believe this is the first reported case of clindamycin anaphylaxis where sensitization may have occurred through previous skin exposure.

### #28 Food-dependent exercise-induced anaphylaxis to almond in a 22-year-old male athlete

#### Kara Robertson^1^, Hannah Roberts^1^

##### Division of Allergy and Immunology, Department of Medicine, University of Western, London, ON, Canada

###### **Correspondence:** Kara Robertson

*Allergy Asthma Clin Immunol* 2020, **16(Suppl 1):**#28

**Background:** Food-dependent exercise-induced anaphylaxis (FDEIA) is a rare subtype of anaphylaxis that develops in association with physical exertion within a few hours of ingestion of a causative food. In this condition, neither food ingestion nor exercise alone is sufficient to trigger anaphylaxis. Although rare, this is an important condition to recognize, as it can lead to death.

**Case presentation:** A healthy 22-year-old male athlete presented with anaphylaxis requiring epinephrine administration in the emergency room. The episode occurred within 30 min of consuming almonds immediately following exercise. He consumed the same brand and quantity of almonds the same week, in the absence of physical activity, without issue. He also exercised at the same intensity on several occasions that week, in the absence of almond consumption, without adverse reaction. Skin prick testing was positive to almond, along with almond serum-specific IgE, confirming almond to be the culprit allergen. Skin prick testing for environmental aeroallergens was positive for dust mite, grass pollen, and cat, but negative to birch. The patient was successful with oral food challenge to almond in hospital.

**Conclusions:** This is the first report of FDEIA isolated to almond in a patient without birch sensitization or food pollen syndrome. There is only one published case report of FDEIA to almond in a female patient with food pollen syndrome, sensitized to birch. Our case eliminates food pollen syndrome as a possible cause of anaphylaxis in a patient sensitized to almond. This case is an important reminder that although rare, FDEIA exists and confirming a diagnosis can lead to life-saving preventative strategies. As tree nuts, especially almond, are not commonly associated with FDEIA, this case will serve to expand our differential for anaphylaxis and contribute to current allergy literature.

### #29 Cashew food allergy causing anaphylaxis and pancreatitis in a young Japanese man

#### Juan C. Ruiz^1^, Amin Kanani^2^

##### ^1^Clinical Allergy and Immunology Program, University of British Columbia, Vancouver, BC, Canada; ^2^Department of Medicine, University of British Columbia, Vancouver, BC, Canada

###### **Correspondence:** Juan C. Ruiz

*Allergy Asthma Clin Immunol* 2020, **16(Suppl 1):**#29

**Background:** Food induced pancreatitis (FIP) is a very rare entity, only 17 cases have been published, with half coming out of Japan, and most cases had IgE sensitization to the culprit food [1]. The pathophysiology of this condition is not well understood, proposed mechanisms are activation of pancreatic enzymes during anaphylaxis, or localized eosinophilic gastroenteritis at the Ampulla of Vater.

**Case presentation:** An 18-year-old Japanese male with allergies to peanuts, tree nuts and fish, had an accidental exposure to cashews and developed anaphylaxis with symptoms of vomiting, diarrhea, abdominal pain, urticaria and throat tightness. He was treated with epinephrine, dimenhydrinate, buscopan, ondansetron, and prednisone. He continued to have severe epigastric pain 7 h after ingestion and was found to have pancreatitis based on a lipase of 6104 U/L (0-393 U/L) and a CT scan showing an edematous pancreas with multifocal non-enhancing areas. Subsequent ultrasounds did not show ductal dilation or gall stones. He did not have eosinophilia or abnormal IgG subclasses. He had 3 beers, 2 days prior to onset of symptoms, and his liver enzymes were normal. Symptoms resolved 3 days after ingesting the cashews, and his lipase was normal after 5 days. Cashew skin prick testing was 10 mm wheal and specific IgE 72.3 KU/L.

**Conclusion:** We believe this is the first case report of cashew FIP. There is no biochemical or clinical evidence of autoimmune, alcoholic, gall stone and drug induced pancreatitis. The patient alcohol’s consumption was minimal. The medications described above are not associated with pancreatitis. In the future, practitioners should be aware of FIP in patients with persistent abdominal pain after a food anaphylaxis event.

#### **Reference**

1. Ogura K, Iikura K, Yanagida N, Sato S, Ebisawa M. Two patients with acute pancreatitis after undergoing oral food challenges. J Allergy Clin Immunol Pract. 2016;4(5):984–6.

### #30 A novel BTK gene mutation, T354I, in a patient with X-linked agammaglobulinemia (XLA)

#### Saghar Sadeghi^1^, Amin Kanani^2^

##### ^1^Department of Allergy and Immunology, University of Western Ontario, London, ON, Canada; ^2^Division of Allergy and Immunology, University of British Columbia, Vancouver, BC, Canada

###### **Correspondence:** Saghar Sadeghi

*Allergy Asthma Clin Immunol* 2020, **16(Suppl 1):**#30

**Background:** XLA is a hereditary primary immunodeficiency that results from Bruton’s tyrosine kinase (*BTK*) gene mutations leading to early–onset agammaglobulinemia and recurrent infections. There have been over 1000 mutations in BTK gene reported to be responsible for XLA. These mutations lead to defective B-cell development, profound deficiency of immunoglobulins and an increased susceptibility to infections.

**Case report:** A 55 year old gentleman presented to our clinic with recurrent sinopulmonary inections. At age 4 he was found to have hypogammaglobulinemia and was diagnosed with common variable immunodeficiency (CVID) and was treated with IVIG; despite this he continued to have recurrent infections. Blood work from 2009 had revealed no mature B-cells and therefore the suspicion of X-linked agammaglobulinemia was raised. BTK gene analysis was done revealing a novel T354I missense mutation in the BTK gene, reported as highly likely to be associated with XLA.

**Discussion:** BTK mutations in XLA consist of missense mutations, small insertion and deletions, Splice-site mutations, and nonsense mutations. The patient we describe was found to have a novel T354I missense mutation of BTK gene. T354I is a non-conservative amino acid substitution in that a polar Threonine residue is replaced with a non-polar Isoleucin at a position conserved across mammals in the SH2 domain of BTK. Early diagnosis, treatment, and genetic counseling are essential to reducing mortality and morbidity. This case emphasizes that suspicion for XLA and gene-study is essential for patients presenting with clinically overlapping disorders that would include XLA as a differential diagnosis.

Consent was obtained from the patient for this case report

### #31 A rare case of hereditary angioedema in a patient with systemic lupus erythematosus

#### Saghar Sadeghi, Hannah Roberts

##### Division of Allergy and Immunology, Department of Medicine, University of Western, London, ON, Canada

###### **Correspondence:** Saghar Sadeghi

*Allergy Asthma Clin Immunol* 2020, **16(Suppl 1):**#31

**Background:** Hereditary angioedema (HAE) is a rare genetic condition characterized by recurrent episodes of angioedema. Angioedema results from a bradykinin-mediated process, although patients are often treated as if this is histamine induced, resulting in ineffective management. Although rare, this condition is important to recognize as laryngeal edema can lead to fatal asphyxiation. The most commonly characterized forms of HAE arise from deficiency or dysfunction of C1 inhibitor. HAE types 1 and 2 are secondary to autosomal dominant mutations in the SERPING1 gene, with about 25% of mutations being de novo. HAE rarely has been associated with systemic lupus erythematosus (SLE).

**Case presentation:** A 26 year old female presented with a ten year history of undiagnosed, recurrent angioedema involving the larynx, abdomen, genitourinary tract, and face. There was no family history of angioedema. She subsequently experienced inflammatory arthritis, a malar rash, recurrent aphthous ulcers, and a positive ANA, all suggestive of lupus. Investigations confirmed a low C1 esterase inhibitor level on two occasions.

**Conclusion:** The association between SLE and HAE is rare. We report an interesting case of a patient with SLE and a classic presentation of HAE, versus acquired angioedema. Those rarely reported with Type 1 HAE and SLE are most often females and symptomatic (prominent gastrointestinal symptoms) with HAE, before developing lupus. This is consistent with our case. We suspect our patient has a de novo mutation and genetic testing is pending. Our case adds to the small body of literature recognizing a complex patient population with HAE and lupus, and will serve to contribute to current literature and assist in timely diagnosis.

### #32 A systemic allergic reaction in oral challenge-confirmed hazelnut “tolerant” individual

#### Sydney A. Scheffler^1^, Vince Wu^1^, Wardha Wardha^1^, Tarin T. Moni^1, 2^, Kristiina E. Frechette^1,2^, Jason A. Ohayon^1,3^

##### ^1^Hamilton Allergy, Hamilton, ON, Canada; ^2^McMaster University, Hamilton, ON, Canada; ^3^Department of Pediatrics, McMaster University, Hamilton, ON, Canada

###### **Correspondence:** Sydney A. Scheffler

*Allergy Asthma Clin Immunol* 2020, **16(Suppl 1):**#32

**Background:** Oral challenges remain the gold standard for hazelnut allergy diagnosis. However, an international standard for dosing and interpretation has yet to be developed. Oral challenge (OC) dosing is often clinic-specific and, in rare cases, may produce false negative results.

**Case presentation:** A 10-year-old female with skin test positivity to hazelnut presented to an allergy clinic for a hazelnut OC. Component testing revealed an elevated Cor A8 at 9.49 units, however the remaining components, Cor A1, Cor A9, and Cor A14, were undetectable. The patient was skin prick tested to hazelnut (10 mm), raw hazelnut (9 mm), and histamine (4 mm). The standard OC dosing in the clinic is 0.5 g, 1 g, 2 g, and 4 g at 15-min intervals with a 30-minmonitoring period after the final dose. The patient went on to tolerate 7.5 g of hazelnut flour (1.34 g hazelnut protein) in clinic. She was labeled as hazelnut-tolerant and discharged home. Four to 5 h after the OC, the patient consumed four hazelnut chocolate balls at home. Approximately 30 min after consumption, the patient complained of nausea, congestion, and facial swelling. The patient was treated with diphenhydramine and symptoms resolved. Analysis of the hazelnut chocolate ball protein content identified a total of 4 g hazelnut protein (1 g per ball).

**Conclusions:** The clinic has adapted their OC protocol to add a fifth dose of 8 g of hazelnut flour (3.3 g pure hazelnut protein) to reduce the risk of false negative challenges where threshold dosing in hazelnut allergic children may be higher. This case emphasizes the need for an international standard for hazelnut OC dosing.

Written informed consent for publication of their clinical details was obtained from the parent of the patient. A copy of the consent form is available for review by the Editor of this journal.

### #33 Playing with fire: the risks associated with intradermal cephalosporin testing

#### Wardha Wardha^1^, Vince Wu^1^, Sydney A. Scheffler^1^, Kristiina E. Frechette^1,2^, Tarin T. Moni^1,2^, Jason A. Ohayon^2^

##### ^1^Hamilton Allergy, Hamilton, ON, Canada; ^2^McMaster University, Hamilton, ON, Canada

###### **Correspondence:** Wardha Wardha

*Allergy Asthma Clin Immunol* 2020, **16(Suppl 1):**#33

**Background:** Cefuroxime (Cf) is a second generation Cephalosporin, which is widely used to treat bacterial infections. In patients with suspected allergic presentation to Cf, skin (prick and intradermal) testing may be used to investigate allergy. Testing of Cf allergy, it is not without its risks.

**Case presentation:** A 47-year-old female with suspected allergic reactions to Cefuroxime (lip angioedema), Clarithromycin and Sulfa-based medications presented for allergy assessment in a community clinic. The patient underwent intradermal (ID) testing to a dose of 10 mg/ml of Cefuroxime with a flare response of 15 mm. Within minutes, the patient felt unwell with generalised erythema, pruritus and dizziness. She was identified as having an anaphylactic reaction and treated with intramuscular (IM) epinephrine 0.5 mg initially. Initial response to IM Epinephrine was minimal and patient remained unwell. The patient’s systolic blood pressure dropped to 105 mmHg from initial 145 mmHg. Patient was placed in supine position with legs raised and insertion of IV normal saline for resuscitation. 911 was subsequently called. Patient was also provided with oxygen and Diphenhydramine 50 mg IM. The patient’s blood pressure recovered to 135 mmHg. The emergency response arrived and the patient was transported to the emergency room for further monitoring which was uneventful.

**Conclusion:** The case report highlights the risk of anaphylaxis in ID Cf testing and the role of IV fluids in case of hypotension persisting post IM Epinephrine treatment. In clinics choosing to test with ID cephalosporins, it would be advised to ensure IV fluids are available for fluid resuscitation.

**Statement of consent:** Written informed consent for publication of their clinical details were obtained from the patient. A copy of the consent form is available for review by the Editor of this journal.

### #34 Anaphylaxis to cilantro during an oral food challenge

#### Andrew Wong-Pack^1^, Erica Bernstein^2^, Samira Jeimy^2^

##### ^1^Schulich School of Medicine and Dentistry, Western University, London, ON, Canada; ^2^Division of Clinical Immunology and Allergy, Western University, London, ON, Canada

###### **Correspondence:** Andrew Wong-Pack

*Allergy Asthma Clin Immunol* 2020, **16(Suppl 1):**#34

**Background:** Cilantro is a member of the Apiaceae family, which includes carrots, parsley, aniseed and fennel seeds. Allergens are present in both the leaves and seeds of the plant. Reported hypersensitivity reactions include stomatitis, dermatitis, and contact urticaria.

**Case presentation:** A 20 year old female underwent an oral challenge for cilantro. She had contact urticaria with exposure to cilantro at the workplace, and urticaria with emesis when eating salsa containing cilantro. She had dermatographism resulting in equivocal cilantro skin prick testing. She had negative aeroallergen testing, including with birch and mugwort pollen. We did not have access to cilantro specific IgE. The graded oral challenge began with one-eighth of a cilantro leaf. In 20 min she developed facial and neck erythema and urticaria, as well as mouth pruritis and lip tingling. She then developed emesis, diarrhea, and chest tightness. Symptoms persisted despite receiving two doses of epinephrine 0.5 mg IM, cetirizine 10 mg SL and 4 puffs of salbutamol. She was transferred to urgent care, where she also received 1 L of IV normal saline, methylprednisolone 125 mg IV and ranitidine 50 mg IV. The patient was instructed to avoid ingestion of cilantro and carry epinephrine auto-injectors at all times.

**Conclusions:** Allergy to cilantro is rarely described; skin testing and serum IgE testing have unknown prognostic value. This is the first report of anaphylaxis to isolated fresh cilantro during a graded oral challenge. Clinicians should be aware of the risk of anaphylaxis during oral food challenges even with minimal protein content and act promptly when reactions occur.

**Statement of consent:** Consent to publish was obtained from the patient.

### #35 Drug-induced enterocolitis syndrome (DIES) with pantoprazole in an adult patient

#### Geneviève Bouvette^1^, Nina Verreault^2^, Nathalie Gagnon^3^, Aubert Lavoie^2^

##### ^1^Division of Internal Medicine, Department of Medicine, Centre Hospitalier de l’Université de Montréal (CHUM), Montreal, QC, Canada; ^2^Division of Allergy and Clinical Immunology, Department of Medicine, Centre Hospitalier de l’Université Laval (CHUL), Quebec, QC, Canada; ^3^Division of Internal Medicine, Department of Medicine, Hôpital Saint-François d’Assise (HSFA), Quebec, QC, Canada

###### **Correspondence:** Geneviève Bouvette

*Allergy Asthma Clin Immunol* 2020, **16(Suppl 1):**#35

**Background:** Drug-induced enterocolitis syndrome (DIES) is a rare type of non-IgE-mediated hypersensitivity reaction to drugs that shares clinical features with a well-recognized entity: food protein-induced enterocolitis syndrome (FPIES). In the acute setting, both DIES and FPIES manifest as delayed-onset profuse vomiting and diarrhea which can lead to dehydration and, more severely, hypovolemic shock (1). To our knowledge, only a few cases of enterocolitis syndrome caused by drugs have been reported, exclusively with amoxicillin and amoxicillin-clavulanate. To date, there is no report of DIES provoked by proton pump inhibitors (PPIs).

**Case presentation:** We report the case of a 69-year-old female who developed, within a few hours after taking pantoprazole, two episodes of severe abdominal pain, vomiting and bloody diarrhea leading to hypovolemic shock with renal insufficiency. Complete investigation showed no infectious, inflammatory or ischemic causes for her acute gastro-intestinal (GI) symptoms. An oral challenge to pantoprazole was performed and was clearly positive for reproducing the exact same violent GI symptoms 1 h after completing the test. There were no respiratory or cutaneous signs and symptoms. Abdominopelvic CT-scan performed during the first episode was positive for enteritis showing small intestine edema.

**Conclusions:** To our knowledge, this is the first case of DIES secondary to a PPI. It demonstrates the seriousness the course of this disease can take with life-threatening hypovolemic shock. Awareness and recognition of this entity, in its mild or more severe forms, is thus important in the evaluation of a patient presenting with acute GI symptoms, considering possible drug reactions.

**Statement of consent:** Consent to publish was obtained from the patient involved in this study.

#### **Reference**

1. Van Thuijl AOJ, Landzaat LJ, Liem O, Emons JAM, Arends NJT. Drug-induced enterocolitis syndrome (DIES): A clinical entity that deserves more awareness. Ann Allergy Asthma Immunol. 2019;122(5):538–9.

## Food allergy/anaphylaxis

### #36 Anaphylaxis in oral allergy syndrome: a case series

#### Khaldon F. Abbas^1^, Alexander Shusterman^1^, Gordon Sussman^1,2^

##### ^1^Gordon Sussman Clinical Research Inc., Toronto, ON, Canada; ^2^University of Toronto, Toronto, ON, Canada

###### **Correspondence:** Khaldon F. Abbas

*Allergy Asthma Clin Immunol* 2020, **16(Suppl 1):**#36

**Background:** Oral allergy syndrome (OAS) is an IgE mediated allergic reaction resulting from cross reactivity of pollens, mainly birch and ragweed, with fruits, vegetables, and tree nuts [1]. It is generally recognized as a benign syndrome presenting with local tingling and pruritus of lips, mouth, and throat. We report 12 cases of anaphylactic reactions following consumption of foods known to cross react with pollens in patients mainly presenting with OAS.

**Methods:** Charts of patients diagnosed with OAS were evaluated for clinical symptoms fitting the diagnostic criteria of anaphylaxis. Patient charts with reported anaphylactic reactions were isolated and summarized for demographics, clinical history, symptoms, food triggers, and skin prick test (SPT) results.

**Results:** Out of 130 charts reviewed, 12 were isolated with anaphylactic reactions. The mean age of patients was 41 years (range 18–64, M:F 5:7). All had positive SPT to birch, have a history of seasonal allergies, and reported local mouth tingling/itching when in contact with certain foods known to cross react with pollens. These patients developed severe multi system reactions following consumption of large quantities of specific food trigger. Of the 12 patients, 10 reported urticaria, 8 reported throat tightness, 8 reported gut symptoms, 7 reported shortness of breath/wheezing, and 2 reported loss-of-consciousness. Four patients were physically exercising prior to developing anaphylaxis reactions. The specific foods vary among patients and include tree nuts, avocado, grapes, carrots, apple, peaches, pomegranate, and nectarine.

**Conclusion:** OAS is an allergic reaction to certain fruits, vegetables, and nuts resulting from cross reactivity of pollens. Generally, the symptoms do not progress beyond the mouth. However, severe multi system allergic reactions are possible. The quantity of the pollen cross reacting food consumed might be a factor in the severity of the allergic response. Exercise can potentially be a co-factor to the development of severe allergic reactions in patients with OAS.

#### **Reference**

1. Sussman G, Sussman A, Sussman D. Oral allergy Syndrome. CMAJ. 2010;182:1210–11.

### #37 Defining food allergy documentation in Canadian primary care practices

#### Elissa M. Abrams^1^, Nerissa N. Nankissoor^2^, Alexander G. Singer^3^

##### ^1^Department of Pediatrics, Section of Allergy and Clinical Immunology, University of Manitoba, Winnipeg, MB, Canada; ^2^UCD School of Medicine, University College Dublin, Dublin, Ireland; ^3^Department of Family Medicine, University of Manitoba, Winnipeg, MB, Canada

###### **Correspondence:** Elissa M. Abrams

*Allergy Asthma Clin Immunol* 2020, **16(Suppl 1):**#37

**Background:** To date, prevalence data for food allergy in Canada is limited to self-report, with a previous survey documenting self-reported food allergy prevalence of 8.1%. However, limitations inherent in self-reported approaches suggest this maybe an overestimation. Our study aims to determine the prevalence of physician reported food allergies using Electronic Medical Record (EMR) data from providers participating in the Manitoba Primary Care Research Network (MaPCReN). We expect a lower prevalence of food allergies compared to the self-reported rate.

**Methods:** An algorithm detecting documentation of food allergy was constructed and validated, based on 2817 unique chart entries considered a food allergy out of 4488 possible allergy entries. The MaPCReN repository contains 221,132 patients of all ages (52.4% females) receiving primary care in Manitoba, Canada. Patients with food allergies were identified by the constructed algorithm. Descriptive statistics assessed for food allergy prevalence and a multivariable logistic regression model determined the association with patient, provider and practice variables.

**Results:** 1.4% of Manitobans have a documented food allergy, of which 61.4% have one or more comorbidities (asthma, depression, diabetes, hypertension, autism, or ADHD). Of those with food allergies, 44.8% (P = <0.0001) and 34.3% (P = <0.0001) have documentation of asthma and eczema, respectively. Individuals with food allergies have 1.8 times higher odds of an eczema diagnosis (CI 1.47–2.11%) and 2.1 higher odds of having one or more commodities (CI 1.89–2.41) compared to patients without food allergies.

**Conclusions:** Manitoba EMR derived data revealed a lower prevalence of food allergy than previously reported in a comparable Canadian study which relied on patient self-reporting (8.1%). The algorithm created in this study will be applied nationally within the Canadian Primary Care Sentinel Surveillance Network (CPCSSN), to determine Canada’s national prevalence of food allergy and investigate geographical variation food allergy prevalence.

### #38 Management of paediatric allergic reaction: practice patterns of Canadian emergency physicians

#### Caitlin Prendergast^1,2^, Amy Plint^1,2,3^, Ken Tang^3^, Gina Neto^1,2^, Waleed Alqurashi^1,2,3^

##### ^1^Division of Emergency Medicine, Children’s Hospital of Eastern Ontario, Ottawa, ON, Canada; ^2^Department of Pediatrics, University of Ottawa, Ottawa, ON, Canada; ^3^Children’s Hospital of Eastern Ontario Research Institute, Ottawa, ON, Canada

###### **Correspondence:** Waleed Alqurashi

*Allergy Asthma Clin Immunol* 2020, **16(Suppl 1):**#38

**Objectives:** The literature indicates that the incidence of anaphylaxis is increasing and that there are significant deficiencies in both recognition and management. We aimed to examine the magnitude of these gaps in Canadian pediatric emergency medicine (PEM).

**Methods:** We conducted a cross-sectional, self-administered survey of the Pediatric Emergency Research Canada (PERC) physician database. The survey tool was developed through a literature review to identify recurring themes of gaps in anaphylaxis diagnosis and management. The final tool contained four scenarios; three scenarios featured each of the National Institute of Allergy and Infectious Diseases (NIAID) anaphylaxis criteria, separately, and fourth case of non-anaphylactic allergy was added to further assess the diagnostic ability of participants. Multiple-choice questions associated with each scenario addressed diagnosis, management and disposition. We also inquired about the type and availability of anaphylaxis order sets and patient education resources in the ED.

**Results:** Of the 214 members invited to participate in the survey, 152 (71%) responded. Anaphylaxis was accurately recognized 93%, 82% and 99% of the time for the NIAID criteria 1 through 3, respectively. When anaphylaxis was recognized, epinephrine was prescribed in each case 96%, 95% and 72% of the time, respectively. Of all respondents, 115 (75%) accurately diagnosed all three cases of anaphylaxis, and of these respondents, 81 (53%) treated anaphylaxis with epinephrine each time. Of the 13 PERC centers, 4 (30%) reported having pre-printed order sets, and only one site indicated having patient education and discharge resources.

**Conclusions:** The majority of respondents recognized cases of anaphylaxis, however a substantial portion demonstrated gaps in management that may adversely impact this vulnerable population. The recognition of anaphylaxis without cutaneous or pulmonary findings and systematic treatment with epinephrine were the main gaps identified. Nationwide, there is a lack of structured patient education and discharge materials.

### #39 Usability evaluation of the Canadian anaphylaxis emergency plan: opportunity for improvement

#### Ernie Avilla^1^, Susan Waserman^1^, Abeer Hegazi^1^, Laurie Harada^2^, Joni Huang^2^, Monika Kastner^2^

##### ^1^Division of Clinical Allergy and Immunology, Department of Medicine, McMaster University, Hamilton, Canada; ^2^Food Allergy Canada, Toronto, Canada; ^3^Knowledge Translation and Implementation, North York General Hospital, Toronto, Canada

###### **Correspondence:** Ernie Avilla

*Allergy Asthma Clin Immunol* 2020, **16(Suppl 1):**#39

**Background:** Few patients have an anaphylaxis action plan even though they are advocated for routine use. Evidence for the effectiveness of action plans as part of long-term anaphylaxis management is lacking. Our study aim was to conduct a usability evaluation of the Canadian Anaphylaxis Emergency Plan (AEP) to determine how well it meets usability needs and whether it can facilitate appropriate emergency response to anaphylaxis.

**Methods:** The usability study employed one-on-one simulation sessions with (i) parents or caregivers of children with known allergies (age < 18 years) at risk for anaphylaxis; and (ii) school teachers to test the use of the AEP; all able of self-administer an epinephrine auto-injector (EAI). Our primary outcome was the correct identification of situations when an EAI was most appropriate to administer (using the AEP as a guide). We used two standardized patient scenarios to measure this outcome using an advanced patient simulator (SimMan^®^): Case A (not anaphylaxis) and Case B (anaphylaxis). Participants were exposed to both cases in random order. A moderator collected data using a standardized checklist including correct handling, initiation and site administration of an EAI placebo device). Participants also completed a validated, 10-item System Usability Scale (SUS).

**Results:** Sixteen individuals (5 children, 7 parents, 4 teachers) participated. The use of the AEP assisted the correct identification of anaphylaxis (and indication for administering an EAI) in 75% of participants (Case A) and 85% of participants (Case B). The SUS was completed by adult participants (n = 9); the overall mean score was 72.2 (SD 11.9), which represents a subjective grade of C+ (below average).

**Conclusion:** Our work contributes to the limited evidence of the effectiveness of AEPs for self-management of anaphylaxis. Our findings indicate that the Canadian AEP needs to be improved to optimize its use.

### #40 Exercise-associated anaphylaxis co-factors and epinephrine administration

#### Sabrina Bartolucci^1^, Sofianne Gabrielli^1^, Ann Clarke^2^, Edmond S. Chan^3^, Julia Upton^4^, Andrew O-Keefe^5^, Harlery Eisman^6^, Moshe Ben-Shoshan^1^

##### ^1^Division of Allergy and Clinical Immunology, Department of Pediatrics, Montreal Children’s Hospital, McGill University Health Centre, Montreal, QC, Canada; ^2^Division of Rheumatology, Department of Medicine, Cumming School of Medicine, University of Calgary, Calgary, AB, Canada; ^3^Division of Allergy and Immunology, Department of Pediatrics, BC Children’s Hospital, University of British Columbia, Vancouver, BC, Canada; ^4^Division of Immunology and Allergy, The Hospital for Sick Children, Department of Paediatrics, University of Toronto, Toronto, ON, Canada; ^5^Department of Pediatrics, Faculty of Medicine, Memorial University, St. John’s, Newfoundland & Labrador, Canada; ^6^Department of Emergency Medicine, Montreal Children’s Hospital, McGill University Health Centre, Montreal, QC, Canada

###### **Correspondence:** Sabrina Bartolucci

*Allergy Asthma Clin Immunol* 2020, **16(Suppl 1):**#40

**Background:** Exercise is reported to be a co-factor contributing to anaphylaxis severity and may also play a major role in certain forms of anaphylaxis including exercise induced anaphylaxis (EIA) and food dependent exercise induced anaphylaxis (FDEIA). There is little data on the role of exercise in large scale studies. We evaluate the sociodemographic, clinical characteristics and management of exercise-associated anaphylaxis cases, with a focus on food triggers and the management as part of the Cross-Canada Anaphylaxis Registry (CCARE).

**Methods:** Through the CCARE study, between April 2011 and April 2019, data was collected through a standardized questionnaire given to patients presenting to the Montreal Children’s Hospital Emergency Department (MCH ED) with anaphylaxis. Multivariate logistic regression was used to estimate factors associated with epinephrine administration in exercise-associated anaphylaxis.

**Results:** Among 2092 anaphylactic cases presenting to the MCH ED, 90 of the cases occurred in association with exercise, of which 61% were male, and the median age was 11.9 (IQR: 8.01, 14.96). Among them, the most common reported anaphylaxis trigger was food (n = 48, 53.33%), of which peanuts were the most common trigger (10, 20.83%), followed by soy (4, 8.33%) and milk (4, 8.33%). Of the peanut triggered cases, 30% did not have a known peanut allergy, whereas in the soy and milk triggered individuals, 75% and 50% respectively did not have a known allergy. Patients were more likely to receive epinephrine prior to arrival if there was a known food allergy, odds ratio (OR) 1.75 (95% CI 1.47, 2.10).

**Conclusions:** Food was frequently a reported trigger for exercise-associated anaphylaxis and epinephrine was more likely to be administered prior to arrival at the hospital in those who had a known food allergy to the trigger. Educational programs promoting the use of epinephrine in cases associated with exercise are required.

**Ethics statement:** The study was approved by the McGill University Health Center’s Research Ethics Board, approval number 10-203 GEN.

### #41 Reducing parental anxiety during pediatric oral food challenges: a randomized control trial of deep breathing exercises

#### Chantel E. Canessa^1^, Lianne Soller^2^, Sharon To^4^, Elaine Hsu^2^, Ingrid Baerg^2^, Kyla J. Hildebrand^2^, Tiffany Wong^2^, Raymond Mak^3^, Catherine M. Biggs^2^, Theresa A. Newlove^4,5^, Edmond S. Chan^2^

##### ^1^Division of Specialty Medicine, Department of Pediatrics, BC Children’s Hospital, Vancouver, BC, Canada; ^2^Division of Allergy & Immunology, Department of Pediatrics, University of British Columbia, BC Children’s Hospital, Vancouver, BC, Canada; ^3^Division of Allergy & Immunology, Department of Medicine, University of British Columbia, BC Children’s Hospital, Vancouver, BC, Canada; ^4^Department of Psychology, BC Children’s and Women’s Hospital and Sunny Hill Centre, Vancouver, BC, Canada; ^5^Adjunct Professor, Department of Psychology, University of British Columbia, Vancouver, BC, Canada

###### **Correspondence:** Chantel E. Canessa

*Allergy Asthma Clin Immunol* 2020, **16(Suppl 1):**#41

**Background:** Parents have increased anxiety associated with an oral food challenge (OFC). However, no research has been conducted to determine effectiveness of coping strategies for parents during an OFC. This study sought to determine effectiveness of a 5-min deep breathing intervention (compared with standard of care) in reducing parental anxiety on the day of their child’s OFC.

**Methods:** Parents of children with food allergy who underwent an OFC at BC Children’s Hospital were randomized to receive deep breathing exercises (intervention group) or standard of care (control group). Effects of the deep breathing intervention were measured objectively with a biofeedback device at three time points: pre-intervention, post-intervention before first dose of food, and post-OFC, and subjectively with the State Trait Anxiety Inventory-State anxiety (STAI-S) pre-intervention and post-OFC. Data analysis included an independent t-test to compare the effects of the intervention on anxiety between groups and correlation between the change in anxiety (subjective vs objective) from pre-intervention to post-OFC.

**Results:** Of 118 parents approached, 92 participated (78.0%); 54 received the intervention and 38 were controls. Baseline STAI-S scores were 46.1 (intervention) and 47.9 (control). There was no significant difference in subjective anxiety between the two groups pre-intervention to post-OFC (difference 0.64, 95%CI − 1.76, 3.04). There was no significant difference in objective anxiety between groups at any of the three time points. There was weak correlation between the change in anxiety (subjective vs objective) from pre-intervention to post-OFC (r^2^ = 0.016).

**Conclusions:** Deep breathing did not significantly reduce parental anxiety. Parents reported moderate to high anxiety before their child’s OFC, which highlights the importance of screening for anxiety in clinical practice. Our study is limited in that the intervention was 5 min long, and some parents who declined participation may have had higher anxiety. Further studies incorporating coping strategies to reduce parental anxiety during an OFC are needed.

### #42 Evaluating new processing methods and their effects on peanut allergenicity

#### Casey G. Cohen, Wei Zhao, Bertrand J. Jean-Claude, Bruce D. Mazer

##### Research Institute of the McGill University Health Centre, Montreal, QC, Canada

###### **Correspondence:** Casey G. Cohen

*Allergy Asthma Clin Immunol* 2020, **16(Suppl 1):**#42

**Background:** Peanut allergy is considered the most severe of all food allergies as it is the leading cause of fatal anaphylaxis. Evidence suggests that the allergenicity of peanuts is significantly increased in its roasted form when compared with raw. The aim of this project is to develop alternative processing methods to decrease the allergenicity of peanut.

**Methods:** Advanced Glycation End products (AGEs) are considered to be the main cause of increased allergenicity. Peanuts were ground into a paste and dissolved in n-hexane for defatting. Protein extracts from raw, roasted (150 °C, 30 min), boiled (100 °C, 2 h in water) and autoclaved (136 °C, 2.15 atm, 30 min) peanuts were used to quantify IgE binding via ELISA using serum samples from peanut-sensitive patients. Western Blot analyses were then carried out on the panel of protein extracts using an antibody specific for Ara h 2.

**Results:** IgE-binding assays of the extracts revealed no difference in IgE binding between raw and roasted or boiled peanuts but revealed a significant difference (p < 0.05) between raw and autoclaved peanuts. An absence of signal in the Western Blot analysis indicates potential degradation of Ara h 2 in autoclaved peanut samples. These results were consistent following roasting of the peanut before or after autoclaving, demonstrating a dominant effect of autoclaving over roasting in the context of IgE-binding.

**Conclusion:** In contrast to other findings, our results indicate no relevant difference in IgE binding between raw, roasted or boiled peanuts. However, a significant difference in IgE-binding and in Ara h 2 detection was found between autoclaved and raw peanut proteins, a dominant effect over roasting. Further analysis is required to determine what effect autoclaving has at the molecular level.

### #43 Quality-adjusted life-year gain with oral immunotherapy: preliminary results from a real-life cohort

#### Élise Dufresne^1^, Thomas G Poder^2,3^, Kathryn Samaan^4^, Jonathan Lacombe-Barrios^4^, Louis Paradis^1,4^, Anne Des Roches^4^, Philippe Bégin^1,4^

##### ^1^Department of Medicine, Université de Montréal, Montreal, QC, Canada; ^2^Department of Family Medicine, Université de Sherbrooke, Sherbrooke, QC, Canada; ^3^UETMISSS and CRCHUS, CIUSSS de l’Estrie-CHUS, Sherbrooke, QC, Canada; ^4^Departement of Pediatrics, CHU Sainte-Justine, Université de Montréal, Montreal, QC, Canada

###### **Correspondence:** Philippe Bégin

*Allergy Asthma Clin Immunol* 2020, **16(Suppl 1):**#43

**Background:** The current data on the quality of life benefit of oral immunotherapy (OIT) is mostly based on disease-specific questionnaires (FAQLQ) that do not translate to quantifiable values. This is a major limit when advocating for resources in clinical context since this benefit cannot be compared to other disease states or other interventions. We have recently developed a conversion algorithm allowing translation of FAQLQ results into quality-adjusted life-years (QALY) based on validated generic questionnaire (SF-6D). The aim of this project was to quantify the gain in QALY after completing the desensitization phase of OIT based on this algorithm.

**Methods:** FAQLQ questionnaires were administered to patients and parents of a public academic OIT clinic as appropriate for age at time of referral for OIT and 3 months after reaching OIT maintenance doses, along with a modified version of the SF-6D using a unitary approach. SF-6Dv2 data were converted to QALY using preference weights developed by the University of Sheffield. FAQLQ data were converted to QALY using the previously developed conversion algorithm. Change in QALY values were compared with paired T-test.

**Results:** Of the 96 patients having reached the 3 months maintenance doses, 43 had completed the questionnaire as of June 2019. Of these, 21 had completed all relevant questions of the FAQLQ-PF (0 to 12 y-o). The average gain in QALY inferred from FAQLQ conversion was 0.052 ± 0.093 (p = 0.018). This change could not be detected using QALY values from repeated SF-6D questionnaire, with a mean improvement of 0.014 ± 0.170 (p = 0.64).

**Conclusions:** Generic questionnaires fail to discriminate gain in QALY from OIT in food allergy. The conversion algorithm allowing FAQLQ translation to QALY allowed for more reliable estimate, indicating 0.052 QALY gained, recurrent after desensitization.

The study was approved by CHU Sainte-Justine’s Ethics Board, approval number MP-21-2019-2088.

### #44 First anaphylactic reactions among children from the Cross-Canada Anaphylaxis REgistry (C-CARE)

#### Sofianne Gabrielli^1^, Ann Clarke^2^, Judy Morris^3^, Harley Eisman^4^, Jocelyn Gravel^5^, Rodrick Lim^6^, Edmond S. Chan^7^, Ran Goldman^8^, Julia Upton^9^, Andrew O-Keefe^10^, Jennifer Gerdts^11^, Moshe Ben-Shoshan^1^

##### ^1^Division of Allergy and Clinical Immunology, Department of Pediatrics, Montreal Children’s Hospital, McGill University Health Centre, Montreal, QC, Canada; ^2^Division of Rheumatology, Department of Medicine, Cumming School of Medicine, University of Calgary, Calgary, AB, Canada; ^3^Department of Emergency Medicine, Sacré-Coeur Hôpital, Montreal, QC, Canada; ^4^Department of Emergency Medicine, Montreal Children’s Hospital, McGill University Health Centre, Montreal, QC, Canada; ^5^Department of Pediatric Emergency Medicine, Centre Hospitalier Universitaire Sainte-Justine, Montreal, QC, Canada; ^6^Division of Pediatrics and Emergency Medicine, Children’s Hospital at London Health Science Centre, London, ON, Canada; ^7^Division of Allergy and Immunology, Department of Pediatrics, BC Children’s Hospital, University of British Columbia, Vancouver, BC, Canada; Department of Pediatric Emergency Medicine, BC Children’s Hospital, University of British Columbia, Vancouver, BC, Canada; ^9^Division of Immunology and Allergy, The Hospital for Sick Children, Department of Paediatrics, University of Toronto, Toronto, ON, Canada; ^10^Department of Pediatrics, Faculty of Medicine, Memorial University, St. John’s, Newfoundland & Labrador, Canada; ^11^Executive Director, Food Allergy Canada, Toronto, ON, Canada

###### **Correspondence:** Sofianne Gabrielli

*Allergy Asthma Clin Immunol* 2020, **16(Suppl 1):**#44

**Background:** Data on first anaphylactic reaction in children with no known food allergy is sparse. We aimed to assess the clinical characteristics and management of first anaphylactic reactions in children presenting to six Emergency Departments (EDs) across Canada.

**Methods:** Between April 2011 to May 2019, children presenting to six EDs in four Canadian provinces with anaphylaxis were recruited as part of the Cross-Canada Anaphylaxis REgistry (C-CARE). A standardized data form documenting symptoms, triggers and management was collected. Multivariate logistic regression was used to predict factors associated with epinephrine treatment among first anaphylactic reactions in children.

**Results:** Over an 8-year period, among 2701 children presenting with food-induced anaphylaxis, 1027 (38%) children presented with their first reaction. The main triggers were peanut (17%), tree nut (15%), and egg (11%) among all ages. Outside of the ED, epinephrine was used to treat 15% of cases and antihistamines in 40%. Inside in the ED, epinephrine was used in 56% of patients and antihistamines in 53%. When stratifying by age, the main triggers of first reactions in children less than 1 year old were egg (31%) and milk (15%); in children between 1 and 6 years old, peanut (24%) and tree nut (21%); in children between 7 and 12 years, other food (21%) and unknown food (18%); and in children 13 years and over, unknown food (23%) and other food (21%). First anaphylactic reactions triggered by egg were more likely to be treated without epinephrine (adjusted Odds Ratio (aOR) 1.11 (95%CI 1.01, 1.22)), while those reacting to milk (aOR 1.22 (95%CI 1.07, 1.39)) and with severe reactions (aOR 1.17 (95%CI 1.02, 1.34)), were more likely to be treated with epinephrine, while adjusting for age, sex, asthma, and location.

**Conclusion:** Difference between age groups for specific food triggering first anaphylactic reactions may be due to introduction patterns of different foods.

**Ethics statement:** The study was approved by the McGill University Health Centre’s Research Ethics Board, approval number 10-203 GEN.

### #45 Diagnosis of ibuprofen allergy through oral challenge

#### Judy Gaffar^1^, Sofianne Gabrielli^1^, Christine McCusker^1^, Christos Tsoukas^2^, Moshe Ben-Shoshan^1^

##### ^1^Division of Allergy Immunology and Dermatology, Montreal Children’s Hospital, Montreal, QC, Canada; ^2^Department of Medicine, Division of Allergy and Clinical Immunology, McGill University, Montreal, QC, Canada; Division of Experimental Medicine, The Research Institute of the McGill University Health Centre, McGill University, Montreal, QC, Canada

###### **Correspondence:** Judy Gaffar

*Allergy Asthma Clin Immunol* 2020, **16(Suppl 1):**#45

**Background:** Non-steroidal anti-inflammatory drugs (NSAIDs) are frequently implicated in hypersensitivity reactions in children. Given that Ibuprofen is often used to manage fever in children and given the absence of standardized skin tests in the diagnosis of NSAID allergy, drug challenges are the only available tool to establish the diagnosis of this drug allergy. We aimed to assess the risk of Ibuprofen allergy in patients through the graded oral challenge.

**Methods:** All children referred to the Montreal Children’s Hospital for suspected NSAIDs allergy were recruited for the LACTAAM study between January 2017 and June 2019. A standardized survey of treatment, symptoms, and associated factors was filled and an oral challenge (10% and 90% of the oral dose) was conducted. The families were contacted annually to inquire about subsequent NSAID use. Descriptive statistics were used to characterize the reactions.

**Results:** Thirty-five patients with a reported allergy to Ibuprofen were recruited. The majority of the reactions (65%) occurred within 1 h after using the medication and 89% occurred within the first 3 days of taking the drug. The most common symptom was angioedema (54%). All children underwent an oral challenge with ibuprofen and six patients (17.14%) had a positive challenge, five of which were immediate reactions and one non-immediate reactions. Among the six patients, symptoms were consistent with anaphylaxis in three (50%), and all three (100%) were treated with epinephrine. Among the 27 patients with negative oral challenge eligible for follow-up, 15 (55%) patients responded. Of the contacted patients, nine (60%) reported subsequent Ibuprofen use of which two patients (22.22%) reacted and had symptoms consistent with anaphylaxis.

**Conclusion:** Graded oral challenges can be used to diagnose an allergy to Ibuprofen. However, the challenges are associated with high risk of anaphylaxis and there is a 22% risk of subsequent reaction despite negative challenge in this small cohort.

**Ethics statement:** McGill University Health Center Research Ethics Board. REB # 12-084 PED.

### #46 An evaluation of logistical challenges associated with multi-food oral immunotherapy in a Canadian pediatric private practice clinic

#### Mariam A. Hanna^1,2^, Douglas P. Mack^1,2^

##### ^1^Halton Pediatric Allergy, Burlington, ON, Canada; ^2^Department of Pediatrics, McMaster University, Hamilton, ON, Canada

###### **Correspondence:** Mariam A. Hanna

*Allergy Asthma Clin Immunol* 2020, **16(Suppl 1):**#46

**Background:** As oral immunotherapy (OIT) becomes more commonplace in Canada, clinics will need to modify their primarily consultation-based practice in order to accommodate frequent follow-ups both during buildup and maintenance phases. Physicians will also need to adapt to the change in logistical burden associated with OIT. Clinical and nonclinical burdens will present barriers to physicians and patients and will affect accessibility to OIT.

**Methods:** As a quality improvement initiative, we have retrospectively reviewed patient charts, clinic schedules and daily patient email contact with OIT physicians in a private practice pediatric clinic offering multi-food OIT from May, 2017-May, 2019. We have also modelled future maintenance follow-up burden.

**Results:** We have demonstrated a significant increase in daily OIT-related after-hours/weekend email contact managed by physicians. Over a 1 year period, frequency of email contact increased from 3.5 emails per day to 14.5. This contact has increased with the number of enrolled patients. We also demonstrate a significant increase in frequency of oral graded challenges (OGCs) in our clinic related to increases in demand for OIT. Completed oral challenges have increased from a frequency of 2 per day to approximately 8 per day. We currently book up to 12 oral challenges per day and have performed 1980 OGCs in the 2 years. OIT up dose visit absenteeism related to patient illness or uncontrolled asthma was evaluated demonstrating a mean absentee rate of approximately 33%. Finally, modelling of follow-up appointments suggest that in 5 years, our clinic will need to accommodate between 12.5 and 16.3 40-h weeks simply seeing OIT follow-up patients on maintenance.

**Conclusions:** The logistical burden of OIT increases as clinics increase the number of patients treated. This burden will be felt by physicians, administrative staff, allied health and patients. These logistical challenges will affect accessibility to OIT and will present long term challenges.

### #47 Administration of epinephrine autoinjector by families during oral food challenge or in the community leads to greater confidence

#### Elaine Hsu^1,2^, Lianne Soller^1,2^, Timothy Teoh^2^, Ingrid Baerg^1,2^, Tiffany Wong^1,2^, Kyla J. Hildebrand^1,2^, Victoria E. Cook^1,2^, Catherine M. Biggs^1,2^, Lindsay Yaworski^2^, Scott B. Cameron^1^, Edmond S. Chan^1,2^

##### ^1^Division of Allergy and Immunology, Department of Pediatrics, Faculty of Medicine, University of British Columbia, Vancouver, BC, Canada; ^2^BC Children’s Hospital, Vancouver, BC, Canada

###### **Correspondence:** Elaine Hsu

*Allergy Asthma Clin Immunol* 2020, **16(Suppl 1):**#47

**Background:** Our previous study found that supervised administration of the epinephrine autoinjector (EAI) during an oral food challenge (OFC) increased confidence administering the EAI [1]. We sought to determine whether administering the EAI in the community since the OFC had a similar effect on confidence, and whether increased confidence was sustained over time.

**Methods:** Families who completed an OFC at BC Children’s Hospital were sent a follow up survey 1 year after the OFC, asking them whether they had administered an EAI since the OFC, and their confidence in administering it (1 = not very confident; 5 = very confident). Logistic regression was performed with change in confidence administering the EAI (positive vs. negative/zero) as the outcome, and administering the EAI (either during the OFC or during the 1-year follow-up (yes/no), as the predictor.

**Results:** Of 353 OFCs completed in 317 families between 2014 and 2017, 53/317 (15%) administered the EAI; 162/317 (51.1%) families completed the 1-year follow-up survey, of which 18/162 (11.1%) administered the EAI during follow-up. Confidence administering EAI increased from pre-OFC (3.25, 95%CI 3.15, 3.36) to follow-up (3.79, 95%CI 3.67, 3.93) for all families. However, families who administered the EAI during the OFC and/or follow-up were more likely to experience an improvement in confidence administering EAI from pre-OFC to follow-up (OR: 2.13, 95%CI 1.01, 4.52) compared with those who did not experience administering the EAI.

**Conclusions:** Our data show that regardless of OFC outcome, patient confidence administering the EAI improves after an OFC, and this is sustained 1 year later. Those who have administered an EAI during OFC or in the community are more likely to experience increased confidence, than those who do not have this experience. This indicates the importance of appropriate education and training together with the benefit of personal experience, for optimal patient empowerment.

#### Reference

1. Soller L, Teoh T, Baerg I, et al. Extended analysis of parent and child confidence in recognizing anaphylaxis and using the epinephrine autoinjector during oral food challenges. J Allergy Clin Immunol Pract. 2019;7(2):693–5.

### #48 Larger skin test wheal size associated with prolonged escalation phase during cow milk oral immunotherapy (CMOIT)

#### Danbing Ke^1^, Bruce Mazer^1^, Duncan Lejtenyi^1^, Liane Beaudette^1^, Edmond S Chan^2^, Ingrid Braeg^2^, Moshe Ben-Shoshan^1^

##### ^1^Division of Allergy and Immunology, Department of Pediatrics, McGill University, Montreal, QC, Canada; ^2^Division of Allergy and Immunology, Department of Pediatrics, University of British Columbia, Vancouver, BC, Canada

###### **Correspondence:** Danbing Ke

*Allergy Asthma Clin Immunol* 2020, **16(Suppl 1):**#48

**Background:** Most patients with cow’s milk (CM) allergy can be safely desensitized via a three-stage oral immunotherapy (rush, escalation and maintenance). The duration of CMOIT is mainly determined by the progress of updosing during escalation phase when adverse reaction often occurs. It remains challenging to identify individuals who are less responsive to standardized desensitization procedures. We sought to assess the association of skin prick test (SPT) wheal size and length of escalation phase for milk desensitization.

**Methods:** CM-allergic patients from two Canadian tertiary hospitals were enrolled. Following challenge and rush desensitization, each patient underwent weekly updosing at clinic visit and took daily dose at home between visits. Clinical data and skin prick test (SPT) were registered when targeted doses (initial, 6, 25, 125, 200 and 300 mL) were taken during escalation. Patient progress was charted and survival analysis was conducted to determine the cumulative probability of successful desensitization with regard to SPT wheal size at study entry.

**Results:** Of 79 patients, 42 were successfully desensitized over the escalation phase that ranged between 141 to 791 days (median of 232 days). In these patients, SPT wheal diameters decreased from a mean of 10.6 mm at the study entry to that of 5.7 mm at the end of updosing phase (P < 0.01). According to their SPT wheal size at study entry, the median time to achieve complete desensitization were 232, 259 and 552 days for groups with SPT ≥ 3 mm, SPT ≥ 8 mm, and SPT ≥ 15 mm respectively. Kaplan–Meier analysis revealed a cumulative probability of successful desensitization after 791 days at 100% for SPT ≥ 3, 96.1% for SPT ≥ 8 cm and 64.5% for SPT ≥ 15 (P = 0.0001).

**Conclusions:** Larger SPT is associated with prolonged CM desensitization. Strategies aiming to shorten dose escalation interval are required for patients with large SPT (≥ 15 mm) at study entry.

The study was approved by MUHC-RI’s Ethics Board, approval number PED-12-090 with http://www.clinicaltrials.gov registry no. NCT03644381.

### #49 Efficiency of peanut suspension with simple syrup compared to individual pre-weighted flour doses for oral immunotherapy

#### Hélène Leroux^1^, Alexandra Langlois^2^, Véronique Bastien^2^, Kathryn Samaan^2^, Jonathan Lacombe-Barrios^2^, Louis Paradis^2^, Anne Des Roches^1,2^, Philippe Bégin^1,2^

##### ^1^Sainte-Justine University Hospital Research Center, Montreal, QC, Canada; ^2^Allergy section, Department of pediatrics, Sainte-Justine University Hospital Center, Montreal, QC, Canada

###### **Correspondence:** Philippe Bégin

*Allergy Asthma Clin Immunol* 2020, **16(Suppl 1):**#49

**Background:** The ideal form of allergen to be used for oral immunotherapy (OIT) has yet to be defined. The approach favored in many clinical trials has been to prepare individual pre-weighted doses of allergen flour. However, this approach is time consuming and is technician-dependent. The aim of this study was to determine the efficiency and exportability of the use of peanut suspension in simple syrup compared with individual pre-weighted flour doses.

**Methods:** Total time for preparing 500 ml of peanut protein suspension in simple syrup at 50 mg/mL was measured, including the time needed for preparing the equipment, weighting peanut flour, mixing it with syrup, completing forms, distributing the peanut suspension in doses cups of 80 ml and cleaning the production space. The time needed to complete two prescriptions of 40 doses of 25 mg and 125 mg of peanut protein was also assessed. Procedures were first performed by an experienced dietetic technician and then repeated by an allergist with less experience in these tasks.

**Results:** When considering technician time for 1 month worth of doses at 25 mg protein, the peanut suspension represented a gain in efficiency of 2340% compared to individual pre-weighted doses. For the 125 mg dose, the efficiency was 470% better for the peanut suspension. When considering allergist time, the gain in efficiency was even more pronounced (3533% and 670% increase for doses of 25 mg and 125 mg, respectively).

**Conclusion:** Use of peanut suspension in simple syrup rather than pre-weighted individual doses can lead to major gain in efficiency. The level of efficiency is also less dependent on technician experience, making for an easily exportable solution for OIT clinics with minimal resources.

### #50 Increased risk of anaphylaxis during Halloween and Easter in Canadian children

#### Melanie Leung^1^, Sofianne Gabrielli^1^, Ann E Clarke^2^, Judy Morris^3^, Jocelyn Gravel^4^, Rodrick Lim^5^, Edmond S. Chan^6^, Andrew O-Keefe^7^, Jennifer Gerdts^8^, Greg Shand^1^, Xun Zhang^9^, Moshe Ben-Shoshan^1^

##### ^1^Division of Allergy and Clinical Immunology, Department of Pediatrics, Montreal Children’s Hospital, McGill University Health Centre, Montreal, QC, Canada; ^2^Division of Rheumatology, Department of Medicine, Cumming School of Medicine, University of Calgary, Calgary, AB, Canada; ^3^Department of Emergency Medicine, Sacré-Coeur Hôpital, Montreal, QC, Canada; ^4^Division of Pediatric Emergency Medicine, Centre Hospitalier Universitaire Sainte-Justine, Montreal, QC, Canada; ^5^Division of Pediatrics and Emergency Medicine, Children’s Hospital at London Health Science Centre, London, ON, Canada; ^6^Division of Allergy and Immunology, Department of Pediatrics, BC Children’s Hospital, University of British Columbia, Vancouver, BC, Canada; ^7^Department of Pediatrics, Faculty of Medicine, Memorial University, St. John’s, Newfoundland & Labrador, Canada; ^8^Executive Director, Food Allergy Canada, Toronto, ON, Canada; ^9^Centre for Outcomes Research and Evaluation, Research Institute of McGill University Health Centre, Montreal, QC, Canada

###### **Correspondence:** Melanie Leung

*Allergy Asthma Clin Immunol* 2020, **16(Suppl 1):**#50

**Background:** Anaphylaxis is a severe allergic reaction, mainly triggered by peanut and tree nut. There are no data on the risk of anaphylactic reaction at certain time points during the year, when children are more likely exposed to peanut/tree nut. We aimed to assess the risk of peanut/tree nut-triggered anaphylaxis on Halloween, Christmas, and Easter

**Methods:** From April 2011 to November 2018, our research team, working in six EDs in 4 provinces (Quebec, Ontario, Newfoundland and Labrador, and British Columbia), documented the date of presentation to the ED and the reported trigger in a standard approach. Since the mean was equal to the variance, a Poisson regression was used to examine the relative incidence of peanut/tree nut-induced anaphylaxis on Halloween, Christmas, and Easter, versus the rest of the year (i.e.: excluding all 3 holidays). Each holiday was a 1-day period.

**Results:** Over the 7 years of the study, 1235 cases of peanut/tree nut-induced anaphylaxis were reported, with a median age of 5.3 years (IQR, 2.5, 10.6), and 62% were males. There was a higher incidence rate ratio (IRR) for peanut/tree nut-induced anaphylaxis on Easter and Halloween [IRR 1.71 (95%CI 1.19, 2.46) and 1.66 (95%CI 1.15, 2.40), respectively]. There was no significant difference in the IRR between Christmas (IRR 0.85 (95%CI 0.49, 1.47)) and the rest of the year. On Halloween, peanut-induced anaphylaxis (IRR 1.96 (95%CI 1.21, 3.17)) doubled, compared to the non-holiday period. Anaphylaxis from tree nuts during Halloween (IRR 1.03 (95%CI 0.46, 2.31)) was as frequent as the rest of the year.

**Conclusion:** Differences in the incidence of peanut and tree nut-induced anaphylaxis during Easter and Halloween are likely due to higher risk of exposure to these culprits at these specific time points. Educational programs aiming to increase caregivers’ vigilance on inadvertent exposure to peanut/tree nuts during Easter and Halloween are required.

### #51 A survey-based evaluation of cannabis hypersensitivity in Vancouver allergy practices following legalization of recreational marijuana

#### Shun Chi Ryan Lo^1^, Mina Abbaslou^2^, Khaldon F. Abbas^2^, Deena Kobric^2^, Donald Stark^3^, Amin Kanani^3^, William Silvers^4^, Gordon Sussman^5^

##### ^1^Department of Medicine, University of British Columbia, Vancouver, BC, Canada; ^2^Gordon Sussman Clinical Research Inc., Toronto, ON Canada; ^3^Division of Allergy and Immunology, Department of Medicine, University of British Columbia, Vancouver, BC, Canada; ^4^Division of Allergy, Asthma, and Clinical Immunology, Department of Medicine, University of Colorado School of Medicine, Aurora, CO, USA; ^5^Division of Clinical Immunology and Allergy, Department of Medicine, University of Toronto, Toronto, ON, Canada

###### **Correspondence:** Shun Chi Ryan Lo

*Allergy Asthma Clin Immunol* 2020, **16(Suppl 1):**#51

**Background:** Hypersensitivity to *Cannabis sativa* and to marijuana, preparations of its flowers, have been reported but remains understudied. Symptoms range from mild to life-threatening, including anaphylaxis, generally correlating with route of exposure. An implicated allergen is *Can s 3*, a non-specific lipid transfer protein that may confer cross-reactivity with many fruits and vegetables. With legalization of recreational marijuana use in Canada in October 2018, sensitization to cannabis will likely become more prevalent through increased exposure. Here, we aim to characterize marijuana exposure and explore prevalence of symptoms attributable to cannabis hypersensitivity in a Vancouver population.

**Methods:** A self-administered, anonymous paper questionnaire adapted from previously published studies was distributed to adult patients at 2 allergy clinics in Vancouver since October 2018. Information regarding route and frequency of marijuana use, associated symptoms, and concomitant allergic conditions was obtained. The study was approved by the Canadian SHIELD Ethics Review Board, approval number 18-10-002.

**Results:** 58 of 66 distributed questionnaires were completed. 18 (31.6%) participants were male. Mean age was 38.8 years. Commonly reported routes of marijuana consumption were inhalation (64.2%), topical application (35.1%) and ingestion (31.0%). 48 (82.8%) participants have used marijuana, among which 35 (73%) reported symptoms upon exposure. Respiratory and ocular symptoms were most frequent (91.4%), followed by dermatologic (25.7%) and gastrointestinal (17.1%). 33 participants were actively using marijuana, with 25 (75.8%) reporting symptoms. 4 of 8 (50%) participants without previous marijuana consumption also reported symptoms. 21 (60%) participants with marijuana exposure, and 3 (30%) participants without, reported food allergy.

**Conclusions:** A large portion of marijuana users attribute allergic symptoms to marijuana consumption. Nevertheless, most continue to use marijuana. A higher proportion of patients with marijuana exposure reporting food allergies may be driven by cross-reactivity. Future directions include determining sensitization by validated testing.

### #52 School principals’ reports of epinephrine auto-injector accessibility and administration in Manitoba schools

#### Keely Loewen^1,2^, Nancy Ross^1^, Sandra Dalke^3^, Shauna Filuk^1^, Bev Kulbaba^1^, JoAnne St. Vincent^1^, Elinor Simons^1,2^

##### ^1^Children’s Allergy & Asthma Education Centre (CAAEC), Health Sciences Centre, Winnipeg, MB, Canada; ^2^Section of Allergy & Clinical Immunology and Children’s Hospital Research Institute of Manitoba, Department of Pediatrics & Child Health, Max Rady College of Medicine, University of Manitoba, Winnipeg, MB, Canada; ^3^ United Referral Intake Service (URIS), Winnipeg Regional Health Authority, Winnipeg, MB, Canada

###### **Correspondence:** Keely Loewen

*Allergy Asthma Clin Immunol* 2020, **16(Suppl 1):**#52

Keely Loewen and Nancy Ross are co-first authors

**Background:** Students at risk for anaphylaxis must strictly avoid their allergens and have their epinephrine auto-injectors available at all times. In Manitoba, students with life-threatening allergies are provided with an Anaphylaxis Standard Health Care Plan. Teachers and school staff receive training on recognition and management of anaphylaxis by registered nurses through the United Referral Intake Service (URIS) Program. Manitoba school divisions are creating a policy to have “back up” stock epinephrine auto-injectors available for students who have been prescribed an epinephrine auto-injector. This study examines the baseline accessibility of epinephrine auto-injectors in schools before the availability of stock epinephrine.

**Methods:** The University of Manitoba Health Research Ethics Board and participating school divisions approved the study. School principals were emailed an Invitation Letter and SurveyMonkey^®^ Link for a brief questionnaire regarding epinephrine auto-injectors in their schools during the 2018–2019 school year. We examined number of anaphylaxis episodes, epinephrine auto-injector administration, and ability to locate the auto-injectors of students experiencing anaphylaxis.

**Results:** After the first invitation, 26 of 78 invited school principals (33%) participated by the last week of June 2019. Of the principals who responded, 3.8% reported a child or children experiencing 1–2 episodes of anaphylaxis. In each case, the individuals’ own epinephrine auto-injectors were used and were not difficult to find. Preliminary data did not identify trouble locating at-risk individuals’ epinephrine auto-injectors in schools when they were needed to treat anaphylaxis.

**Conclusions:** Among participating schools, anaphylaxis at school was a rare occurrence and the individuals’ own auto-injectors were administered. We will send a second wave of questionnaires to school principals in September. We will also repeat this questionnaire after the next school year to evaluate epinephrine auto-injector use and the influence of stock epinephrine auto-injector availability.

**Acknowledgments:** We thank the participating school divisions, all school principals who completed the questionnaire and The Canadian Allergy, Asthma and Immunology Foundation (CAAIF) for funding this study.

### #53 A real world evaluation of pediatric preschool peanut oral food challenges in a private practice clinic prior to oral immunotherapy

#### Douglas P. Mack^1,2^, Mariam A. Hann^1,2^

##### ^1^Halton Pediatric Allergy, Burlington, ON, Canada; ^2^Department of Pediatrics, McMaster University, Hamilton, ON, Canada

###### **Correspondence:** Douglas P Mack

*Allergy Asthma Clin Immunol* 2020, **16(Suppl 1):**#53

**Background:** The oral food challenge, in combination with clinical history is the gold standard for diagnostic evaluation of food allergy. In clinical practice, allergists often utilize skin prick testing (SPT) and/or blood work in conjunction with clinical history to diagnose food allergy. For a number of reasons, oral challenges continue to be infrequently performed and are generally under-utilized in clinical practice. As therapies such as oral immunotherapy (OIT) for food become more commonplace, proper diagnosis of food allergy is essential.

**Methods:** A quality improvement retrospective chart review was performed involving all oral challenges to peanuts in a private practice clinic specializing in OIT between May, 2017 and May, 2019.

**Results:** Of 1980 oral challenges performed, 118 were for peanut in patients < 6 years or ages referred for OIT. Of these, 67 patients passed the oral challenge. Of those that passed, 56 had a history of prior clinical reaction. Mean age for all patients was 2.45 years. Mean peanut SPT was 7.23 mm, Peanut SSIgE was 8.15 KU/L and AraH2 IgE was 3.48, and all mean values were higher in those that failed the oral challenge. No severe reactions during oral challenge were reported with the most common symptoms being urticaria, mouth/throat itch and vomiting. Rupatadine was used in 98% of oral challenges and epinephrine was used in 16%. Comparison of outcomes was also made with patients that were not referred for OIT. Referral source was also evaluated.

**Conclusions:** In a real world study of a preschool population referred for OIT, only a minority of patients had confirmed peanut allergy, and reaction severity in this population was mild-moderate. This has significant implications as OIT guidelines are developed and commercial products are being marketed. Oral challenges should form the foundation of food allergy diagnosis, especially in patients interested in OIT.

### #54 Pre-hospital management of anaphylaxis in rural Quebec

#### Laura May Miles^1^, Sofianne Gabrielli^1^, Mohamad Ghrooda^1^, Greg Shand^1^, Ann Elaine Clarke^2^, Jocelyn Moisan^3^, Moshe Ben Shoshan^1^

##### ^1^Division of Allergy and Clinical Immunology, Department of Pediatrics, Montreal Children’s Hospital, McGill University Health Centre, Montreal, QC, Canada; ^2^Division of Rheumatology, Department of Medicine, Cumming School of Medicine, University of Calgary, Calgary, AB, Canada; ^3^Regional Medical Director of Emergency Medical Services of Outaouais, Outaouais, QC, Canada

###### **Correspondence:** Laura May Miles

*Allergy Asthma Clin Immunol* 2020, **16(Suppl 1):**#54

**Background:** Anaphylactic reactions are medical emergencies requiring immediate management by patients. Data on pre-hospital management of anaphylaxis by emergency medical services (EMS) is often limited to metropolitan centers. Management of anaphylaxis in rural settings of Quebec remains unclear. We aimed to assess anaphylaxis rate, triggers and management by EMS.

**Methods:** Over a four-year period, patients with anaphylaxis requiring EMS in the Outaouais region of Quebec were recruited as part of the Cross-Canada Anaphylaxis REgistry (C-CARE). Treating paramedics collected data on patients’ demographics, co-morbidities, triggers of anaphylaxis, and clinical presentation on a tablet based on a standardized questionnaire incorporated into a software program developed specifically for the study.

**Results:** From 2013 to 2017, 433 patients requiring EMS due to an anaphylactic reaction were recruited, of which 42.6% were male, with a median age of 40.7 years (interquartile range [IQR]: 20.1, 59.0). The percentage of anaphylaxis among all ambulance calls did not change significantly [0.06% (95%CI − 0.02%, 0.15%)] over this time interval. Among patients with anaphylaxis, 37.4% presented with a food allergen trigger. Among food allergens, the most common trigger was peanut (19.8%). Around 20% of anaphylactic reactions were reported to be triggered by drug (17.6% of which were triggered by moxifloxacin), and similarly 20.1% were caused by venom. Approximately 15% of anaphylactic reactions were caused by an unknown exposure. Prior to EMS arrival, only 33.7% patients were administered epinephrine, compared to 35.6% who were administered antihistamines. Prior to EMS arrival, 2.7% of patients were administered steroids. Almost 60% were administered epinephrine by the paramedics, 0.4% were administered antihistamines and none were administered steroids. One-fifth (20.8%) of patients were administered epinephrine both before and after EMS arrival.

**Conclusion:** Epinephrine administration by patients prior to EMS arrival occurs in less than half of the cases of anaphylaxis in rural settings of Quebec.

### #55 Improvement in disease-specific quality of life for peanut-allergic subjects continuing AR101 therapeutic dosing for an additional 28 weeks

#### Jason Ohayon^1^, Gordon Sussman^2^, Moshe Ben-Shoshan^3^, Amarjit Cheema^4^, William H. Yang^5^, Noelle M. Griffin^6^, Deborah Cebrik^6^, Andrea Vereda^7^, Jonathan Hourihane^8^, on behalf of the investigators

##### ^1^Hamilton Allergy, Hamilton, Canada; ^2^Gordon Sussman Clinical Research, Toronto, Canada; ^3^McGill University Health Center, Montreal, Canada; ^4^Cheema Research, Mississauga, Canada; ^5^Ottawa Allergy Research Corporation, University of Ottawa Medical School, Ottawa, Canada; ^6^Aimmune Therapeutics, Brisbane, CA, USA; ^7^Aimmune Therapeutics, London, UK; ^8^University College Cork, Cork, Ireland

###### **Correspondence:** Jason Ohayon

*Allergy Asthma Clin Immunol* 2020, **16(Suppl 1):**#55

**Background:** Peanut allergy is associated with poor health-related quality of life (QoL). PALISADE was a phase 3 double-blind, placebo-controlled trial that investigated the safety and efficacy of AR101, an investigational oral biologic drug for use in oral immunotherapy in peanut-allergy subjects. A subgroup of subjects continued the therapeutic dose of AR101 (300 mg/day) in an open-label follow-on study (ARC004) for 28 weeks. Here we report on the changes in QoL in these subjects.

**Methods:** Subjects 8–17-years-old (self-report) and parents/caregivers of subjects 4–17-years-old (proxy-report) completed an age-appropriate Food Allergy Quality of Life Questionnaire (FAQLQ) and Food Allergy Independent Measure (FAIM) at PALISADE screening, exit and at ARC004 exit. Total and domain scores were calculated for all time points for the overall population and stratified by responder status, systemic allergic reactions, or adrenaline use during the study period. Changes in scores (screening to exit) were calculated to determine if they exceeded the developer-referenced minimally important difference (MID) ≥ 0.5.

**Results:** 110 PALISADE subjects 4–17-years-old received 300 mg/day AR101 in ARC004; 68 subjects and 93 parents/caregivers completed FAQLQ and FAIM. Self-reported QoL from screening to exit in total scores for FAQLQ and FAIM improved and exceeded the MID (FAQLQ: PALISADE 4.48 vs. ARC004 3.77; FAIM: PALISADE 3.70 vs. ARC004 3.10). Proxy-reported QoL from screening to exit improved but did not exceed the MID (FAQLQ: PALISADE 4.08 vs. ARC004 3.74; FAIM: PALISADE 3.81 vs. 3.40). Changes in self- and proxy-reported QoL scores were similar regardless of responder status, systemic allergic reactions, or epinephrine use.

**Conclusions:** AR101-treated subjects continuing the therapeutic dose (300 mg/day) for an additional 28 weeks reported improved FAQLQ and FAIM scores. Gradual improvements over time may reflect the need for adjustment to their desensitization status. This report supplies the first evidence in a blinded trial of positive changes in QoL for subjects with food allergy.

### #56 Perceived parental mental health impact of food allergy

#### Elissa M. Abrams^1,2,3,4^, Leslie Roos^5^, Elinor Simons^1,6^, Kim Hurst^2,7^, Jennifer L.P. Protudjer^1,2,8,9,10^

##### ^1^Department of Medicine, University of Manitoba, Winnipeg, MB, Canada; ^2^Children’s Hospital Research Institute of Manitoba, Winnipeg, MB, Canada; ^3^Meadowood Medical Centre, Winnipeg, Canada; ^4^Department of Pediatrics, Division of Allergy and Immunology, University of British Columbia, Vancouver, BC, Canada; ^5^Deparment of Psychology, University of Manitoba, Winnipeg, Winnipeg, MB, Canada; ^6^Department of Pediatrics, Section of Allergy and Clinical Immunology, University of Manitoba, Winnipeg, MB, Canada; ^7^Department of Health Sciences, Queen’s University, Kingston, ON, Canada; ^8^George and Fay Yee Centre for Healthcare Innovation, Winnipeg, MB, Canada; ^9^Department of Food and Human Nutritional Sciences, University of Manitoba, Winnipeg, MB, Canada; ^9^Institute of Environmental Medicine, Karolinska Institutet, Stockholm, Sweden

###### **Correspondence:** Jennifer L.P. Protudjer

*Allergy Asthma Clin Immunol* 2020, **16(Suppl 1):**#56

**Background:** Approximately 8% of children have food allergies, which impacts family life. Yet, parents’ coping strategies for this disease burden are incompletely understood. We aimed to describe the perceptions of allergy-related mental health issues in families with food allergy.

**Methods:** This is an ongoing qualitative interview study of parents of children with paediatric allergist-diagnosed food allergy, who were recruited in allergy clinics and education centres in Winnipeg. Interviews were transcribed verbatim. We used content analysis from which we identified preliminary overarching themes. The University of Manitoba Health Research Ethics Board approved this study (HS22242 (H2018-408)).

**Results:** To date, we have interviewed 13 mothers, with children aged < 12 months to 13 years. Perceived mental health impacts varied by type and number of food allergies, and time lapse since diagnosis. For parents whose children had a single food allergy, “***Accommodation and Adaptation***” was described, whereas most parents of children with multiple food allergies reported negative impacts on their mental health. As captured in the theme, “***Pragmatism and Isolation***”, many spoke of being “*depressed*” and “*terrified*” about leaving their children in the care of others who were perceived as being only minimally equipped to handle food allergy. Parents felt “*overwhelmed and alone*,” and engaged in negative ways of coping. For parents who lacked support from their extended family and/or daycare/school, this impact was particularly strong. Time lapse since diagnosis also impacted perceived mental health. “***Fear for today, fear for the future***” was commonly described by parents whose children were recently diagnosed, whereas the theme, “***Food allergy management has become our normal***” was identified amongst parents whose children had been living with the condition for some time.

**Conclusion:** Food allergy has varying impacts on parental mental health. Most negatively affected are those whose children have multiple food allergies, and/or who have recently been diagnosed.

### #57 Dairy intake amongst Manitoba adolescents with and without food allergy by Jennifer Protudjer

#### Jennifer L.P. Protudjer^1,2,3,4,5^, Anita L. Kozyrskyj^6^, Allan B. Becker^1,2^

##### ^1^Department of Pediatrics and Child Health, University of Manitoba, Winnipeg, MB, Canada; ^2^Children’s Hospital Research Institute of Manitoba, Winnipeg, MB, Canada; ^3^George and Fay Yee Centre for Healthcare Innovation, Winnipeg, MB, Canada; ^4^Department of Food and Human Nutritional Sciences, University of Manitoba, Winnipeg, MB, Canada; ^5^Institute of Environmental Medicine, Karolinska Institutet, Stockholm, Sweden; ^6^Department of Pediatrics, University of Alberta, Edmonton, AB, Canada

###### **Correspondence:** Jennifer L. P. Protudje

*Allergy Asthma Clin Immunol* 2020, **16(Suppl 1):**#57

**Background:** Food allergy may impact diet quality and nutrient intake. The aim of this study was to examine intake patterns of calcium-rich foods and supplements, and dairy quality amongst adolescents with or without a history of food allergy.

**Methods:** We examined intake patterns of calcium-rich products amongst Manitoba adolescents by food allergy status, using data from a cohort born in 1995 and followed to adolescence. At ages 7–8 (childhood) and 12–14 (adolescence) years, parents reported if their child had ever had food allergy. Adolescents completed food frequency questionnaires, which queried calcium-rich foods calcium-fortified orange juice and multivitamin/mineral supplements. Intake patterns were defined as 1+ weekly, vs. < 1 weekly, except calcium-fortified orange juice, which was categorized as no/yes. Dairy quality scores were classified based on the Youth Health Eating Index, with low-fat milk and yoghurt consumption scored twice as high as high-fat, high-sugar dairy. Logistic regression was used, with adjustments for confounders. This study was approved by The University of Manitoba Health Research Ethics Board (HS14742 (H2002:078)).

**Results:** Overall, 472 adolescents were included, including 60 (13%) with food allergy ever. Compared to adolescents without food allergy, adolescents with a history of food allergy were significantly less likely to consume milk 1+ weekly (OR 0.39; 95%I 0.20–0.77). In contrast, intake patterns of other calcium-rich foods and supplements were similar between the groups (all p > 0.05). Mean dairy quality was poor (4.1/10). Although dairy quality scores tended to be slightly higher amongst boys than girls (p = 0.08), it did not differ by food allergy status in childhood, in adolescence or by timing.

**Conclusion:** Manitoba adolescents with a history of food allergy consume less milk, but similar amounts of other calcium-rich foods, compared to their non-food allergic peers. Whereas dairy quality was poor amongst all adolescents, it did not differ by dairy quality scores.

### #58 Specific IgE antibody levels during and after food-induced anaphylaxis

#### S. Rehimini^1^, S. Gabrielli^1^, A. Langlois^1^ A. Clarke^2^, S. De Schryver^1^, G. Shand^1^ and M. Ben-Shoshan^1^

##### ^1^Department of Pediatrics, Division of Pediatric Allergy and Clinical Immunology, McGill University, Montreal, QC, Canada; ^2^Department of Medicine, Cumming School of Medicine, University of Calgary, Calgary, AB, Canada

###### **Correspondence:** S. Rehimini

*Allergy Asthma Clin Immunol* 2020, **16(Suppl 1):**#58

**Background:** Little is known about the dynamics of specific IgE antibody (sIgE) levels during and after an allergic reaction. We evaluated the differences in food sIgE levels during and after food-induced anaphylaxis.

**Methods:** Patients presenting to the Emergency Department (ED) of the Montreal Children’s Hospital with food-induced anaphylaxis were recruited for the Cross-Canada Anaphylaxis Registry (C-CARE) between 2013 and 2019. sIgE levels (Phadia ImmunoCAP) of the suspected culprit allergen and of other related and unrelated foods, in addition to tryptase levels, were drawn within 2 h of presentation and at least 24 h later. We compared sIgE (and tryptase) levels during and after the reaction with the paired Wilcoxon test. A multivariable linear regression model was used to predict factors associated with difference in sIgE levels of the reported culprit allergen during and after the reaction.

**Results:** Among 50 consenting pediatric cases, sIgE levels of the reported culprit allergen after anaphylaxis were higher than during the reaction, yielding a mean difference of 8.41 kUa/L (p = 7.87 × 10^−6^). The median time interval for sIgE differences was 4 weeks (IQR = 5). Among the 34 patients who had their sIgE levels of unrelated foods measured, the difference was not substantial (2.15 kUa/L, p = 0.473). In 46 patients, tryptase levels during and after anaphylaxis were also measured. All had decreased levels after the reaction consistent with the definition of anaphylaxis with a total mean difference of 7.03 μg/L (p = 2.59 × 10^−9^). Older children (0.067 (95%CI 0.00094, 0.13)) and children with higher tryptase level difference (0.83 (95%CI 0.14, 1.52)) were found to be more likely to have higher differences in sIgE levels, while adjusting for sex, and difference in sIgE levels of unrelated foods.

**Conclusion:** The results suggest that changes in sIgE levels may contribute to identifying the culprit allergen when history is unclear.

The study was approved by McGill University Health Center Research Ethics Board. REB # 12-084 PED.

### #59 Extended daily dosing of AR101 for peanut allergy results in higher tolerated doses and continued immunomodulation in subjects aged 4–17 years

#### Gordon Sussman^1^, Moshe Ben-Shoshan^2^, Amarjit Cheema^3^, Jason Ohayon^4^, William H. Yang^5^, Katharina Blümchen^6^, Noelle M. Griffin^7^, Deborah Cebrik^7^, M. Dolores Ibáñez^8^, Antonella Muraro^9^, Stacie M. Jones^10^, George du Toit^11^, Jonathan O’B. Hourihane^l2^

##### ^1^Gordon Sussman Clinical Research, Toronto, Canada; ^2^McGill University Health Center, Montreal, Canada; ^3^Cheema Research, Mississauga, Canada; ^4^Hamilton Allergy, Hamilton, Canada; ^5^Ottawa Allergy Research Corporation, University of Ottawa Medical School, Ottawa, Canada; ^6^University Hospital Frankfurt, Frankfurt, Germany; ^7^Aimmune Therapeutics, Brisbane, CA, USA; ^8^H. Infantil Universitario Niño Jesús, and ARADyAL, Madrid, Spain; ^9^Food Allergy Referral Centre Veneto Region, Department of Woman and Child Health, Padua University Hospital, Padua, Italy; ^10^University of Arkansas for Medical Sciences and Arkansas Children’s Hospital, Little Rock, AR, USA; ^11^Guy’s and St. Thomas’ NHS Foundation Trust, London, UK; ^12^University College Cork, Cork, Ireland

###### **Correspondence:** Gordon Sussman

*Allergy Asthma Clin Immunol* 2020, **16(Suppl 1):**#59

**Background:** In PALISADE, a phase 3 study of AR101 in North America and Europe, immunological changes suggested immunomodulation of peanut allergy during the first year of treatment, including 6 months of dose escalation and 6 months at the therapeutic dose (300 mg/day). Efficacy, safety, and immunological changes after 28 additional weeks of 300 mg/day in subjects 4–17-years-old is reported.

**Method:** AR101-treated PALISADE completers tolerating ≥ 300 mg peanut protein at double-blind, placebo-controlled food challenge (DBPCFC) entered the follow-on study ARC004. A subset (including Canadian subjects) continued 300 mg/day AR101 for 28 weeks and completed a DBPCFC with an additional 2000 mg (4043 mg cumulative) challenge dose. Peanut skin-prick test (SPT) and peanut-specific IgE (psIgE) measurements (reported as median [Q1, Q3]) were compared (PALISADE vs. ARC004).

**Results:** 110/285 AR101-treated subjects aged 4–17 years continued 300 mg/day AR101; the remainder were assigned to other dosing arms (not reported here). 94% of subjects completed the additional 6-month dosing period and exit DBPCFC. Two subjects discontinued AR101 due to adverse events (AEs). The proportion of subjects reporting related AEs were similar (PALISADE 45% vs. ARC004 43%) but the number of events decreased (739 vs. 371 events). Median tolerated dose at ARC004 DBPCFC was 1000 mg (1000, 2000) (n = 104). Of subjects who tolerated < 1000 mg at PALISADE exit (n = 37), 70% tolerated a higher challenge dose at ARC004 exit. 49% of subjects tolerated the 2000 mg challenge dose. Immunological changes continued (PALISADE vs. ARC004 exits): SPT wheal diameter, 7.5 mm (5.5, 10.0) vs. 7.0 mm (5.0, 9.0); psIgE, 59.3 (19.8, 245.5) vs. 40.5 (15.8, 90.0); psIgE/IgG4, 12.5 (2.3, 29.9) vs. 5.1 (1.2, 16.0).

**Conclusion:** AR101 showed improved tolerability after 28 additional weeks of 300 mg/day AR101, with AEs decreasing over time. Higher tolerated doses were matched by favourable immunological changes, suggestive of ongoing immunomodulation with therapeutic dose continuation, reinforcing the rationale for continued daily AR101 dosing after 1 year.

### #60 Evaluation of a Canadian oral immunotherapy community programme

#### Eunice Tunggal^1^, Joel Liem^1,2^

##### ^1^Windsor Asthma Allergy Education Centre, Windsor, ON, Canada; ^2^Schulich School of Medicine and Dentistry, Western University, London, ON, Canada

###### **Correspondence:** Eunice Tunggal

*Allergy Asthma Clin Immunol* 2020, **16(Suppl 1):**#60

**Background:** Although oral immunotherapy (OIT) has become popular in other countries, its use by Canadian community allergists has only begun over the past several years. Concerns exist regarding safety and evidence of effectiveness. We report outcomes at Windsor Allergy Asthma Clinic.

**Methods:** A chart review of patients who began OIT in May 2017 until May 2019 examined 3 parts of OIT: start-up, build-up and maintenance phases (top dose = 700–1000 mg protein). Adverse reactions and anaphylaxis were recorded and graded by the World Allergy Organization Subcutaneous Immunotherapy Systemic Reaction Grading System. Analysis was conducted on patients divided into 3 age groups (< 6, 6–12, > 12 years). Risk factors for adverse events were examined.

**Results:** 64 children (65% male) began OIT for peanut (59), cashew/pistachio (3), cashew (1), or wheat (1). On start-up day, 14 (22%) patients had adverse reactions (grade 1/2). During build-up, 53 (83%) patients reported an adverse reaction at least once (grade 1/2); 10 (15%) used EpiPen for anaphylaxis. Patients > 12 trended towards increased risk of anaphylaxis compared to < 6 (OR = 5.1, P = 0.06) and 6–12 (OR = 4.5, P = 0.08) year-old age groups. Later ingestion times (past 12PM) and exercise within 3 h were associated with 63% and 82% of anaphylaxes, respectively. 36% of all anaphylaxis occurred on weekends and school holidays. At maintenance, 7 patients reported reaction; EpiPen was given once. Overall, 63/64 patients continue treatment; 1 withdrew for reasons unrelated to anaphylaxis or adverse reaction. Currently, 21 patients are in build-up, with 47 in maintenance.

**Conclusions:** OIT in Canadian communities is feasible only if the allergist is aware of reaction risks and can work with patients to persist. Risk factors of older age, ingestion times, and exercise must be cautioned. Quality of life for these patients and families should be examined, as 98% of OIT patients in this community want and continue with treatment.

### #61 Anaphylaxis to hemp seed ingestion: a case series

#### Dennis Wong^1^, Khaldon F. Abbas^2^, Gordon L. Sussman^1,2^

##### ^1^Department of Medicine, University of Toronto, Toronto, ON, Canada; ^2^Gordon Sussman Clinical Research Inc, Toronto, ON, Canada

###### **Correspondence:** Dennis Wong

*Allergy Asthma Clin Immunol* 2020, **16(Suppl 1):**#61

**Background:***Cannabis sativa* is an annual herbaceous flowering plant in the family of Cannabaceae. Cultivars with higher concentration of the psychoactive component tetrahydrocannabinol (THC) are generally used for medicinal purposes or consumed as recreational drug known as marijuana. Hemp is a variety of *Cannabis sativa* that contain lower THC concentration, and hemp seed has become a popular food ingredient due to its nutritious value. There are few reports to date on anaphylaxis to hemp seed, and cannabis is a new allergen that is not well-recognized. We, therefore, investigated the clinical features of seven patients with anaphylaxis to hemp seed ingestion.

**Methods:** Seven patients who presented to an ambulatory allergy clinic for hemp seed allergy with clinical symptoms fitting the diagnostic criteria of anaphylaxis were evaluated. Skin prick test with a cannabis extract was performed.

**Results:** The mean age of the patients was 33 (range 26–40) years, and there were 4 females and 3 males. All patients had positive skin prick test to cannabis. 6 patients have previously smoked cannabis and had no known prior exposure to hemp seed. One patient has never been exposed cannabis before having anaphylaxis to hemp seed ingestion. The symptoms involved multiple systems, including urticaria, angioedema, dyspnea, syncope and, most commonly, abdominal cramps. Of the 6 patients who had previous exposure to cannabis, none had anaphylaxis to smoking cannabis.

**Conclusions:** We present one of the first case series of patients with anaphylaxis to hemp seed ingestion. There is evidence showing that smoking cannabis could have sensitized our patients to hemp seed. With legalization of cannabis in Canada, the prevalence of cannabis allergy will likely be on the rise. Patients exposed to cannabis should be made aware of the potential risks as hemp seed is added to a variety of foods due to its increasing popularity.

### #62 Early skin prick testing infants with atopic dermatitis leads to sensitization of food allergens

#### Vince Wu^1^, Sydney A. Scheffler^1^, Wardha Wardha^1^, Tarin T. Moni^1,2^, Kristiina E. Frechette^1,2^, Jason A. Ohayon MD^1,3^

##### ^1^Hamilton Allergy, Hamilton, ON, Canada; ^2^McMaster University, Hamilton, ON, Canada; ^3^Department of Pediatrics, McMaster University, Hamilton, ON, Canada

###### **Correspondence:** Vince Wu

*Allergy Asthma Clin Immunol* 2020, **16(Suppl 1):**#62

**Background:** Skin prick testing (SPT) is commonly performed to assess for allergen sensitization, but SPT infants with atopic dermatitis who have an impaired skin barrier may sensitize them to tested allergens.

**Methods:** A retrospective chart review was conducted at a community clinic identifying infants with atopic dermatitis (AD) who were evaluated for allergic sensitization via skin testing (ST). AD infants who were initially positive for allergies and were re-evaluated with repeat ST were selected. Allergy ST assessments were directed towards foods including milk (1:10 w/v), egg white (1:100 w/v), egg yolk (1:100 w/v), soy (1:10 w/v), wheat (1:10 w/v), tree nut mix (1:10 w/v), and peanuts (1:10 w/v). Infants with a wheal size 3 mm or greater, than negative control were considered sensitized to these foods with advice on avoidance until formal oral challenge was arranged to confirm tolerance or allergy. Results of ST were compared at separate visits to each food.

**Results:** The wheal size of 30% (7/23) of milk, 28% (7/25) of egg white, 25% (6/24) of egg yolk, 0% (0/9) of soy, 10% (1/10) of wheat, 37.5% (3/8) of tree nut mix, and 31.3% (5/16) of peanut skin tests increased by 3 mm or more in the most recent skin test compared to initial testing. Seventy-one percent of milk (5/7), 85.7% of egg white (6/7), 83.3% of egg yolk (5/6), 0% of soy (0/9), 100% of wheat (1/1), 66.7% tree nuts (2/3), and 60% of peanut (3/5) of these infants’ initial skin test had a wheal size of 0 mm.

**Conclusions:** The dual exposure to allergen hypothesis states that sensitization to foods is a result of cutaneous exposure to potential allergens. Consequently, SPT infants with AD may increase risk for sensitization to tested allergens due to increased cutaneous exposure through impaired skin barrier.

### #63 Milk immunotherapy induces casein-specific, CD137+FOXP3+ regulatory T cells during oral-induced tolerance among children

#### Lei Li^1^, Yi Zhang^1,2,^ Sabrina Bartolucci^1^, Nils Parvey^1^, Wei Zhao^1^, Duncan Lejtenyi^1^, Bahar Torabi^1^, Danbing Ke^1^, Bruce Mazer^1^, Ciriaco A. Piccirillo^1^

##### ^1^Programs in Infectious Disease and Immunity in Global Health and Translational Research in Respiratory Diseases, Research Institute of the McGill University Health Centre, 1001 Boulevard Décarie, Montréal, QC, H4A 3J1; ^2^Department of Otorhinolaryngology Head and Neck Surgery, Beijing Chaoyang Hospital, Capital Medical University, Beijing, 100020, China

###### **Correspondence:** Lei Li

*Allergy Asthma Clin Immunol* 2020, **16(Suppl 1):**#63

**Background:** The mechanisms of desensitization with cow’s milk oral immunotherapy (OIT) are not well understood. Previous work showed that regulatory T cells (Tregs) are key inhibitors of allergic immune responses, including Th2 effectors cells (Teffs), and promote tolerance development. However, the contribution of antigen-specific Tregs, relative to polyclonal Tregs, in the induction of oral tolerance to cow’s milk protein (CMP) in humans remains unclear. We sought to detect induction of CMP-specific Tregs, relative to CMP-specific Teffs in a cohort of subjects undergoing OIT.

**Methods:** Thirteen children with confirmed CMP allergy underwent OIT with escalating doses of milk up to 300 ml (maintenance) and were followed for up to 12 months post-maintenance. For each patient, PBMCs were obtained at baseline and specific milestones during dose escalation and maintenance. Casein-specific T cells were expanded from PBMCs by culturing for 10 days with casein. Casein-specific CD4+Teffs (Cs-Teffs, FoxP3-Helios- expressing CD154) or Tregs (Cs-Tregs, Foxp3+Helios+ expressing CD137) were quantified in proliferating (CTVlow) or non-proliferating (CTVhigh) culture fractions, and characterized by flow cytometry. The study was approved by Research Institute of the McGill University Health Centre’s Ethics Board, approval number PED-12-090.

**Results:** Cs-Tregs were found in proliferating cultures in OIT subjects. The proportion of Cs-Tregs increased significantly with escalation of milk doses during OIT. There was no change in Cs-Tregs in non-proliferating culture. There was also no change in Tregs against a control antigen, tetanus toxoid. Induction of Cs-Tregs correlated with reduction in frequency of Cs-Teffs and in vitro Th2 cytokine production. An increased ratio of Cs-Tregs to Cs-Teffs in proliferating cultures, was associated with developing tolerance among OIT subjects.

**Conclusion:** OIT to CMP induces highly suppressive Cs-Tregs, compared to pretreatment levels, and Tregs levels increased with increasing exposure to CMP. CMP-specific Tregs may inhibit CMP-specific Teffs in children receiving OIT.

### #64 Serological analysis of milk oral immunotherapy participants

#### Wei W. Zhao^1^, Casey Cohen^1^, Duncan Lejtenyi^2^, Danbing Ke^2^, Moshe Ben-Shoshan^3^, Bruce D. Mazer^3^

##### ^1^The Research Institute of the McGill University Health Centre, Meakins-Christie Laboratories, Division of Experimental Medicine, Department of Medicine, Montreal, QC, Canada; ^2^The Research Institute of the McGill University Health Centre, Meakins-Christie Laboratories, Division of Pediatric Allergy and Clinical Immunology, Department of Pediatrics, Montreal Children’s Hospital, Montreal, QC, Canada; ^3^Montreal Children’s Hospital, Division of Pediatric Allergy and Clinical Immunology, Department of Pediatrics, Montreal, QC, Canada

###### **Correspondence:** Wei W. Zhao

*Allergy Asthma Clin Immunol* 2020, **16(Suppl 1):**#64

**Background:** Cow’s milk allergy (CMA) is an inappropriate immune response against cow’s milk protein (CMP) and is the leading cause of severe anaphylaxis in children. Our laboratory is currently leading the first Canadian randomized controlled trial of milk oral immunotherapy (OIT). The goal is to enable children with CMA to tolerate ingestion of cow’s milk without developing allergy symptoms. Past work has shown that OIT alters the serum CMP-specific immunoglobulin E (sIgE) and sIgG4 levels. However, we observed a high variability in the levels of sIgE and sIgG4 over the course of milk OIT. We aim to determine clinical parameters that affect sIgE and sIgG4 level in milk OIT participants.

**Methods:** Children with confirmed CMA by history, skin prick test, and oral food challenge were studied. Serum was collected at specific time points during the escalation phase which ended at 200 ml of cow’s milk then followed for 12 months while consuming dairy. We used indirect ELISA to measure the level of serum IgE and IgG4 specific for CMPs: α-lactalbumin, β-lactoglobulin, and casein. This study was approved by the Facility Animal Care Committee of the Research Institute of the McGill University Heath Centre, approval #PED-12-090.

**Results:** Overall, the level of sIgE in milk OIT participants decreased while sIgG4 increased. Current results indicate a difference in the levels of sIgE and sIgG4 between male and female participants. Female participants have higher sIgE and lower CMP-specific sIgG4 than male participants. Correlations with clinical outcomes and adverse reaction profile were performed to address sex differences in outcome following OIT therapy.

**Conclusion:** Our current results indicate that sex difference may contribute to the high variability in levels of sIgE and sIgG4. Identification of potential factors that contribute to varied level of Ig can help us understand the immune response during OIT and food allergy.

## Immunology

### #65 Distribution and phenotype of murine regulatory B cells in HDM-driven allergic airway disease

#### Eisha A. Ahmed^1^, Shao Tao^1^, Nicholas Vonniessen^1^, Bruce D. Mazer^1,2^

##### ^1^Research Institute of McGill University Health Centre, Montreal, QC, Canada; ^2^Division of Allergy and Immunology, Department of Pediatrics, Montreal Children’s Hospital, McGill University Health Centre, Montreal, QC, Canada

###### **Correspondence:** Eisha A. Ahmed

*Allergy Asthma Clin Immunol* 2020, **16(Suppl 1):**#65

**Background:** Regulatory B cells (Bregs) play an important role in the suppression of inflammatory responses and have been implicated in the resolution of inflammation in allergic airway disease (AAD). The distribution and systemic changes in Breg populations in the context of AAD remain unclear. Here, we sought to evaluate the systemic frequency and dynamics of multiple Breg subsets prior to, and during the resolution of AAD.

**Methods:** IL-10 reporter mice were sensitized and challenged using house dust mite (HDM) extract to induce AAD protocol. We evaluated the frequency and phenotype of B cells and Bregs in the bone marrow, lungs, spleens, peripheral blood, peritoneal cavity, pleural cavity, and livers of naive mice, and sensitized mice at multiple time points following the final HDM challenge. Cell phenotypes were evaluated via flow cytometry, and correlations of Breg populations post-challenge with the resolution of airway eosinophilia were assessed. This study was approved by the Facility Animal Care Committee of the Research Institute of the McGill University Heath Centre (RI-MUHC), protocol #6011.

**Results:** Preliminary results demonstrate substantial IL-10 expressing Breg populations in peritoneal cavity, pleural cavity, and the bone marrow of naive mice, which predominantly consist of cells from the B1 and CD138+ plasma cell subsets. Breg frequency increases in the lungs of allergic mice 48 h after the last HDM challenge. We also analyzed the systemic dynamics of Breg populations over the course of the natural resolution of lung inflammation is mice.

**Conclusions:** Our results show the distribution and changes in Breg populations in mice following the induction and resolution of AAD. Future work will evaluate the functional capacity of different Breg subsets to suppress airway inflammation, and correlates in human asthma.

### #66 Clinical predictors of primary immune deficiencies: a systematic review

#### Anna Branch^1,2,3^, Bhavi Modi^3^, Kyla Hildebrand^2,3^, Stuart Turvey^2,3^, Catherine Biggs^2,3^

##### ^1^Western University, London, ON, Canada; ^2^Department of Pediatrics, University of British Columbia, Vancouver, BC, Canada; ^3^BC Children’s Hospital Research Institute, Vancouver, BC, Canada

###### **Correspondence:** Anna BranchAnna Branch

*Allergy Asthma Clin Immunol* 2020, **16(Suppl 1):**#66

**Background:** Primary immunodeficiencies (PIDs) are a group of genetic disorders that affect the development and/or function of the immune system, predisposing patients to infection, cancer, autoimmunity, and inflammation. Early diagnosis is key to prevent severe, life-threatening infections and end-organ damage. Clinical tools are needed to aid clinicians in identifying patients who may have an underlying PID. This work performs a systematic review of the literature to identify features shown to distinguish PID patients from non-PID controls.

**Methods:** We searched PUBMED and WEB OF SCIENCE databases for studies describing clinical features predictive of or associated with PIDs. The results were screened and excluded if they were studying disorders other than PIDs, the sample size was < 20, they lacked clinical information, or they were case reports or review articles. Studies were included only if they had quantifiable data comparing human PID cases to non-PID controls.

**Results:** 5485 articles were identified, 1060 abstracts screened, and 8 articles were included in the final analysis. The following features were found to be predictive of PID: absolute lymphopenia or CD3^+^/CD4^+^, CD19^+^ or CD16^+^/CD56^+^ subsets below the 10th percentile, hypogammaglobulinemia, neutropenia, immune thrombocytopenia, splenomegaly, enteroviral or respiratory tract infections, serious bacterial, persistent fungal, or recurrent deep-seated infections, chronic diarrhea, failure to thrive/growth retardation, family history of PID, parental consanguinity, sibling death, need for IV or prolonged courses of antibiotics, immunodeficiency related score > 6 (a clinical scoring system of immunodeficiency-related conditions), history of hospitalization, recurrent viral infections or atopy in adulthood, autoimmunity in childhood, and adolescent presentation of recurrent/frequent infections.

**Conclusion:** Combinations of clinical features can be used to detect PID patients, and this work identifies features that have evidence supporting their use. Future work will aim to integrate these features into a clinical scoring tool that will be validated in a patient cohort at BC Children’s Hospital.

### #67 Injection site and infusion parameters of patients with primary immunodeficiency diseases initiated on immune globulin subcutaneous (human) 20% in a patient program

#### Lisa M. Meckley^1^, Yanyu Wu^1^, Spiros Tzivelekis^1^, Ahmed El-Zoeiby^2^, André Gladiator^3^

##### ^1^Global Outcomes Research and Epidemiology, The Takeda group of companies, Cambridge, MA, USA; ^2^Medical Affairs, Takeda Canada Inc., Toronto, ON, Canada; ^3^Global Medical Affairs, The Takeda group of companies, Zug, Switzerland

###### **Correspondence:** Ahmed El-Zoeiby

*Allergy Asthma Clin Immunol* 2020, **16(Suppl 1):**#67

**Background:** CUVITRU^®^ (immune globulin subcutaneous [human]; Ig20Gly) is a ready-to-use 20% liquid subcutaneous immunoglobulin (SCIG) formulation approved as antibody replacement therapy for primary immunodeficiency diseases (PID) in patients aged ≥ 2 years in the US and Canada. The HelloCUVITRU Program (HCUVP) provides 4 in-home infusions of Ig20Gly in the US to eligible patients with PID, at no cost to patients or their health insurance.

**Methods:** A retrospective, descriptive analysis of demographics, clinical characteristics, and infusion parameters of patients enrolled in the HCUVP was performed. Eligible patients had PID, were ≥ 2 years old, able to receive Ig20Gly at home, and had no history of Ig20Gly use. Patients whose first Ig20Gly infusion occurred between January 1, 2017 and September 1, 2017 were included in the analysis. Because data were anonymized in compliance with CFR 46.101(b)(4), this study was exempt from institutional review board approval.

**Results:** A total of 817 patients completed all 4 infusions (N = 817); 598 (73%) were female and mean age was 50 (range: 2–89) years. Of immunoglobulin-experienced patients with data on previous treatment, 268 (64%) received intravenously administered immunoglobulin and 152 (36%) received SCIG. By visit 4 (final), median (interquartile range) infusion rate/site was 47 (43–53) mL/h, infusion volume/site was 40 (30–50) mL/site, infusion duration was 50 (37–60) minutes, and dose was 0.2 (0.1–0.2) g/kg. During visit 4, the most common infusion area was the abdomen (upper: n = 359, 44%; lower: n = 302, 37%), followed by thigh (n = 221, 27%) and upper arm (n = 18, 2%), with most patients (n = 472, 58%) using 2 injection sites within each body area. Two-thirds (n = 544, 67%) of patients used 9 mm needles; patients also used 12 mm (n = 234; 29%) and 6 mm (n = 32; 4%) needles.

**Conclusions:** This analysis described clinical and infusion characteristics of patients with PID who initiated Ig20Gly outside a clinical trial

**Funding:** This study was funded by Baxalta US Inc., a member of the Takeda group of companies, Lexington, MA, USA.

### #68 Frequency and variant spectrum in 227 patients with molecular diagnosis of hemophagocytic lymphohistiocytosis

#### Vanessa Gadoury-Levesque^1^, Lei Dong^2^, Rui Su^2^, Jianjun Chen^2^, Kimberly Risma^1^, Rebecca Marsh^3^, Miao Sun^4^

##### ^1^Division of Allergy and Immunology, Cincinnati Children’s Hospital Medical Center, University of Cincinnati, Cincinnati, OH, USA; ^2^Department of Systems Biology, Beckman Research Institute of City of Hope, Monrovia, CA, USA; ^3^Division of Bone Marrow Transplant and Immune Deficiency, Cincinnati Children’s Hospital Medical Center, University of Cincinnati, Cincinnati, OH, USA; ^4^Division of Human Genetics, Cincinnati Children’s Hospital Medical Center, University of Cincinnati, Cincinnati, OH, USA

###### **Correspondence:** Vanessa Gadoury-Levesque

*Allergy Asthma Clin Immunol* 2020, **16(Suppl 1):**#68

**Background:** Hemophagocytic Lymphohistiocytosis (HLH) is a severe life-threatening condition characterized by excessive immune activation associated with heritable defects in multiple genes. At Cincinnati Children’s Hospital Medical Center, a panel of 15 HLH-associated genes is evaluated by next generation sequencing, which includes *AP3B1*, *BLOC1S6*, *CD27*, *GATA2*, *ITK*, *LYST*, *MAGT1*, *PRF1*, *RAB27A*, *SH2D1A*, *SLC7A7*, *STX11*, *STXBP2*, *UNC13D* and *XIAP.* The current study explored the pathogenic genetic variant distribution in HLH observed in a large tertiary clinical referral laboratory.

**Methods:** The results of HLH panels and X-linked single gene sequencing were reviewed between September 2013 and June 2018.

**Results:** A total of 1892 patients results were reviewed, and a causal genetic finding was observed in 227 (12.0%). Patients with mutations in Familial HLH (FHL types 2–5) genes tended to be diagnosed younger when compared to other genes (median age: 0.75 years [0.1–62.8] vs. 4.6 years [0.2–53.8]). Of the 227 genetically defined cases, mutations in the *PRF1* gene were the most frequent (n = 67; 29.5%). However, mutations in genes associated with degranulation defects were more common than previously noted– *STXBP2* (n = 45; 19.8%), *UNC13D* (n = 43; 18.9%), *RAB27A* (n = 17; 7.5%), *LYST* (n = 11; 4.8%) and *STX11* (n = 6; 2.6%). X-linked disorders accounted for a smaller group-*XIAP* (n = 20; 8.8%) *SH2D1A* (n = 10; 4.4%) and *MAGT1* (n = 5; 2.2%). Lysinuric protein intolerance gene *SLC7A7* (n = 3; 1.3%) mutations were least commonly identified in patients.

**Conclusion:** These results describe the largest cohort of genetic variants associated with suspected HLH in North America. Although only 12% of patients were identified with a genetic diagnosis by this diagnostic approach, the gene panel identified known pathogenic and novel variants in 15 HLH-associated genes. As a group, patients with degranulation gene defects represented the majority of cases, likely reflecting the availability of rapid immunologic screening prior to genetic testing.

### #69 MASTER: MASTtocytosis to study Evolution Registry

#### Mahdi Hassan^1,2^, Elena Netchiporouk^1^, Sofianne Gabrielli^2^, Greg Shand^2^, Elinor Simons^3^, Barbara Miedzybrodzki^2^, Fatemeh Jafarian^2^, Audrey Lovet^2^, Shawna Lechner-Rumpel^4^, Sharon Baum^5^, Shoshana Greenberger^5^, Moshe Ben-Shoshan^2^

##### ^1^Division of Dermatology, McGill University Health Centre, Montreal General Hospital, QC, Canada; ^2^Department of Pediatrics, Division of Allergy and Clinical Immunology and Dermatology, Department of Pediatrics, Montreal Children’s Hospital, Montreal, QC, Canada; ^3^Section of Allergy & Clinical Immunology, Departments of Pediatrics & Child Health, University of Manitoba, Canada; ^4^Mastocytosis Society Canada, Regina, Saskatchewan, Canada; ^5^Dermatology Department, Sheba Medical Center, Ramat Gan, Israel

###### **Correspondence:** Mahdi Hassan

*Allergy Asthma Clin Immunol* 2020, **16(Suppl 1):**#69

**Background:** Mastocytosis refers to a group of myeloproliferative disorders characterized by excessive proliferation and accumulation of mast cells in tissues. Mast cell degranulation leads to vasoactive mediator release and clinically presents as episodic anaphylactoid symptoms (pruritis, flushing, headache, etc.). When the manifestations of these disorders are limited to the skin, this condition is referred to as cutaneous mastocytosis (CM). Patients with mastocytosis are at increased risk of anaphylaxis. Limited available data suggests that most cases of pediatric CM resolve or spontaneously improve by adolescence. However, the disease can be disfiguring, disabling and potentially life-threatening to children who develop systemic mast cell mediator release. There is insufficient data on clinical characteristics of patients with CM as well as disease triggers and management. Given the paucity of data, we have established a mastocytosis registry to understand the clinical characteristics, management and natural history of cutaneous mastocytosis.

**Methods:** In 2019 we established a registry to understand the clinical characteristics, management and natural history of CM. We are recruiting participants from dermatology and allergy clinics in Montreal and the Mastocytosis Society Canada and have developed a standardized questionnaire to assess these individuals.

**Results:** We have so far recruited eleven participants. Among our participants, 72% are children (less than 18 years old) and 82% have been diagnosed with CM. The major flares were related to specific physical stimuli (81%), ingestion of specific foods (55%) and antibiotic use (18%). Among the participants, 55% were treated using antihistamines and 18% with steroids; 36% reported having experienced remission without treatment.

**Conclusion:** We have established the first Canadian registry for mastocytosis. Our next steps will be to expand our registry to provide further data on diagnosis, flares and management of mastocytosis to improve the care for these patients and further develop this field of study.

### #70 Sustained effect of dupilumab with concomitant topical corticosteroids (TCS) on health-related quality of life (HRQoL) in patients with moderate-to-severe atopic dermatitis (AD): LIBERTY AD CHRONOS

#### Kim A. Papp^1^, Pedro Herranz Pinto^2^, Diana Rubel^3^, Luca Stingeni^5^, Annamaria Offidani^6^, Karel Ettler^7^, Ana B. Rossi^8^, Zhen Chen^9^, Paul Tomondy^8^, Brad Shumel^9^, Laurent Eckert^10^, Kelly Shaw^11^, Abhijit Gadkari^9^

##### ^1^K. Papp Clinical Research and Probity Medical Research, Waterloo, ON, Canada; ^2^Department of Dermatology, La Paz University Hospital, Madrid, Spain; ^3^Australian National University, Canberra, ACT, Australia; ^4^Probity Medical Research, Phillip, ACT, Australia; ^5^Department of Medicine (Section of Clinical, Allergological and Venereological Dermatology), University of Perugia, Perugia, Italy; ^6^Department of Clinical and Molecular Sciences, Polytechnic Marche University, Ancona, Italy; ^7^Faculty Hospital, Charles University, Hradec Králové, Czech Republic; ^8^Sanofi Genzyme, Cambridge, MA, USA; ^9^Regeneron Pharmaceuticals, Inc., Tarrytown, NY, USA; ^10^Sanofi, Chilly-Mazarin, France; ^11^Sanofi Genzyme, Toronto, Canada

###### **Correspondence:** Kelly Shaw

*Allergy Asthma Clin Immunol* 2020, **16(Suppl 1):**#70

**Background:** Dupilumab, a fully human anti-IL-4Rα mAb that inhibits IL-4/IL-13, is approved for patients aged ≥ 12 years in the USA with moderate-to-severe atopic dermatitis (AD) inadequately controlled by topical prescription treatments or when those therapies are not advisable, adult AD patients in Japan not adequately controlled with existing therapies, and moderate to severe AD patients aged ≥ 18 years in Europe who are candidates for systemic therapy. Here we evaluate the effect on quality-of-life in patients treated with dupilumab + topical corticosteroids (TCS) using data from a phase 3 trial (CHRONOS: NCT02260986).

**Methods:** Adults received dupilumab 300 mg weekly (qw + TCS)/every 2 weeks (q2w + TCS) or placebo + TCS for 52 weeks. Outcomes included total Dermatology Life Quality Index (DLQI) and 6 subdomains scores: symptoms and feelings, daily activities, leisure, personal relationships (maximum score 6), work and school, treatment burden (maximum score 3).

**Results:** In all DLQI domains, dupilumab treatment (vs placebo + TCS) showed improvements from baseline at both Week 16 and 52. Week 16 (qw + TCS/q2w + TCS vs. placebo + TCS): symptoms and feelings, − 2.90/− 2.64 vs. − 1.66; daily activities, − 2.38/− 2.12 vs. − 1.35; leisure, − 2.13/− 1.96 vs. − 1.23; personal relationships, − 1.31/− 1.29 vs. − 0.68; work and school, − 1.12/− 1.06 vs. − 0.65; treatment burden, − 0.94/− 0.94 vs. − 0.43. Week 52 (qw + TCS/q2w + TCS vs. placebo + TCS): symptoms and feelings, − 2.93/− 2.93 vs. − 1.87; daily activities, − 2.41/− 2.47 vs. − 1.58; leisure, − 2.19/− 2.23 vs. − 1.55; personal relationships, − 1.31/− 1.37 vs. − 0.88; work and school, − 1.08/− 1.13 vs. − 0.71; treatment burden, − 0.96/− 0.99 vs. − 0.45. Dupilumab improved total DLQI score at Week 16 (qw + TCS/q2w + TCS vs. placebo + TCS): − 10.70/− 10.00 vs. − 5.80; and Week 52 (qw + TCS/q2w + TCS vs. placebo + TCS): − 10.80/− 11.00 vs. − 6.80 (all *P* values< 0.0001). Dupilumab had an acceptable safety profile.

**Conclusions:** Adults with moderate-to-severe AD treated with dupilumab + TCS had improvements in total DLQI and all its domains vs placebo + TCS at Week 16, sustained through Week 52.

**Acknowledgments:** Research sponsored by Sanofi and Regeneron Pharmaceuticals, Inc. ClinicalTrials.gov identifier: NCT02260986 (LIBERTY AD CHRONOS). Medical writing/editorial assistance provided by Natalie Adlesic, PhD, of Excerpta Medica, funded by Sanofi Genzyme and Regeneron Pharmaceuticals, Inc.

### #71 Expression profiles of primary immunodeficiency associated genes in adult cancers and their incidence and implications in pediatric malignancies

#### Son Tran^1^, Sunand Kannappan^1^, Tony H. Truong^1^, Luis Murguia-Favela^2^, Pinaki Bose^3^, Aru Narendran^1^

##### ^1^Division of Pediatrics, Section of Pediatric Oncology and Blood and Marrow Transplantation, Alberta Children’s Hospital and University of Calgary, Calgary, AB, Canada; ^2^Division of Pediatrics, Section of Pediatric Hematology and Immunology, Alberta Children’s Hospital and University of Calgary, Calgary, AB, Canada; ^3^Departments of Oncology, Biochemistry and Molecular Biology, University of Calgary, Calgary, AB, Canada

###### **Correspondence:** Son Tran

*Allergy Asthma Clin Immunol* 2020, **16(Suppl 1):**#71

**Background:** The immune surveillance concept postulates that primary immune deficiencies (PID) are an important contributor to cancer growth and outcomes. However, the molecular mechanisms that underlie immune evasion of pediatric cancers are poorly defined.

**Methods:** Microarray data from normal tumor-adjacent samples was obtained from Gene Expression Omnibus (GEO) database for colorectal cancer (n = 155) and pancreatic cancer (n = 42). PID-related genes were curated from the Fulgent Genetics “Comprehensive Primary Immunodeficiency NGS Panel”. univariate and multivariate Cox regression analyses for PID-related genes and clinical covariates were performed with overall survival as an outcome. Data from the TARGET datasets in cBioPortal were used to generate Kaplan–Meier survival curves to assess the clinical impact of alterations of PID-related genes in pediatric cancers.

**Results:** PID-related genes significantly associated with poor prognosis were *OSTM1* (HR: 3.667 [1.131–11.888]) and *F11* (HR: 1.704 [1.015–2.859]) for colorectal cancer. PID-related genes significantly associated with improved prognosis were *HYDIN* (HR: 0.525 [0.350–0.788]), *NHP2* (HR: 0.833 [0.718–0.966]), and *FANCA* (HR: 0.298 [0.126–0.707]) for colorectal cancer and *RPS27A* (HR: 0.044 [0.005–0.375]), *TCF3* (HR: 0.044 [0.002–0.898]), and *C8G* (HR: 0.132 [0.024–0.732]) for pancreatic cancer. The frequency of amplification, deletion and mutation of the eight genes across two pediatric hematological malignancies (ALL, AML) and three solid tumors (Wilms, rhabdoid, and neuroblastoma) was 3.31%, with the greatest frequency in ALL at 5.06%. In the Wilms and neuroblastoma datasets, alterations in the identified genes significantly lowered survival (P < 0.001).

**Conclusion:** Our studies identified specific PID-related genes with clinical significance in distinct pediatric tumors. These data may help identify important biomarkers and potential future targeted therapeutics.

### #72 Hypogammaglobulinemia may affect the course of COPD in hospitalized patients

#### Harissios Vliagoftis^1,3^, Malcena Niven^1,3^, Nami Shrestha^1,3^, Desi Fuhr^1,3^, Brian Rowe^2,3^, Mohit Bhutani^1,3^, Michael Stickland^1,3^

##### ^1^Division of Pulmonary Medicine, Department of Medicine, University of Alberta; ^2^Department of Emergency Medicine, University of Alberta; ^3^Alberta Respiratory Centre, University of Alberta

###### **Correspondence:** Harissios Vliagoftis

*Allergy Asthma Clin Immunol* 2020, **16(Suppl 1):**#72

**Background:** Chronic obstructive pulmonary disease (COPD) is a progressive obstructive lung disease that affects up to 4% of Canadians. COPD patients may be immunocompromised because of their chronic disease, poor nutrition or frequent courses of oral corticosteroids. These patients may present with secondary hypogammaglobulinemia that might predispose them to frequent and more severe infections. We hypothesized that decreasing serum IgG levels are associated with acute COPD exacerbations (AECOPD) leading to increased frequency of emergency department (ED) visits and hospitalizations.

**Methods:** A prospective study to examine inflammation and cardiovascular risk in patients hospitalized for COPD recruited patients between December 2014 and July 2017. Data on length of hospitalization at the index admission, subsequent number of ED visits, and hospital readmission rates were collected. Immunoglobulin levels (IgG, IgA and IgM) were measured in serum samples collected prospectively at recruitment.

**Results:** 55 patients had a full set of data; 37 subjects had normal serum IgG levels (7.0–16.18 g/L) and 15 had hypogammaglobulinemia (IgG < 7.0 g/L). 3 subjects with IgG levels over the normal range were excluded. There was no difference in outcomes between subjects with normal and low IgG levels. In the low IgG group, there was a negative correlation between the number of AECOPD-related hospital readmissions and serum IgG (R = − 0.542, p = 0.037), while no such correlation existed in the patients with normal IgG. Subjects with low IgM levels had longer hospital length of stay at index admission compared to subjects with normal IgM (7.4 ± 5.7 vs. 4.3 ± 3.5 days, p = 0.011).

**Conclusion:** In patients with COPD and hypogammaglobulinemia IgG levels may be predictive of the course of the disease. In addition, low IgM may also affect clinical outcomes in COPD patients. The biological significance of this observation is not clear, and larger studies are required to understand these associations.

### #73 Delivery of subcutaneous immunoglobulin by rapid “push” infusion for primary immunodeficiency patients—revised

#### Graham Walter^1^, Chrystyna Kalicinsky^1,2^, Marianne Miguel^1,2^, Tamar S. Rubin^3,4^

##### ^1^Department of Internal Medicine, University of Manitoba, Winnipeg, Canada; ^2^Section of Allergy and Clinical Immunology, University of Manitoba, Winnipeg, Canada; ^3^Department of Pediatrics and Child Health, University of Manitoba, Winnipeg, Canada; ^4^Section of Pediatric Allergy and Clinical Immunology, University of Manitoba, Winnipeg, Canada

###### **Correspondence:** Tamar S. Rubin

*Allergy Asthma Clin Immunol* 2020, **16(Suppl 1):**#73

**Background:** Pump-free subcutaneous immunoglobulin (SCIG) self-administration by rapid push has been reported as a safe, effective and convenient method of immune globulin (Ig) replacement in patients with primary immune deficiency diseases (PID).

**Methods:** We conducted a retrospective review of all patients enrolled in the SCIG push program in Manitoba, Canada. We also included adult patients (≥ 18 years old) receiving SCIG push for PID for at least 12 months. Eligible patients were either Ig replacement naïve, or had previously received Ig by intravenous infusion (IVIG). We extracted individual patient IgG levels before and after starting SCIG, as well as the number of systemic antibiotic courses filled before and after starting SCIG. We also reviewed patient-reported adverse events, and reasons for discontinuation of the program.

**Results:** Sixty-five patients were included, of whom twenty-eight were naïve to Ig replacement, and thirty-seven were on IVIG replacement prior. Of naïve patients, the mean IgG levels 6 months prior to SCIG and 12 months after were 5.22 g/L (range: < 0.33–12.3 g/L), and 10.75 g/L (range: 5.85–16.1 g/L) respectively. The mean numbers of antibiotic prescriptions in the 12 months prior and 12 months after were 5.50 (range: 1–14) and 4.04 (range: 0–16) respectively. With regard to hospitalizations, IVIG naïve patients were hospitalized an average of 0.35 (range 0–4) times in the 12 months preceding SCIG replacement and 0.21 (range 0–3) in the 12 months following. For patients in the IVIG-prior group, the mean IgG levels 6 months prior and 12 months after were 11.67 g/L (range: 6.76–45.9 g/L), and 13.07 g/L (range: 4.99–44.0 g/L). The average number of antibiotic prescriptions 12 months prior and 12 months for this group after starting SCIG were 4.23 (range: 0–20) and 3.63 (range: 0–16). The average number of hospitalizations in the preceding 12 months was 0.30 (range: 0–4) and 0.22 in the 12 months following SCIG switch (range 0–3). Adverse events were generally local and mild, with only two patients discontinuing SCIG replacement secondary to side effects.

**Conclusions:** SCIG push appears to provide adequate steady-state IgG concentrations in our local adult PIDD population, and resulted in a reduction in both antibiotic prescriptions filled and hospitalizations in the IgG replacement naïve population. Compared to IVIG replacement, push SCIG also appeared to provide a similar steady-state IgG concentration as well as number of hospitalizations and antibiotic prescriptions filled.

### #74 Signs, symptoms, and quality of life in atopic dermatitis: early and sustained clinically meaningful responses with dupilumab and concomitant topical corticosteroids: a phase 3 trial post hoc analysis

#### Marjolein de Bruin-Weller^1^, Christopher E. M. Griffiths^2,3^, Errol Prens^4^, Jacek C. Szepietowski^5^, Takafumi Etoh^6^, Ana B. Rossi^7^, Abhijit Gadkari^8^, Laurent Eckert^9^, Zhen Chen^8^, Kelly Shaw^10^, Brad Shumel^8^

##### ^1^University Medical Center Utrecht, Utrecht, Netherlands; ^2^The Dermatology Centre, Salford Royal Hospital, Manchester, UK; ^3^University of Manchester, Manchester Academic Health Science Centre, Manchester, UK; ^4^Erasmus University Medical Center Rotterdam, Rotterdam, Netherlands; ^5^Department of Dermatology, Venereology and Allergology, Wrocław Medical University, Wrocław, Poland; ^6^Tokyo Teishin Postal Services Agency Hospital, Tokyo, Japan; ^7^Sanofi Genzyme, Cambridge, MA, USA; ^8^Regeneron Pharmaceuticals Inc., Tarrytown, NY, USA; ^9^Sanofi, Chilly-Mazarin, France; ^10^Sanofi Genzyme, Toronto, Canada

###### **Correspondence:** Kelly Shaw

*Allergy Asthma Clin Immunol* 2020, **16(Suppl 1):**#74

**Background:** Dupilumab is approved for patients aged ≥ 12 years in the USA with moderate-to-severe atopic dermatitis (AD) inadequately controlled by topical prescription treatments or when those therapies are not advisable, adult AD patients in Japan not adequately controlled with existing therapies, and moderate to severe AD patients aged 18 years and older in Europe who are candidates for systemic therapy. This post hoc analysis describes the proportion of patients with moderate-to-severe AD treated with dupilumab plus concomitant topical corticosteroids (TCS) who achieved clinically meaningful responses in ≥ 1 of the 3 major AD domains (signs, symptoms, and quality of life [QoL]) using data from a long-term phase 3 trial (CHRONOS: NCT02260986).

**Methods:** Patients were randomized 3:1:3 to subcutaneous dupilumab 300 mg plus concomitant TCS weekly (qw + TCS), every 2 weeks (q2w + TCS), or placebo + TCS for 52 weeks. Clinically meaningful responses were defined as: ≥ 50% reduction from baseline in the Eczema Area and Severity Index score, or ≥ 3-point reduction from baseline in weekly average Peak daily Pruritus Numerical Rating Scale score, or ≥ 4-point reduction from baseline in the Dermatology Life Quality Index score.

**Results:** 315/106/319 patients were randomized to placebo + TCS/q2w + TCS/qw + TCS. More patients achieved ≥ 1 clinically meaningful outcomes with dupilumab + TCS vs placebo + TCS as early as Week1 (qw + TCS/q2w + TCS vs. placebo + TCS: 62.7%/61.3% vs. 52.1%; *P *= 0.0067/*P *= 0.0972). At Week2, 80.6%/81.1% of patients in the qw + TCS/q2w + TCS groups reached ≥ 1 meaningful outcome vs. 60.3% of patients receiving placebo + TCS. These results were sustained or further improved at Week52 (qw + TCS/q2w + TCS vs. placebo + TCS: 72.1%/79.2% vs. 36.2%) (*P *< 0.0001 for all timepoints Week2–Week52). Injection-site reactions and conjunctivitis were more frequent with dupilumab than placebo.

**Conclusions:** In this long-term phase 3 trial, a higher proportion of patients with moderate-to-severe AD treated with dupilumab + TCS had clinically meaningful improvement in 1 or more key AD domains (signs, symptoms, and QoL) as early as Week1 and through Week52.

**Acknowledgments:** Research sponsored by Sanofi and Regeneron Pharmaceuticals, Inc. ClinicalTrials.gov Identifier: NCT02260986 (LIBERTY AD CHRONOS). Medical writing/editorial support provided by Patricia Gómez-Pérez, MD, MPH, of Excerpta Medica, funded by Sanofi Genzyme and Regeneron Pharmaceuticals, Inc.

### #75 Effect of Dupilumab on health-related quality of life (HRQoL) in patients with moderate-to-severe atopic dermatitis (AD): results from two identical randomized phase 3 trials (LIBERTY AD SOLO 1 and 2)

#### Jean-Philippe Lacour^1^, Andreas Wollenberg^2^, Thomas Werfel^3^, Yoko Kataoka^4^, H. Chih-ho Hong^5^, Paolo Amerio^6^, Maria Concetta Fargnoli^7^, Kϋlli Kingo^8^, Seong-Jun Seo^9^, Giovanna Moretti^10^, Ana B. Rossi^11^, Zhen Chen^12^, Paul Tomondy^11^, Marius Ardeleanu^12^, Abhijit Gadkari^12^, Kelly Shaw^13^, Laurent Eckert^14^

##### ^1^Department of Dermatology, Centre Hospitalier Universitaire de Nice, Nice, France; ^2^Ludwig-Maximilian-University, Munich, Germany; ^3^Hannover Medical University, Hannover, Germany; ^4^Department of Dermatology, Osaka Habikino Medical Center, Habikino, Japan; ^5^Probity Medical Research, Surrey, BC, Canada; ^6^Dermatologic Clinic, University “G. d’Annunzio” Chieti – Pescara, Chieti, Italy; ^7^Department of Dermatology, University of L’Aquila, L’Aquila, Italy; ^8^Clinic of Dermatology, Tartu University Hospital, Tartu, Estonia; ^9^Chung-Ang University Hospital, Seoul, Republic of Korea; ^10^Department of Dermatology, Papardo Hospital, Messina, Italy; ^11^Sanofi Genzyme, Cambridge, MA, USA; ^12^Regeneron Pharmaceuticals, Inc., Tarrytown, NY, USA; ^13^Sanofi Genzyme, Toronto, Canada; ^14^Sanofi, Chilly-Mazarin, France

###### **Correspondence:** Kelly Shaw

*Allergy Asthma Clin Immunol* 2020, **16(Suppl 1):**#75

**Background:** Dupilumab, a fully human anti-IL-4Rα mAb, inhibits signaling of IL-4/IL-13, key drivers of type 2-mediated inflammation. Dupilumab is approved for patients aged ≥ 12 years in the USA with moderate-to-severe atopic dermatitis (AD) inadequately controlled by topical prescription treatments or when those therapies are not advisable, adult AD patients in Japan not adequately controlled with existing therapies, and moderate to severe AD patients aged ≥ 18 years in Europe who are candidates for systemic therapy. AD has an important impact on patients’ quality-of-life, commonly assessed with the Dermatology Life Quality Index (DLQI) questionnaire. Here we report the effect of dupilumab treatment on quality-of-life by using pooled data from two 16-week phase 3 trials (LIBERTY AD SOLO 1: NCT02277743; LIBERTY AD SOLO 2: NCT02277769).

**Methods:** Adults with moderate-to-severe AD were randomized to dupilumab 300 mg weekly (qw), every 2 weeks (q2w), or placebo qw for 16 weeks. Outcomes included total DLQI score and its 6 domains: symptoms and feelings, daily activities, leisure, personal relationships (maximum score 6), work and school, and treatment burden (maximum score 3).

**Results:** 1379 patients were randomized to qw (n = 462), q2w (n = 457), or placebo (n = 460). Dupilumab resulted in improvements from baseline in all DLQI domains at Week 16 (qw/q2w vs. placebo: symptoms and feelings, − 2.45/− 2.41 vs. − 1.23; daily activities, − 2.16/− 2.19 vs. –1.04; leisure, − 1.86/− 1.88 vs. − 1.02; personal relationships, − 1.17/− 1.18 vs. − 0.50; work and school, − 0.97/− 0.99 vs. − 0.43; treatment burden, − 0.73/− 0.72 vs. − 0.37). Dupilumab treatment also resulted in improvement in total DLQI score at Week 16 (qw/q2w vs. placebo): –9.20/–9.30 vs. − 4.30 (all *P *< 0.0001). Dupilumab had an acceptable safety profile.

**Conclusions:** Adults with moderate-to-severe AD treated with dupilumab monotherapy consistently had improvements in total DLQI score and scores in all its 6 domains compared with placebo at Week 16.

**Acknowledgments:** Research sponsored by Sanofi and Regeneron Pharmaceuticals, Inc. ClinicalTrials.gov Identifiers: NCT02277743 (LIBERTY AD SOLO 1), NCT02277769 (LIBERTY AD SOLO 2). Medical writing/editorial assistance provided by Natalie Adlesic, PhD, of Excerpta Medica, funded by Sanofi Genzyme and Regeneron Pharmaceuticals, Inc.

## Other allergy/immunology

### #76 Defining beta-lactam allergy documentation in Canadian primary care practices

#### Elissa M. Abrams^1^, Ryan R. Phung^2^, Alexander G. Singer^3^

##### ^1^Section of Allergy and Immunology, Department of Pediatrics, University of Manitoba, Winnipeg, MB, Canada; ^2^Max Rady College of Medicine, University of Manitoba, Winnipeg, MB, Canada; ^3^Department of Family Medicine, University of Manitoba, Winnipeg, MB, Canada

###### **Correspondence:** Elissa M Abrams

*Allergy Asthma Clin Immunol* 2020, **16(Suppl 1):**#76

**Background:** Research conducted in the U.S has determined the prevalence of reported beta-lactam allergy to be between 10 and 17%. Currently, there is no data to date on the prevalence of self-reported or documented beta-lactam allergy in Canada. The purpose of this study is to describe the prevalence of physician-documented beta-lactam allergy in a Canadian outpatient population and to comment on associated characteristics of patients and providers.

**Methods:** We conducted a retrospective cohort study using Electronic Medical Record (EMR) data from the Manitoba Primary Care Research Network (MaPCReN). An algorithm was generated to define and extract data on physician documented beta-lactam allergy from the EMR dataset (n = 211,132). Once established; prevalence, provider, and patient variables were analyzed using multivariate regression to assess differences between patients with reported beta-lactam allergy and those without.

**Results:** Of the 221,132 records in the MaPCReN database, 2.89% (6397/221,132) of patients had a recorded beta-lactam allergy. Documented beta-lactam allergy was found to be associated with female sex (1.542 OR, 95% CI 1.463-1.623) and medical comorbidities including asthma and eczema (P < .0001 for both asthma and eczema).

**Conclusions:** Prevalence of physician reported beta-lactam allergy in Manitoba was lower than previously recorded American studies. The validated algorithm will be applied to the larger Canadian Primary Care Sentinel Surveillance Network repository to describe epidemiology at a national scale and to further aid efforts in reducing burden associated with erroneous labelling.

### #77 Diagnosing serum sickness-like reactions using a graded oral challenge

#### Luca Delli Colli^1^, Sofianne Gabrielli^1^, Elissa M. Abrams^2,3^, Elana Lavine^4,5,6^, Tracy Pitt^5,6,7^, Adelle Atkinson^4^, Thomas Eiwegger^4^, Christine McCusker^1^, Moshe Ben-Shoshan^1^

##### ^1^Division of Allergy and Clinical Immunology, Department of Pediatrics, Montreal Children’s Hospital, Montreal, QC, Canada; ^2^Department of Pediatrics, Section of Allergy and Clinical Immunology, University of Manitoba, Winnipeg, MB, Canada; ^3^Department of Pediatrics, Division of Allergy and Immunology, University of British Columbia, Vancouver, BC, Canada; ^4^Division of Allergy and Clinical Immunology, Department of Paediatrics, University of Toronto, Hospital for Sick Children, Toronto, Canada; ^5^Department of Pediatrics, Queen’s University, Kingston, ON, Canada; ^6^Department of Pediatrics, Humber River Hospital, Toronto, ON, Canada; ^7^Department of Pediatrics, St Joseph’s Hospital, Toronto, ON, Canada

###### **Correspondence:** Luca Delli Colli

*Allergy Asthma Clin Immunol* 2020, **16(Suppl 1):**#77

**Background:** Serum sickness-like reactions (SSLR) are benign reactions defined by presence of rash (primarily urticaria), and joint complaints (arthralgia/arthritis) that are believed to occur due to a non-IgE mediated response to drugs and/or viral infections [1]. Thus, determining the etiology of any given episode is challenging. We aim to evaluate children presenting with SSLRs associated with antibiotic use through a graded oral challenge.

**Methods:** All children referred to the Montreal Children’s Hospital for potential antibiotic allergy and a clinical presentation compatible with SSLR were recruited for the study between March 2013 and June 2019. A standardized survey with questions on treatment, symptoms, and associated factors was completed and an oral challenge (10% and subsequently 90% of the oral dose) was conducted. Patients with a negative challenge were contacted annually to query on subsequent antibiotic use.

**Results:** Among 50 patients presenting with suspected SSLRs, the median age was 2.65 years (IQR 0.98, 4.33) and 52.0% were males. The majority, 84.0%, of patients reported reactions to Amoxicillin, 12.0% reacted to Clavulin and 4.00% reacted to Cefprozil. The most common symptoms were arthritis/arthralgia (100.0%), urticaria (68.0%), and macular/papular rash often associated with hemorrhagic lesions (44.0%). Amongst the 50 patients, 4.00% reacted immediately to a graded oral challenge (within 1 h) and 4.00% had a non-immediate reaction. Of the 31 patients who responded to our follow-up, 23 (74.2%) reported subsequent antibiotic use of which 13 patients (56.5%) underwent subsequent treatment with the culprit antibiotic. Among those who reported subsequent culprit antibiotic use, 5 (38.5%) reported a mild rash.

**Conclusion:** This is the first and largest pediatric study assess SSLR through the use of oral challenge. Our data suggests that challenge is a safe and accurate procedure in this population. Given our findings, it is likely that viral infections rather than true antibiotic allergy account for most SSLRs in children.

**Ethics statement:** McGill University Health Center Research Ethics Board. REB # 12-084 PED.

#### Reference

1. Yorulmaz A, Akın F, Sert A, Agir MA, Yilmaz R, Arslan S. Demographic and clinical characteristics of patients with serum sickness-like reaction. Clin Rheumatol. 2018;37:1389–94.

### #78 Dupilumab reduces systemic corticosteroid use and sinonasal surgery in patients with severe chronic rhinosinusitis with nasal polyps: pooled results from the phase 3 SINUS-24 and SINUS-52 studies

#### Martin Desrosiers^1^, Claus Bachert^2,3^, Peter Hellings^4^, Claire Hopkins^5^, Heidi Olze^6^, Joseph K. Han^7^, Stella E. Lee^8^, Mei Zhang^9^, Xin Lu^9^, Nikhil Amin^10^, Naimish Patel^9^, Neil M.H. Graham^10^, Marcella Ruddy^10^, Heribert Staudinger^9^, Leda P. Mannent^11^

##### ^1^Département Oto-rhino-laryngologie (ORL), Centre de recherche du Centre hospitalier de l’Université de Montréal (CRCHUM), Montreal, QC, Canada; ^2^Upper Airways Research Laboratory, Ghent University, Ghent, Belgium; ^3^Department of Clinical Science, Intervention and Technology (CLINTEC), Karolinska Institutet, Stockholm, Sweden; ^4^Department of Otorhinolaryngology, University Hospitals Leuven, Leuven, Belgium; ^5^ENT Department, Guy’s and St Thomas’ Hospitals, London, UK; ^6^Department of Otorhinolaryngology, Head and Neck Surgery, Charité-Universitätsmedizin Berlin, Berlin, Germany; ^7^Department of Otolaryngology, Eastern Virginia Medical School, Norfolk, VA, USA; ^8^Department of Otolaryngology, University of Pittsburgh Medical Center, Pittsburgh, PA, USA; ^9^Sanofi, Bridgewater, NJ, USA; ^10^Regeneron Pharmaceuticals, Inc., Tarrytown, NY, USA; ^11^Sanofi, Chilly-Mazarin, France

###### **Correspondence:** Martin Desrosiers

*Allergy Asthma Clin Immunol* 2020, **16(Suppl 1):**#78

**Background:** Current treatment paradigm for chronic rhinosinusitis with nasal polyps (CRSwNP) is characterized by recurrent surgeries and/or frequent systemic corticosteroid (SCS) use. Dupilumab, a fully human monoclonal antibody that blocks the shared receptor component for interleukin (IL)-4 and IL-13, key drivers of type 2-mediated inflammation. This prespecified analysis evaluates the effect of dupilumab on SCS use and nasal polyp (NP) surgery in patients with CRSwNP previously treated with SCS and/or surgery, receiving mometasone furoate and standard of care in a pooled SINUS-24 (NCT02912468)/SINUS-52 (NCT02898454) population.

**Methods:** SINUS-24 patients were randomized 1:1 to subcutaneous (SC) dupilumab 300 mg or placebo every 2 weeks (q2w) for 24 weeks. SINUS-52 patients were randomized 1:1:1 to 52 weeks of SC dupilumab 300 mg q2w, 24 weeks q2w followed by 28 weeks of dupilumab 300 mg every 4 weeks, or 52 weeks of placebo q2w. Pooled analysis included all patients randomized to dupilumab 300 mg q2w (n = 438) and placebo (n = 286) over the 24- and 52-week treatment periods.

**Results:** Baseline disease characteristics were comparable between groups. 74.3% of patients used SCS in the past 2 years; 63.4% had prior NP surgery. Dupilumab vs. placebo reduced the proportion of patients requiring SCS rescue by 73.9%, number of SCS courses by 75.3%, and need for NP surgery by 82.6% (nominal *P* vs. placebo: < 0.001). Dupilumab vs. placebo reduced NP score and nasal congestion in patients with prior SCS use (difference vs. placebo − 1.83 and − 0.88 [nominal *P* vs. placebo: < 0.0001]), and in patients with prior NP surgery (− 1.99 and − 0.98 [nominal *P* vs. placebo: < 0.0001]). Common adverse events (≥ 5%) were nasopharyngitis, NP, headache, asthma, epistaxis, and injection-site erythema, all occurring with higher frequency in placebo-treated patients.

**Conclusions:** Dupilumab significantly reduced SCS use and NP surgery in patients with severe CRSwNP in the pooled SINUS-24/SINUS-52 population and was well tolerated.

**Acknowledgments and funding sources:** Research sponsored by Sanofi and Regeneron Pharmaceuticals, Inc. ClinicalTrials.gov Identifiers: NCT02912468 and NCT02898454. Medical writing/editorial assistance provided by Maggie Tarrio Watson, PhD, of Excerpta Medica, funded by Sanofi Genzyme and Regeneron Pharmaceuticals, Inc.

### #79 Assessing risk factors associated with a positive graded oral challenge (GOC) in children with suspected amoxicillin allergy

#### Rutherford Exius^4^, Sofianne Gabrielli^2,4^, Christine McCusker^1,2,3,4^, Andrew O’Keefe^5^, Elissa M. Abrams^6^, Jennifer Protudjer^6,11,12,13,14^, Elana Lavine^7^, Tracy Pitt^8^, Adelle Atkinson^9^, Thomas Eiwegger^9,10^, Moshe Ben-Shoshan^1,2,3,4^

##### ^1^Division of Clinical Epidemiology, Department of Medicine, McGill University Health Center, Montreal, QC, Canada; ^2^Division of Pediatric Allergy and Clinical Immunology, Department of Pediatrics, Montreal Children’s Hospital, McGill University Health Center, Montreal, QC, Canada; ^3^Division of Dermatology, Department of Medicine, McGill University Health Center, Montreal, QC, Canada; ^4^Division of Experimental Medicine, The Research Institute of the McGill University Health Centre, McGill University, Montreal, QC, Canada; ^5^Nl Allergy and Immunology, St John’s, Newfoundland, Canada; ^6^Department of Pediatrics and Child Health, Section of Allergy and Clinical Immunology, University of Manitoba, Winnipeg, MB, Canada; ^7^Humber River Hospital, Toronto, Ontario, Canada; ^8^Midtown pediatrics, Toronto, ON, Canada; ^9^Division of Allergy and Immunology, Hospital for Sick Children, Toronto, ON, Canada; ^10^Division of physiology and experimental Medicine, University of Toronto, Toronto, ON, Canada; ^11^Children’s Hospital Research Institute of Manitoba, Winnipeg, ON, Canada; ^12^George and Fay Yee Centre for Healthcare Innovation, Winnipeg, MB, Canada; ^13^Department of Food and Human Nutritional Sciences, University of Manitoba, Winnipeg, MB, Canada; ^14^Institute of Environmental Medicine, Karolinska Institutet, Stockholm, Sweden

###### **Correspondence:** Rutherford Exius

*Allergy Asthma Clin Immunol* 2020, **16(Suppl 1):**#79

**Background:** The diagnosis of amoxicillin hypersensitivity is challenging in children given that the sensitivity of commercially available skin tests is low and oral challenges are often required to establish the diagnosis. We aimed to assess the diagnostic properties of a graded oral challenge (GOC) among children presenting with a rash during amoxicillin therapy and determine risk factors associated with positives challenges

**Methods:** A cohort study was conducted between May 1, 2013, and May 17, 2019, at the allergy clinic of the Montreal Children’s Hospital. All children referred with suspected allergy to amoxicillin were recruited and a GOC was conducted. Eligible children were followed up to assess reactions to subsequent use of amoxicillin. Data were collected on demographics clinical characteristics of reaction and comorbidities.

**Results:** We recruited 1632 children (median age at reaction, 1.7 years [interquartile range, 1.0–3.5 years]; 876 [53.7%] male). Among them, 1544 (94.6%) tolerated the GOC, 38 (2.3%) developed mild immediate reactions, and 50 (3.1%) developed nonimmediate reactions. The GOC had a negative predictive value of 84.8%. Among all 1461 participants eligible for annual follow-up, 817 (55.9%) responded, 257 of whom received subsequent full treatment with amoxicillin; 218 of these 257 participants (84.8%) reported tolerance, while 39 (15.2%) had mild reactions. History of a reaction occurring within 5 min of exposure (adjusted Odds Ratio [aOR] = 1.11; 95%CI 1.07–1.17) and chronic urticaria (aOR = 1.11; 95%CI 1.05–1.19) were associated with immediate reactions to the GOC. A rash lasting longer than 7 days (aOR = 1.09; 95%CI 1.02–1.17) and self-reported maternal drug allergy (aOR = 1.04; 95%CI 1.00–1.07) were associated with nonimmediate reactions.

**Conclusions:** GOCs provide an accurate and safe confirmatory test for skin-related reactions to amoxicillin. Further studies are required to assess factors associated with the GOC outcome groups and the cross-reactivity between penicillin derivatives and cephalosporins.

McGill University Health Center Research Ethics Board. REB # 12-084 PED.

### #80 Dairy quality amongst adolescents with a history of eczema: results from the SAGE Birth Cohort

#### Hailey Hildebrand^1^, Anita L Kozyrskyj^2^, Allan B Becker^3,4^, Jennifer L. P. Protudjer^3,4,5,6,7^

##### ^1^Department of Medicine, University of Manitoba, Winnipeg, MB, Canada; ^2^Department of Pediatrics, University of Alberta, Edmonton, AB, Canada; ^3^Department of Pediatrics and Child Health, University of Manitoba, Winnipeg, MB, Canada; ^4^Children’s Hospital Research Institute of Manitoba, Winnipeg, MB, Canada; ^5^George and Fay Yee Centre for Healthcare Innovation, Winnipeg, MB, Canada; ^6^Department of Food and Human Nutritional Sciences, University of Manitoba, Winnipeg, MB, Canada; ^7^Institute of Environmental Medicine, Karolinska Institutet, Stockholm, Sweden

###### **Correspondence:** Hailey Hildebrand

*Allergy Asthma Clin Immunol* 2020, **16(Suppl 1):**#80

**Background:** Eczema may affect diet quality. The aim of this study was to examine the dairy quality amongst Manitoba adolescents with a history of eczema.

**Methods:** We examined dairy quality using data from a cohort of Manitoba children born in 1995 and followed to adolescence. At ages 7–8 (childhood) and 12–14 (adolescence) years, parents reported if their child had eczema. Persistent eczema was defined as eczema at both time points, whereas transient was eczema in childhood only. Adolescents completed food frequency questionnaires, which queried dairy intake, including skim/low-fat milk, homogenized milk, yogurt, and ice cream, on a scale from rarely to 6+ times daily. Dairy quality was classified based on the Youth Health Eating Index, with low-fat milk and yogurt consumption scored twice as high as high-fat, high-sugar dairy. Linear regression was used, with adjustments for confounders. This study was approved by The University of Manitoba Health Research Ethics Board (HS14742 (H2002:078)).

**Results:** Overall, 468 adolescents (263 (56.2%) boys) were included, including 22 (5%) with persistent and 63 (13%) with transient eczema. Mean dairy quality was poor (4.6/10) with boys tending to score slightly higher than girls (p = 0.10). Compared to those without eczema in childhood, those with eczema in childhood had poorer dairy quality in adolescence (ß − 0.53; 95%CI − 1.01; − 0.04). When stratified by sex, girls with eczema in childhood had poorer dairy quality in adolescence then girls without eczema in childhood (ß − 0.81; 95%CI − 1.51; − 0.10). In contrast, dairy quality did not differ in boys with or without eczema in childhood, or amongst adolescents with vs. without eczema. Compared to adolescents with persistent eczema, those with transient eczema tended to have lower dairy quality trending toward significance in partially adjusted models only (p < 0.07).

**Conclusion:** Unlike eczema in adolescence, eczema in childhood is associated with lower dairy quality in adolescence.

### #81 An algorithm-based approach to routinely delabel penicillin allergy in pre-hematopoietic stem cell transplant patients with low risk of reaction

#### Shun Chi Ryan Lo^1^, Katie Lacaria^2,3^, Allison Mah^4^, Tiffany Wong^5^, Raymond Mak^5^

##### ^1^Department of Medicine, University of British Columbia, Vancouver, BC, Canada; ^2^Faculty of Pharmaceutical Sciences, University of British Columbia, Vancouver, BC, Canada; ^3^Pharmaceutical Sciences, Vancouver General Hospital, Vancouver, BC, Canada; ^4^Division of Infectious Diseases, Department of Medicine, University of British Columbia, Vancouver, BC, Canada; ^5^Division of Allergy and Immunology, Department of Pediatrics, University of British Columbia, Vancouver, BC, Canada

###### **Correspondence:** Shun Chi Ryan Lo

*Allergy Asthma Clin Immunol* 2020, **16(Suppl 1):**#81

**Background:** Reported penicillin allergy is common (10% of population), however, 90% of these patients can tolerate the drug. Detailed penicillin history, skin testing, and oral challenge have been used to delabel this allergy. To date, few programs have focused on immunocompromised patients. We assessed a strategy of routine screening and assessment in pre-bone marrow transplant recipients.

**Methods:** Beginning in October 2018, a structured questionnaire was administered to pre-hematopoietic stem cell transplant patients reporting a history of penicillin allergy. Application of the history to an algorithm then identified low-risk patients for skin testing by a trained pharmacist. If skin-prick and intradermal testing to major and minor determinants were negative, oral challenge to amoxicillin was administered. Delayed reactions were evaluated by pharmacist phone call 72 h after the challenge. An allergist evaluated patients with high-risk or recent reactions.

**Results:** Fifteen patients with penicillin allergy were assessed. Three patients were referred to an allergist for serious (1) or recent (2) reactions. Two patients tolerated a penicillin-derivative drug after the reaction, while one patient was exposed to cephalosporins rather than penicillin, and thus were deemed not allergic on history. Nine patients were tested and had negative skin testing. No patients developed immediate reactions to oral amoxicillin challenge. One patient developed a delayed morbilliform rash. Ultimately, 13/15 (87%) of patients were delabelled. Average time for testing was 1.56 h. Total cost of reagents was $1234.08. Five delabelled patients subsequently tolerated courses of penicillin-derivatives.

**Conclusions:** Implementation of systematic assessment using a questionnaire, skin testing and oral challenge led by a clinical pharmacist successfully identified immunocompromised patients eligible for penicillin allergy delabelling. Most patients (87%) were delabelled and rate of reaction (6.7%) was similar to other reported strategies. No severe reactions, including anaphylaxis, occurred. Future directions include direct oral challenge of low-risk patients to increase cost-effectiveness.

### #82 Building a polygenic risk score to assess atopic dermatitis and allergic march in two Canadian cohorts

#### Mathieu Simard^1,2^, Anne-Marie Madore^1^, Anita Kozyrjsky^3^, Simon Girard^1,2^, Stuart E. Turvey^4^, Malcolm Sears^5^, Qingling Duan^6^, Padjama Subbarao^7^, Susan Waserman^8^, Philippe Bégin^9,10^, Catherine Laprise^1,2^

##### ^1^Département des sciences fondamentales, Université du Québec à Chicoutimi, Saguenay, QC, Canada; ^2^Centre Intersectoriel en Santé Durable, Université du Québec à Chicoutimi, Saguenay, QC, Canada; ^3^University of Alberta, Edmonton, AB, Canada; ^4^Department of Pediatrics Child and Family Research Institute, BC Children’s Hospital, University of British Columbia, Vancouver, BC, Canada; ^5^Department of Medicine, McMaster University, Hamilton, ON, Canada; ^6^School of Computing and Dept of Biomedical & Molecular Sciences, Queen’s University, Kingston, ON, Canada; ^7^Department of Pediatrics, Hospital for Sick Children, University of Toronto, Toronto, ON, Canada; ^8^McMaster University, Hamilton, ON, Canada; ^9^CHUM, Hôpital Notre-Dame, Montréal, QC, Canada; ^10^CHU Sainte-Justine, Department of Allergy and Immunology, Montréal, QC, Canada

###### **Correspondence:** Mathieu Simard

*Allergy Asthma Clin Immunol* 2020, **16(Suppl 1):**#82

**Background:** Atopic dermatitis (AD) is characterized by a damaged skin barrier that allows allergens to penetrate the body, leading to a sensitization and a higher risk to develop food allergy (35%), asthma (50%), and/or allergic rhinitis (75%), all characterizing the allergic march. This study aims to prevent these diseases by building a polygenic risk score (PRS) to identify newborns with a higher risk of developing AD in the Canadian population.

**Methods:** The Saguenay–Lac-Saint-Jean (SLSJ) asthma cohort and the CHILD birth cohort, which includes respectively imputed GWAS data from 1200 French-Canadian individuals and 5300 individuals from Vancouver, Edmonton, Winnipeg and Toronto, will be used for analyses. Using the two cohorts will increases the power of analysis and will consider the ethnic variability of the Canadian population. Loci associated with AD in the literature will be tested with a general regression model and best associations will be included in the PRS. Patients PRS’ will be calculated according to the odds ratio and the number of risk allele(s) for selected loci. A ROC curve will be built to determine the cut-off threshold of the PRS and analyses will be ran on the risk categories and random samples, tested with and without potential covariates, to assess its sensitivity and specificity.

**Results:** Preliminary results using the SLSJ cohort were obtained from 17 genome-wide loci involved in AD. A PRS including phenotypic data (age, asthma) as covariates was built, giving a 67% specificity, 74% sensitivity and an area under the curve (AUC) of 0.76. Based on these results, adding the CHILD cohort to the analysis should lead to a more accurate specificity and sensitivity (> 80%).

**Conclusion:** Expected results should allow to discriminate newborns (< 6 months old) that are at risk of developing AD in the perspective to prevent the development of the allergic march diseases.

### #83 Assessing quality of life instruments for patients with nasal polyposis: a systematic review

#### Melanie M. Wong^1^, Paul K Keith^2^

##### ^1^Faculty of Health Sciences, McMaster University, Hamilton, ON, Canada; ^2^Department of Medicine, McMaster University, Hamilton, ON, Canada

###### **Correspondence:** Paul K Keith

*Allergy Asthma Clin Immunol* 2020, **16(Suppl 1):**#83

**Background:** Nasal polyposis (NP) is an inflammatory chronic disease of the upper respiratory tract that significantly impacts quality of life (QoL) and daily functioning. Many QoL measurement instruments for NP patients exist; however, it is unclear whether the most disease-specific instrument is currently in use.

**Methods:** A systematic review was conducted in the Ovid MEDLINE, Embase, and Cochrane CENTRAL databases to identify all original validation studies that assessed QoL instruments in NP patients. The quality of each instrument was evaluated following the COnsensus-based Standards for the selection of health status Measurement INstruments checklist.

**Results:** A total of 8 validated QoL instruments for NP patients were identified, with the most commonly-used and well-established tool in the literature being the 22-Item Sinonasal Outcome Test. Significant inconsistencies were noted in the way that measurement properties were developed and reported. All questionnaires assessed the QoL in chronic rhinosinusitis (CRS) with and without NP. Four questionnaires specified the percentage of included NP patients, which ranged from 11 to 71%; however, none of the item generation studies focused exclusively on NP patients. This created challenges in understanding if the tool could assess QoL specifically in these subjects. On quality assessment, three instruments scored positive for at least four of the six measurement properties.

**Conclusion:** QoL impairment in patients with NP has been reported to be prevalent and severe. While a handful of instruments exist to assess QoL in these patients, most of the existing questionnaires lacked a thorough CRS subtype analysis within the sample included in the original validation study. Instruments previously developed to assess QoL in NP patients have not exclusively used NP patients in their development and none had a positive score on all quality assessments. A short, validated, more disease-specific QoL instrument would be helpful in assessing new therapies for NP and making decisions regarding clinical management.

## Urticaria/angioedema

### #84 Real world data of Canadians living with hereditary angioedema (HAE): attributes of new medications

#### Jacquie Badiou^1^, Anne Rowe^1^, Tina McGrath^1^, Kristy Brosz^1^, Daphne Dumbrille^1^, Robert Bick^2^, Suzanne M. Kelly^3^, William Yang^4^

##### ^1^Hereditary Angioedema Canada; ^2^ Health Policy Consultant, Markham, ON, ^3^Red Maple Trials Inc., Ottawa, ON; ^4^Ottawa Allergy Research Corporation, Ottawa, ON

###### **Correspondence:** Jacquie Badiou

*Allergy Asthma Clin Immunol* 2020, **16(Suppl 1):**#84

**Background:** Hereditary angioedema (HAE) is a chronic spontaneous life-threatening disease. Until recently, treatment options for HAE have been limited and required infusion. New treatment options would be beneficial to this population.

**Methods:** In 2019, HAE Canada conducted an online survey of patients and caregivers to assess the challenges patients and caregivers face as a result of hereditary angioedema and to gain insight into their experience and expectation with therapies used to treat hereditary angioedema.

**Results:** Of 73 respondents to the questions, 68 were living with HAE and 6 were providing care to a patient with HAE. Of the respondents, 43/50 (86%) indicated that access to new treatments is extremely important. Attributes for new medications that were considered extremely important were: more convenient dosing interval -38/58, reduction in edema attacks—47/57, easier mode of delivery 44/58, prophylactic administration—44/56. Eight participants (13%) had received treatment with lanadelumab a newly approved medication. On a scale of 1 to 5, five participants rated its effectiveness preventing attacks at 5 and 1 each rated it a 3 or 4. Reported adverse events were headache (2/8) and pain at injection site (7/8) scored as tolerable to very tolerable. The majority (5/8) indicated their quality of life while taking lanadelumab to be comparable to normal living. Access to lanadelumab was predominantly through participation in a Canadian clinical trial (5/8), a compassionate access program (1/8) or through private insurance (1/8).

**Conclusion:** The data collected indicates that newer, more effective and more convenient treatments for are wanted by Canadian HAE patients. The newest approved treatment, lanadelumab, which is given by subcutaneous injection every 2 weeks fulfils some of these requirements according to a limited number of patients. It is important that Canadian HAE patients have access to multiple treatments to address the unpredictable nature of this disease.

### #85 Long-term treatment with ligelizumab is well tolerated, demonstrates sustained control and high retreatment efficacy in patients with chronic spontaneous urticaria

#### Gordon Sussman^1^, Marcus Maurer^2^, Jonathan A Bernstein^3^, Ana Maria Giménez-Arnau^4^, Diane Baker^5^, Avantika Barve^6^, Eva Hua^7^, Thomas Severin^8^, Reinhold Janocha^8^

##### ^1^Division of Allergy and Clinical Immunology, University of Toronto, Canada; ^2^Department of Dermatology and Allergy, Charité -Universitätsmedizin Berlin, Germany; ^3^University of Cincinnati College of Medicine and Bernstein Clinical Research Center; ^4^Dermatology Department, Hospital del Mar-Parc de Salut Mar, Universitat Autònoma Barcelona, Spain; ^5^Baker Allergy Asthma and Dermatology Clinic, Portland, OR, USA; ^6^Novartis Pharmaceuticals Corporation, East Hanover, NJ, United States; ^7^Shanghai Novartis Trading Ltd., Shanghai, China; ^8^Novartis Pharma AG, Basel, Switzerland

###### **Correspondence:** Gordon Sussman

*Allergy Asthma Clin Immunol* 2020, **16(Suppl 1):**#85

**Background:** Ligelizumab provides fast onset of action, sustained control and stronger efficacy compared with omalizumab in patients with chronic spontaneous urticaria (CSU) as demonstrated in the core Phase 2b study (NCT02477332). Here, we report retreatment and long-term efficacy and safety of ligelizumab (240 mg q4w) in an open-label, single-arm extension study (NCT02649218) in patients who completed the core study and presented with active disease (7-day Urticaria Activity Score [UAS7] ≥ 12).

**Method:** Patients entering the extension received ligelizumab for 52 weeks and were followed-up for 48-week. UAS7, angioedema occurrence from the Urticaria Patient Daily Diary, 7-day Angioedema Activity Score (ASS7) and safety were assessed during the 52-week treatment period.

**Results:** From the core study, 70.6% (226/320) of patients entered the extension with 88.9% completing the treatment period. Complete response (UAS7 = 0) was sustained throughout the extension with over 50% of patients achieving this at the end of treatment and 75.8% of patients (95% confidence interval [69.9%, 81.3%]) cumulatively experiencing this at least once. Regardless of core study dose received, efficacy was sustained with ligelizumab retreatment during extension. Proportion of patients reporting angioedema decreased from 33.2% at baseline to 10.8% at week 4. Sustained improvements were observed up to Week 52 where AAS7 score was reduced by 86.3% in those with angioedema at baseline. No new or unexpected safety signals were observed. The most frequently reported adverse events (AEs; ≥ 10% in total) were nasopharyngitis (23.0%), headache (11.9%) and upper respiratory tract infection (10.2%). The majority of AEs were mild (44.2%) or moderate (34.1%).

**Conclusions:** A high rate of early onset, complete control of hives and itch, and angioedema was achieved and sustained with ligelizumab 240 mg q4w treatment for 52 weeks in the population studied. In patients initially well-controlled who relapsed following discontinuation of ligelizumab, retreatment is highly effective. Long-term treatment with ligelizumab was well tolerated.

### #86 Real-world treatment patterns and clinical outcomes among patients with hereditary angioedema (HAE) in Canada

#### Joan Mendivil^1^, Maral DerSarkissian^2^, Rose Chang^2^, Emily S. Brouwer^3^, Samira Jeimy^4^, Paul K Keith^5^, Harold Kim^4,5^, Gina Lacuesta^6^, Sahil Narkhede^2,^ Mu Cheng^2^, Mei Sheng Duh^2^

##### ^1^Shire, a Takeda company, Zug, Switzerland; ^2^Analysis Group, Inc., Boston, MA, USA; ^3^Shire, a Takeda company, Cambridge, MA, USA; ^4^Western University, London, ON, Canada; ^5^McMaster University Hamilton, ON, Canada; ^6^Halifax Allergy and Asthma Associates, Halifax, NS, Canada

###### **Correspondence:** Joan Mendivil

*Allergy Asthma Clin Immunol* 2020, **16(Suppl 1):**#86

**Background:** Real-world data on characteristics of patients with HAE C1-INH, frequency of attacks, and HAE treatments are limited in Canada.

**Methods:** A retrospective longitudinal chart review in 12 sites from six countries, including four sites from Canada, was conducted of HAE C1-INH patients ≥ 12 years of age with ≥ 1 HAE-related visit during 2016–2018. Data on HAE attack frequency and treatments were collected and summarized from the first HAE-related visit until the most recent visit. The rate of HAE attacks was calculated by dividing the number of attacks per patient by the total duration of at-risk observation time when eligible attack information was collected.

**Results:** Among the 51 Canadian patients studied, 68.6% were female, 86.3% were Caucasian, and the mean (standard deviation [SD]) age was 21.6 (13.3) years at symptom onset and 30.5 (16.9) years at diagnosis. 96.1% and 3.9% of patients had type I and type II HAE, respectively; 64.7% had family history of HAE. Over a mean (SD) follow-up of 7.0 (4.2) years, 64.7% received long-term prophylaxis (LTP) and 92.2% used on-demand treatment. The frequency of LTP treatments shifted pre-2014 vs. post-2014: androgen therapy (pre-2014: 76%; post-2014: 18%), pdC1-INH (pre-2014: 33%; post-2014: 82%), and antifibrinolytics (pre-2014: 19%; post-2014: 6%). Mean (SD) annual HAE attack rate was higher among patients who did not receive LTP vs. those who did (23.4 [24.4] vs. 14.6 [13.2]) across the follow-up period, and was 17.7 [13.6] pre-2014 and 10.7 [13.3] post-2014 among patients who received LTP.

**Conclusion:** This is the largest chart review of HAE patients in Canada to date. The shift from predominantly androgen therapy to pdC1-INH post-2014 is mirrored by an associated lower annual attack rate. LTP is an important aspect of managing HAE, and more effective or greater use of LTP is needed to reduce attack frequency in the Canadian population.

### #87 Clinical characteristics and management of inducible chronic urticaria in a pediatric cohort

#### Bruce T. Miles^1^, Sofianne Gabrielli^1^, Elena Netchiporouk^2^, Sharon Baum^3^, Greenberger Shoshana^4^, Petra Straubach-Renz^5^, Moshe Ben-Shoshan^1^

##### ^1^Division of Pediatric Allergy and Clinical Immunology, Department of Pediatrics, McGill University Health Center, Montreal, QC, Canada; ^2^Division of Dermatology, McGill University, Montreal, QC, Canada; ^3^Department of Dermatology, Chaim Sheba Medical Center, Tel-Aviv University, Sackler School of Medicine, Tel Hashomer, Israel; ^4^Department of Dermatology, Sheba Medical Center, Ramat-Gan, Israel; ^5^Department of Dermatology and Allergy, Johannes Gutenbery University KöR, Germany

###### **Correspondence:** Bruce T. Miles

*Allergy Asthma Clin Immunol* 2020, **16(Suppl 1):**#87

**Background:** Chronic urticaria (CU) is defined as hives and/or angioedema occurring for at least 6 weeks. Up to 3% of adults and 0.1% of children will present with CU and among them, almost 15% will have hives induced by an identifiable trigger (e.g. cold, heat, exercise) defined as inducible CU (InCU). There is limited data on patients who have InCU. We aimed to assess the demographics and clinical characteristics of pediatric patients with InCU who presented to the Montreal Children’s Hospital (MCH).

**Methods:** Over a 6 year period, children presenting to the allergy clinic at the MCH with CU were prospectively recruited as part of our urticaria registry. A standardized data entry form documenting symptoms and triggers of CU was collected by a physician. A standardized diagnostic test as previously described was used to establish the presence of a specific form of InCU. A follow-up questionnaire was conducted annually to query patients on the management of their CU. Descriptive statistics were used to describe the demographics and clinical characteristics.

**Results:** From 2013 to 2019 at the MCH, 204 patients with CU were recruited. Among these cases, one-fifth (21.1%) were diagnosed with InCU. The median age was 11.16 years (IQR 5.865, 14.485). Among all patients with InCU, 34.9% also had spontaneous CU. Cold and exercise induced (cholinergic) CU were the most common forms (83.7%, 20.9%, respectively), while solar induced affected 9.3% and pressure induced accounted for 4.7% of all InCU cases (some patients had several triggers). Among all cases, 85.78% had spontaneous UC. The majority of InCU cases (86.0%) required antihistamines daily to control their symptoms. 7.0% had required omalizumab, of which 4.7% had both cold and exercise induced CU and 2.3% had pressure induced CU.

**Conclusion:** Cold was the most common trigger of InCU and most patients were effectively managed with antihistamines.

### #88 Remission of acquired angioedema with C1 esterase deficiency from chronic lymphocytic leukemia achieved with ibrutinib therapy

#### Hoang M. Pham^1^, Gordon L Sussman^2^

##### ^1^Department of Medicine, Division of Allergy and Clinical Immunology, McGill University, Montreal, QC, Canada; ^2^Department of Medicine, University of Toronto, TN, Canada

###### **Correspondence:** Hoang M. Pham

*Allergy Asthma Clin Immunol* 2020, **16(Suppl 1):**#88

**Background:** Acquired angioedema with C1 esterase inhibitor deficiency (C1-INH-AAE) is a very rare disorder thought to be due to malignant overconsumption of C1-INH or anti-C1-INH autoantibody formation. Some attacks of angioedema in C1-INH-AAE may become resistant to C1-INH concentrate, requiring higher doses or alternative treatments like icatibant. Rituximab and chemotherapy for lymphoproliferative disorders have been associated with C1-INH-AAE remission. Here we describe a case of C1-INH-AAE remission with ibrutinib—a potent tyrosine kinase inhibitor of Bruton tyrosine kinase (BTK)—in context of chronic lymphocytic leukemia (CLL) treatment.

**Case presentation:** A 66 year old with a medical history relevant for Del11q CLL, hypothyroidism, and secondary hypogammaglobulinemia was initially referred for laryngeal angioedema provoked by dental cleaning complicated by subsequent attacks of recurrent facial angioedema, in the absence of a family history of angioedema. Her initial labs showed a low C4 and C1-INH in December 2015 but normal CH50. She also had low C1q. She was treated with fludarabine, cyclophosphamide, and rituximab for 2 cycles in December 2016 but this was stopped due to prolonged pancytopenia with a recurrence in April 2017. She was then started on ibrutinib as salvage therapy in May 2017. By July 2017, she was in clinical remission and she had biochemical normalization of C4 & C1-INH antigen.

**Conclusions:** This case highlights that C1-INH-AAE remission may be achieved with treatment of the underlying lymphoproliferative disorder. To the best of our knowledge, this is the first reported case of ibrutinib therapy in CLL which helped a patient achieve remission from C1-INH-AAE. Ibrutinib merits further prospective studies in the management of C1-INH-AAE.

### #89 Natural history, prognostic factors and burden of disease in chronic urticaria

#### Peter Stepaniuk, Manstein Kan, Amin Kanani

##### Department of Medicine, Division of Allergy and Clinical Immunology, University of British Columbia, Vancouver, BC, Canada

###### **Correspondence:** Peter Stepaniuk

*Allergy Asthma Clin Immunol* 2020, **16(Suppl 1):**#89

**Background:** Although the diagnosis and management of chronic urticaria is well documented in the literature, other aspects of the condition including natural history, prognostic factors and humanistic burden is unclear.

**Methods:** This was a prospective, cross-sectional analysis at an allergy referral clinic in Vancouver, British Columbia. We performed a chart reviewed of patient medical records and conducted a survey in patients identified with chronic urticaria to evaluate various aspects of the disease.

**Results:** 72 eligible patients completed the survey. Median age of onset of chronic urticaria was 43 ± 17 years with a median duration of symptoms lasting 57 (range 2–480) months. 31% of patients reported a recurrence of their disease after initial remission with a median length of remission lasting 54 (range 9–252) months. 41 patients (57%) had associated angioedema with a median duration of symptoms lasting 60 (range 2–275) months as compared to 48 (range 3–420) months in those without angioedema. 14 patients (19%) had co-existing thyroid and/or autoimmune disease with a median duration of symptoms lasting 60 (range 2–480) months compared to 57 (range 3–336) months in those without co-existing disease. Three-quarters (75%) of patients reported disrupted sleep due to their urticaria and 29 patients (43%) required emergency room visits for symptomatic relief.

**Conclusions:** The natural history of chronic urticaria in our study population was nearly 5 years with one-third of patients having a relapsing/remitting course. However, when compared to the overall population with chronic urticaria, our study population would be expected to have a more severe disease course as we are a referral centre. Patients with concurrent angioedema or co-morbid thyroid/autoimmune disease had a slight trend towards longer disease duration. We observed a relatively high humanistic burden of disease with significant utilization of health care resources for patients with uncontrolled symptoms.

